# XFM and HERFD-XAS studies of selenium in tissues and whole blood from mice supplemented with potentially therapeutic selenocompounds

**DOI:** 10.1080/13510002.2026.2626151

**Published:** 2026-02-16

**Authors:** Ani T. Baker, Tamara Ortiz-Cerda, Kangzhe Xie, Jordan Hunter, Iliya Dragutinovic, Jonathan C. Morris, Linda I. Vogt, Graham N. George, Dimosthenis Sokaras, Daryl L. Howard, Paul K. Witting, Hugh H. Harris

**Affiliations:** a Discipline of Chemistry, The University of Adelaide, Adelaide, Australia; b Departamento de Citología e Histología Normal y Patológica, Facultad de medicina, Universidad de Sevilla, Seville, Spain; c Redox Biology Group, Charles Perkins Centre, School of Medical Sciences, Faculty of Medicine and Health, The University of Sydney, Sydney, Australia; d School of Chemistry, Faculty of Science, Kensington, UNSW Sydney, NSW, Sydney, Australia; e Department of Geological Sciences, University of Saskatchewan, Saskatoon, Canada; f Stanford Synchrotron Radiation Lightsource, SLAC National Accelerator Laboratory, Menlo Park, CA, USA; g Australian Synchrotron, ANSTO, Clayton, Australia

**Keywords:** Selenium, nanoparticles, selenoneine, dietary supplement, antioxidant, therapeutic, spectroscopy, fluorescence

## Abstract

**Background:**

Selenium nanoparticles (SeNPs) and selenoneine (SeN) are prospective nutritional supplements and therapeutic agents postulated to counteract Se deficiency and alleviate oxidative stress induced by various pathological conditions.

**Methods:**

X-ray fluorescence microscopy (XFM) and high energy resolution fluorescence detected X-ray absorption spectroscopy (HERFD-XAS) investigated Se distribution and speciation in mice fed supranutritional doses of SeNPs (10 14;mg Se/kg in feed) and SeN (5 14;mg Se/kg in feed).

**Results:**

Mice fed control and SeNP diets showed equivalent Se distributions in the liver and testes, however, Se concentrations were elevated in the renal tubules of mice supplemented with SeNPs. Negligible differences in the composition of selenospecies were detected in tissues from mice fed SeNPs and control diets. Mice supplemented with SeN exhibited significant elevations in Se across all analysed tissues. Se Kα_1_ HERFD-XAS data revealed reduced SeN as the predominant selenospecies in tissues and blood from mice fed the SeN diet. Characterisation of the SeN-enriched feed showed that SeN was initially present in both the oxidised dimer and reduced monomer forms.

**Conclusion:**

These results support the hypothesised *in vivo* reduction of SeN and subsequent bioaccumulation in the blood and various tissues. The present findings highlight marked differences in the bioavailability and utilisation of these purported therapeutic selenocompounds.

## Graphical abstract

**Figure:**
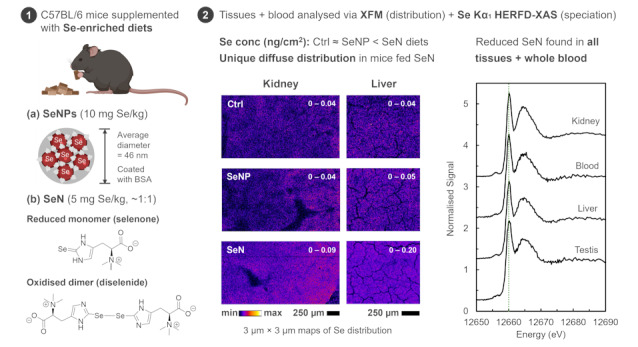

## Abbreviations

ANOVA: Analysis of variance
BSA: Bovine serum albumin
Cys: Cysteine
CysSe^−^: Deprotonated selenocysteine
CysSeH: Protonated selenocysteine
CysSeSeCys: Selenocystine
CV: Central vein
EDTA: Ethylenediaminetetraacetic acid
ET: Ergothioneine
GPx: Glutathione peroxidase
GSH: Glutathione
GST: Glutathione S-transferase
GSSeSG: Selenodiglutathione
HERFD-XAS: High energy resolution fluorescence detected X-ray absorption spectroscopy
ICP-MS: Inductively coupled plasma mass spectrometry
IT: Interstitial tissue
MeHg: Methylmercury
MeSeCys: *Se-*methylselenocysteine
MeSeN: *Se*-methylselenoneine
NAFLD: Non-alcoholic fatty liver disease
OCT: Optimal cutting temperature (compound)
OCTN1: Organic cation/carnitine transporter 1
PV: Portal vein
RML: Right medial lobe
ROI: Region of interest
Sec: Selenocysteine (amino acid code)
SeCys: Selenocysteine
SelP: Selenoprotein P
SeN: Selenoneine
SeNP: Selenium nanoparticles
SeO_3_^2−^: Selenite
SeMet: Selenomethionine
Se2U: 2-selenouridine
ST: Seminiferous tubules
STS: Selenotrisulfide
TEM: Transmission electron microscopy
TrxR: Thioredoxin reductase
XANES: X-ray absorption near edge structure
XAS: X-ray absorption spectroscopy
XFM: X-ray fluorescence microscopy

## 1. Introduction

### 1.1. Selenium and human health

Selenium (Se) is an essential micronutrient required for optimal human health due to its direct relevance to vital biological functions in mammals including the modulation of cellular redox chemistry, antioxidant radical scavenging activities, and thyroid hormone regulation [1,2]. These key functionalities are afforded by the enzymatic roles of selenoproteins, including glutathione peroxidases (GPxs), thioredoxin reductases (TrxRs) and iodothyronine deiodinases, which incorporate Se as selenocysteine (SeCys or Sec), the 21st amino acid [1,3,4]. These examples are among the current 25 identified selenoproteins in the human proteome which are comprehensively described in existing reviews [5,6]. In addition, numerous studies have demonstrated that Se and selenoproteins have fundamental roles in immune function [4,7,8], and both male and female reproductive development and fertility [4,9,10].

Crucially, a growing body of research describes the complex and irregular biological activities and patho-physiological effects of Se consumption outside of its narrow and parabolic optimal dose window [1,4], with further variability exhibited from ingestion of Se in different chemical forms [11–13]. Food sources containing Se include a variety of cereals, nuts and grains, cruciferous and root vegetables, eggs, lean meats and poultry, and seafoods [4,14,15]. These sources provide dietary Se in both inorganic forms, selenite [Se(IV)] and selenate [Se(VI)] (present in minor proportions in food and drinking water), and organic forms, including SeCys, *Se*-methylselenocysteine (MeSeCys), and selenomethionine (SeMet) [4,16,17]. According to current Australian and New Zealand health guidelines, the recommended Se intakes are 60 14;µg/day for adult women and 70 14;µg/day for adult men, with a suggested upper limit of 400 14;µg/day [18]. However, guidelines provided in other countries are inconsistent and the global availability of Se varies substantially due to dramatic fluctuations in the Se content of soils (0.005–5,000 14;mg/kg) between geographic locations with other aspects of the soil composition (i.e. pH, salinity, organic content) largely influencing Se bioavailability and chemical form [13,19–22]. This disparity impacts the Se content of crops and subsequent accumulation and bioconversion throughout the food chain, contributing to the inconsistent worldwide Se intake which ranges from ≤ 10 to ≥ 5,000 14;µg/day [2,23].

Excess Se consumption can induce selenosis (Se toxicity) [19,24] and has been linked to alopecia and dermatitis [25], increased prostate cancer risk [26], type 2 diabetes [27], and an increase in all-cause mortality risk [28,29]. Likewise, low Se status has significant adverse health effects, including diminished immune function [8], thyroid autoimmune disease [30], infertility [31], cognitive decline and neurodegenerative disorders [32,33], alongside ties to increased risks of prostate cancer [34], type 2 diabetes [35], and all-cause mortality [28]. Severe Se deficiency is closely associated with Keshan disease (cardiomyopathy) and Kashin-Beck disease (osteoarthropathy) which affect populations living in regions of endemic Se scarcity [19,36,37]. Collectively, this knowledge has sparked extensive efforts to develop strategies for biofortification of Se in crops [38,39], alongside new avenues for Se-containing nutritional supplements and therapeutics, to reduce the prevalence of diseases associated with Se deficiency. Notably, the detrimental effects of excess Se have highlighted the importance of understanding baseline Se serum status and genetic factors which grant variable Se tolerability [12,40], both of which must be considered to avoid unnecessary Se supplementation and the potential harmful consequences of inaccurate worldwide public health policies.

### 1.2. Elemental Se as a nutritional supplement and therapeutic agent

Se nanoparticles (SeNPs) have emerged as a candidate for dietary Se supplementation with lower acute toxicity relative to other selenocompounds including selenite, SeMet and MeSeCys [41–43]. Beyond reduced toxicity, recent studies have demonstrated that SeNPs can effectively alleviate oxidative stress in mice through the upregulation of antioxidant enzyme activities including selenoproteins (GPxs and TrxRs) [42,44], along with superoxide dismutase, catalase and glutathione S-transferase (GST) [45–47]. These outcomes have guided efforts to investigate the efficacy of SeNPs as chemopreventative and chemotherapeutic agents [48–50]. Furthermore, SeNPs have been postulated as antimicrobial agents [51–53], and as therapeutics for detoxifying heavy metals given recent findings which suggest that SeNPs are capable of mitigating oxidative damage induced by chromium (Cr) [54,55], cadmium (Cd) [56], and mercury (Hg) [57], in poultry and fish. Moreover, results from a recent clinical trial have demonstrated that participants supplemented with SeNPs (200 μg Se/day for 12 weeks) exhibited a small but clinically relevant improvement in biomarkers associated with rheumatoid arthritis, with no adverse side effects [58].

Previous work has been conducted to optimise both chemical synthesis and stability of SeNPs for biological studies. Dispersion of elemental Se in bovine serum albumin (BSA) has been shown to regulate aggregation and inhibit the formation of bulky particles, instead permitting production of Se-coated nanoparticles [44,59], with tuneable size based on the molar ratio of inorganic Se and the reductant, and the agitation speed during synthesis [60]. Importantly, the BSA stabilising agent does not illicit a toxic biological response [60], making it suitable for SeNP supplementation in cells and animal models.

Early studies of BSA-stabilised SeNPs suggested that nanoparticles at variable sizes, between 5–200 14;nm, exhibited comparable bioavailability and equivalent capacities to enhance expression of selenoenzymes (GPxs and TrxRs) in human hepatoma cells and mouse liver [44,61,62]. Simultaneously, smaller SeNPs (∼5–15 14;nm) were shown to be capable of scavenging free radicals with greater efficiency than larger nanoparticles (∼20–200 14;nm) *in vitro* [61]. In recent decades, advancements in the development of nanoparticulate drug delivery systems have highlighted the sensitive physico-chemistry of nanoparticles which can be disrupted *in vivo*, influencing interactions with biological molecules and altering bioavailability [63]. This is supported by subsequent studies that demonstrate a correlation between smaller sized SeNPs and greater Se accumulation in the liver [45], and reduced bioactivities of larger SeNPs with nanorod-like morphology [59]. Hence, both the physical properties and surface chemistry of SeNPs are tightly linked to the efficacy of SeNPs as a nutritional supplement or therapeutic agent.

### 1.3. Discovery and potential therapeutic properties of selenoneine

Alongside SeNPs, there has been a growing interest in the bioavailability and redox properties of selenoneine (SeN, 2-selenyl-*N*α,*N*α,*N*α-trimethyl-l-histidine), a naturally occurring organic form of Se first identified in 2010 in the blood of bluefin tuna (*Thunnus thynnus*) [64,65]. In 2019, SeN was discovered as the major Se species in beluga skin (median 1.8 14;µg Se/g wet weight, 54% total Se content) and the red blood cells of members of the Nunavimmiut population (median 413 14;µg Se/L, 54% total Se content) [66]. Following this, SeN was identified in tissues and blood samples obtained from giant petrels (*Macronectes* sp.) inhabiting the Southern Ocean [67], further demonstrating the trophic bioaccumulation of this organic Se compound. These studies have highlighted the potential association between SeN and methylmercury (MeHg) detoxification, due to the observed inverse relationship between SeN and Hg concentrations in the livers of the giant petrels [67], and the detection of HgSe granules in the organs and tissues of marine organisms and seabirds [68,69].

SeN is the Se analogue of ergothioneine (ET), which is a sulfur-containing derivative of *l*-histidine primarily found in mushrooms, fermented foods, beans and oats [70–72]. Initial assays demonstrated that SeN has impressive free radical scavenging capabilities which are comparable to those of ET [65], highlighting SeN as a potentially valuable Se supplement for antioxidant support, and as a chemopreventative agent and therapeutic for the treatment of chronic immunodeficiency diseases. The capabilities of SeN for efficient protection against oxidative stress are further exemplified in a recent study by Seko *et al*. which reported increased cell viability in cultured human leukemic cells treated with 10 14;µM SeN in the presence of a variety of radical species [73].

At present, studies of SeN in mammalian systems utilising chromatography and mass spectrometry techniques have identified *Se*-methylselenoneine (MeSeN) as a methylated metabolite of SeN in human urine and blood samples [74,75]. Further studies of isotopically labelled SeN in mice have revealed the presence of MeSeN in the blood plasma, liver and kidneys, however, this methylated species was not detected in the brain, spleen and erythrocytes [76]. While these findings and the described accumulation of SeN in marine organisms and human blood are valuable discoveries, the biological activities of SeN *in vivo* remain underexplored.

### 1.4. Evaluation of the distribution and Se speciation in animals supplemented with SeNPs and SeN

In contrast to many hyphenated analytical techniques, synchrotron radiation-based X-ray methods, including X-ray fluorescence microscopy (XFM) and high energy resolution fluorescence detected X-ray absorption spectroscopy (HERFD-XAS), can evaluate *in situ* elemental distribution and speciation, with minimal sample preparation or chemical pre-treatment. Minimising sample processing better preserves sample integrity and maintains the analyte native state, translating to analytical insights which more closely represent *in vivo* (bio)chemistry. The utility of combined XFM and XAS techniques has been demonstrated in previous studies of Se biodistribution and metabolism in cells [77–80] and animals [81,82]. Additionally, the enhanced spectroscopic resolution of HERFD-XAS has been shown to provide significant advantages in the investigation of low-concentration metals and metalloids in biological samples [82–85].

Herein we describe the combined application of XFM and HERFD-XAS to investigate the distribution and speciation of Se in mice fed diets supplemented with SeNPs and SeN, examining the *in situ* fates of these selenocompounds. These Se-enriched diets were formulated to provide mice with supranutritional doses of Se, relative to standard rodent chow (∼0.3 14;mg Se/kg, meeting minimum dietary intake requirements), with feed containing 10 14;mg Se/kg or 5 14;mg Se/kg as SeNPs or SeN, respectively. This concentration of elemental Se lies below the median lethal dose (LD_50_) for orally administered SeNPs (71.1–131.1 14;mg Se/kg with 95% confidence) [42,43,86], indicating minimal risk to induce acute toxicity. At present, SeN is presumed to be non-toxic with no data describing acute or chronic toxicity, however, the provided concentration within the feed lies well below the LD_50_ value of synthetic ET (the sulfur analogue of SeN) which is postulated to be greater than 2,000 14;mg/kg by weight [87]. At these sub-toxic doses, we evaluated Se distributions in kidney, liver and testes tissues via XFM and collected Se Kα_1_ HERFD-XAS spectra to elucidate the composition of selenospecies in these tissues and whole blood. Collectively, we identified that supplementation with SeNPs yielded negligible changes to the native Se distribution and speciation, however, SeN was observed to accumulate in its reduced form in all mouse tissues and whole blood, supporting previous reports of trophic transfer and bioaccumulation [66,67].

## 2. Materials and method

### 2.1. Preparation of Se compounds

#### 2.1.1. Synthesis of SeN

Selenoneine (SeN) was synthesised following the protocol reported by Lim *et al.* [88], with minor modifications to the reaction conditions. Detailed synthetic procedures are provided in the Supplementary Information (SI pages 2–4, reaction schemes illustrated in Figures S1–S5). All spectroscopic data collected here matched data reported in the literature (see ^1^H NMR spectra in SI Figures S6–S9).

#### 2.1.2. Synthesis of BSA-coated SeNPs

SeNPs coated with bovine serum albumin (BSA, A2153, Sigma-Aldrich, St. Louis, MO, USA) were chemically synthesised according to a one-pot procedure previously described by Chung *et al.* [60]. Briefly, ten volume equiv of a 50 14;mM aqueous solution of ascorbic acid (AL022, ChemSupply, Gillman, SA, Australia) was added dropwise to one volume equiv of an aqueous solution containing 100 14;mM sodium selenite (1:5 molar ratio of sodium selenite to ascorbic acid) and 10 14;mg/mL BSA, stirring at 600 14;rpm. Solutions were prepared using ultrapure water from a Milli-Q water system (Millipore-Waters, Lane Cove, NSW, Australia). After complete addition of ascorbic acid, the solution was stirred at 600 14;rpm for 30 mins, then the nanoparticles were collected by centrifugation at 10,000 14;rpm (20 °C, 20 14;min). The supernatant was removed, and the nanoparticles were soaked in ultrapure water (Milli-Q) overnight. Nanoparticles were then rinsed twice with ultrapure water (Milli-Q) via resuspension and centrifugation, then lyophilised at 233 14;K overnight to yield BSA-coated SeNPs as a fine dark red powder.

#### 2.1.3. Characterisation of Se nanoparticles

BSA-coated SeNPs (one microspatula) were dissolved in deionised water (∼1.5 14;mL) and sonicated for 5 14;min then loaded onto a 300-mesh copper-coated carbon film grid (Product 01843, Ted Pella) which was initially glow-discharged using a Quorumtech GloQube Plus Glow Discharge System (Quorum, San Jose, CA, USA) at 20 14;mA for 45 s to increase the hydrophilicity of the surface. The coated grid was air-dried for 20 14;min before imaging by transmission electron microscopy (TEM) using an FEI Tecnai G2 Spirit (Field Electron and Ion Company, Hillsboro, OR, USA) operating at 120 14;kV. Collected TEM images were processed in Fiji ImageJ (Version 1.54 14;g) to verify the average diameter of the SeNPs (SI pages 7–8 and Figure S10).

### 2.2. Preparation of Se-enriched diets

#### 2.2.1. Se-enrichment of dry powder

BSA-coated SeNPs (20.0 14;mg) or SeN (39.6 14;mg) were transferred into a mortar, cleaned with ethanol and air-dried, and gradually combined with a modified Semi-Pure 93M Dough Form Diet (20 14;g, SF23-021, Specialty Feeds, Glen Forrest, WA, Australia), stirring and grinding with a pestle for ∼10 14;min to create a concentrated powder (1,000 14;mg Se/kg or 500 14;mg Se/kg for SeNP or SeN, respectively) for further dilution. In a conventional 4.7 L electric stand mixer with a stainless-steel mixing bowl and non-stick whisk attachment sterilised with ethanol, the Se-enriched powder was diluted 1:10 with additional Semi-Pure feed (180 14;g, for a total of 200 14;g powder containing 100 14;mg Se/kg or 50 14;mg Se/kg, respectively) mixing on low speed for ∼3 14;min. The sides of the mixing bowl were then scraped and the powder was briefly stirred by hand then mixed for a further ∼5 14;min on medium speed. Further gradual additions of Semi-Pure feed, 4 × 200 14;g followed by 2 × 500 14;g, were made following the same homogenisation procedure as described above to give a final concentration of 10 14;mg Se/kg as SeNPs and 5 14;mg Se/kg as SeN in 2 14;kg of Semi-Pure feed powder. For each Se-enriched diet, a second 2 14;kg batch was prepared identically, providing a total of 4 14;kg per diet for the feeding trial. The control diet was unamended and maintained in its original form as prepared by the manufacturer. Dry powder diets were stored at −30 °C until further use.

#### 2.2.2. Pellet formation

To prepare fresh dough pellets, ultrapure deionised water (Milli-Q) was added to the powder (10 14;mg Se/kg or 5 14;mg Se/kg as SeNPs or SeN, respectively) in a 1:5 (w/w) ratio, kneading until combined to form a soft and pliable dough. Dough was pressed into small pellets (2–3 14;g each) and air-dried at 22 °C in a sterilised fume cupboard overnight. The dried pellets were stored at −30 °C until use. Dough form feed was prepared from the dry powder weekly to replenish supplies for the animal feeding trial.

#### 2.2.3. ICP-MS analysis

To assess the homogeneity of Se incorporated into the feeds, Se-enriched dry powder diets (*n* = 4) and dough pellets (*n* = 8, 2 x half segments from 4 x pellets) from the first 2 14;kg batches were digested in 70% HNO_3_ (2 14;mL) at 60 °C for 2 14;h. Solutions were diluted 1:25 (v/v) with ultrapure deionised water (Milli-Q) and filtered through a 0.22 μm syringe filter. Samples were stored at 4 °C until analysis by inductively coupled plasma mass spectrometry (ICP-MS, Agilent 8900x QQQ-ICP-MS) to determine Se concentration. Control dry powder and dough pellets were analysed and spiked with 100 14;µg Se/kg to assess recovery and determine the potential influence of matrix effects (SI page 10 and Table S1).

### 2.3. Rodent feeding trial

Animal studies were conducted in compliance with the ARRIVE guidelines of animal experimentation and the Australian Code for the care and use of animals for scientific purposes, with protocols and conditions approved by The University of Sydney’s Animal Ethics Committee (Protocol #1775, 2020). Male C57BL/6J mice aged 6–8 weeks (18–23 14;g) were purchased from the Australian Resource Centre (Perth, WA, Australia), through The University of Sydney’s Laboratory Animal Services (LAS). Animals were housed in environmentally enriched ventilated cages in the Charles Perkins Centre (CPC) at The University of Sydney. Mice were acclimated for one week before starting the supplementation. At the onset of the experiment, mice were tail-marked for individual identification, randomly assigned to dietary groups, and housed in cages containing three mice (6 x cages, 2 x cages per group, for a total of *n* = 6 animals per dietary group). This sample size aligns with previous studies of the acute toxicity of SeNPs at non-lethal doses in mice [42,43] and conformed to the range of animals (*n* = 4–8) selected in our prior studies of Se uptake in rodents [81,82,89]. Animals were housed on a 12 14;h light/dark schedule with cages maintained at 22 ^◦^C (relative humidity not recorded). The mice were provided with clean drinking tap water *ad libitum* and fed a standard rodent chow diet during the acclimation period.

After acclimation, mice were supplemented with diets derived from the modified Semi-Pure SF23-021 feed; (i) control diet (no added Se, estimated ∼0.3 14;mg Se/kg according to nutrient data provided by the supplier), (ii) SeNP diet (containing 10 14;mg Se/kg), and (iii) SeN diet (containing 5 14;mg Se/kg), for a period of three weeks. Animals were monitored twice weekly to record body weight and the mass of feed consumed per cage. Mice from all groups showed steady weight gain and consumed comparable amounts of feed (3–4 14;g per day) throughout the feeding trial (SI Figure S11), with no significant adverse health effects reported for either Se-enriched diet. Any uneaten food was removed and replaced with fresh pellets twice weekly.

### 2.4. Blood and organ collection

At the end of the three-week feeding period, the male mice were humanely sacrificed according to approved ethics protocols. Briefly, mice were fully anaesthetised with 4% v/v inhaled isoflurane. Blood was extracted via cardiac puncture and drawn into microfuge tubes coated with ethylenediaminetetraacetic acid (EDTA, 100 14;µL of 1 14;mM EDTA stock for tube coating, E4884, Sigma-Aldrich) and stored at −80 °C for subsequent analysis. Euthanasia was confirmed by cervical dislocation. Next, the abdomen was opened transversely and the kidneys, whole liver and separated right medial lobe (RML), and testes were harvested and snap frozen in liquid nitrogen (LN_2_). Samples were stored in cryotubes at −80 °C until further sample preparation.

### 2.5. X-ray fluorescence microscopy

Frozen tissues (kidney, liver RML, and testis) were partially defrosted and immersed in Tissue-Tek Optimal Cutting Temperature compound (OCT, ProSciTech, QLD, Australia) in plastic moulds and frozen in LN_2_. OCT-embedded tissues were stored at −80 °C prior to sectioning. Tissues were cryo-sectioned (25 μm) and mounted onto 5 14;mm × 5 14;mm × 500 14;nm silicon nitride windows (Silson, England), as described elsewhere [90], air-dried and stored in the dark at 22 ^◦^C until analysed. Prior to X-ray analysis, optical images of tissues on silicon nitride windows were obtained using a Nikon Ti-ECLIPSE Inverted Microscope (Nikon Instruments Inc., Melville, NY, USA) applying differential interference contrast (DIC) at 10X magnification.

Elemental distribution maps were collected on the X-ray fluorescence microscopy (XFM) beamline at the Australian Synchrotron, Victoria, Australia [91]. The X-ray beam was tuned to an incident energy of 15.8 14;keV using a Si(111) double crystal monochromator and focused to a spot size of approximately 2 μm x 2 μm using Kirkpatrick–Baez mirrors. The fluorescence signal was collected using a three-element Vortex ME3 detector (Hitachi High-Tech, USA) using a XIA FalconX digital signal processor, with a 90° collection angle geometry. Elemental maps for tissue sections (25 μm thick) were collected in fly-scan mode with a 3 14;µm x 3 14;µm pixel virtual step size and 7.5 14;ms per pixel effective dwell time. After initial data collection, acquisition parameters were adjusted to obtain higher resolution maps of smaller regions of interest (ROIs) in the kidney cortex and medulla, collecting elemental maps with a 1 14;µm x 1 14;µm pixel virtual step size and a 10 14;ms per pixel effective dwell time.

In GeoPIXE (Version 7.7f), the fit to a representative fluorescence spectrum was used to generate a dynamic analysis matrix that was used to project the images [92]. Following quantification, elemental maps scaled by concentration (in areal density, ng/cm^2^) were generated as .tiff files for further analysis.

For each tissue, ROIs corresponding to identified structural features were selected and quantified in Fiji ImageJ (Version 1.54 14;g), recording the mean areal density (ng/cm^2^) for each element. Statistical analyses were performed in GraphPad Prism (Version 10.1.0), applying an ordinary one-way analysis of variance (ANOVA) with Tukey’s multiple comparisons test to determine significant differences in elemental concentrations between dietary groups. In some cases, elemental concentrations were compared between two selected regions within the same tissue via a one-way ANOVA with selected pairwise comparisons, applying Sidak’s correction for multiple comparisons. For instances where multiple identical structures were measured within the same tissue (*i.e.* individual glomeruli in kidneys, or several repeating units in testes), the mean of *n* *=* 4 measurements was calculated for each tissue and compared between biological replicates and across each dietary group. For all applied statistical tests, differences were considered significant at *P* < 0.05.

### 2.6. X-ray absorption spectroscopy

High energy resolution fluorescence detected X-ray absorption spectroscopy (HERFD-XAS) experiments were carried out at the undulator beamline 15-2 at the Stanford Synchrotron Radiation Lightsource (SSRL) with the SPEAR3 storage ring containing 500 14;mA at 3.0 14;GeV. The incident beam energy was selected using a liquid nitrogen-cooled Si(311) double crystal monochromator with ∼0.4 14;eV incident X-ray energy resolution at the Se K-edge. Rhodium-coated Kirkpatrick-Baez mirrors upstream of the sample were used to deliver a 90 × 600 μm^2^ beam (full width and half maximum), providing an incident flux of ∼5 × 10^11^ ph/s. High energy resolution X-ray fluorescence from the Se Kα_1_ emission line was recorded using an array of seven Si(844) spherically bent crystal analysers in the Johann geometry [93], providing a Bragg angle of ϑ = 85.2^◦^. Further details of the beamline configuration and mode of calibration are described elsewhere [82].

Samples were prepared as partially defrosted tissues (kidney, liver, testis, and whole blood) packed into plastic cuvettes sealed with Kapton tape. The tissues were refrozen by immersion in LN_2_ and stored at −80 °C until measurement. During analysis the samples were maintained at a temperature of ∼10 14;K using a helium flow cryostat (Oxford instruments, Abingdon, UK). Sample cuvettes were inclined at an angle of 45° to the incident X-ray beam to facilitate measurement of X-ray fluorescence. An in-hutch fast shutter was employed to prevent unnecessary exposure of the sample to the X-ray beam when data was not actively recorded.

Data acquisition was conducted using the SPEC software package (Certified Scientific Software, Cambridge, Massachusetts, USA), and the PyMCA software package [94] was used for visualisation and to help align samples at an incident energy above the absorption edge (12,800 14;eV). The peak centroid of the Se Kα_1_ emission line was selected as the emission energy at which to record HERFD-XAS. For particularly dilute samples (*e.g.* control diet tissues), the mean emission energy of reduced model selenocompounds and previously recorded tissues was selected. To guard against radiation damage, the sample was translated relative to the incident X-ray beam to interrogate a fresh and unexposed spot for each spectrum collected. A single spectrum (with a ∼2-minute scan duration) was recorded at each spot then the sample was repositioned for the next scan. This was repeated sequentially, collecting 12–28 scans per sample.

Calibration, averaging and background subtraction of HERFD-XAS spectra was performed using the EXAFSPAK software package (G. N. George, SSRL, http://www-ssrl.slac.stanford.edu/exafspak.html). Linear combination analysis of X-ray absorption near edge structure (XANES) spectra was performed between 12,640 14;eV and 12,720 14;eV for Se Kα_1_ HERFD-XAS spectra. Components were excluded from the fit whenever the estimated standard deviation (esd), obtained from the diagonal of the variance-covariance matrix, was greater than the reported fit fraction. The total library of model selenocompounds is described in elsewhere [82].

Se Kα_1_ HERFD-XAS spectra of frozen samples of the Se-enriched diets were also analysed to verify the composition of selenospecies in the dough pellets fed to mice (SI pages 10–11, Figures S12-S13 and Table S2).

## 3. Results

### 3.1. Effects of supplementation on total Se content and distribution in tissues

#### 3.1.1. Kidneys

**Figure 1.**
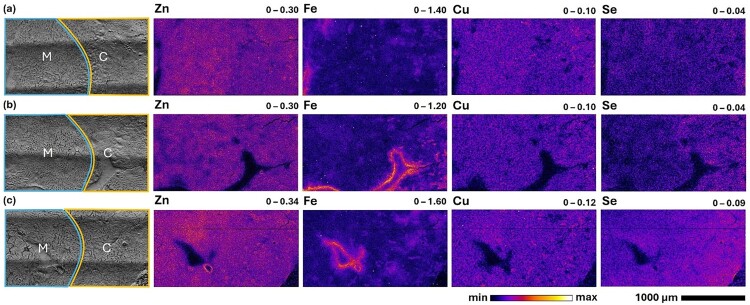
Optical (left) and XFM elemental maps (right, 3-µm resolution) of kidney sections (25 14;µm) from C57BL/6J mice fed the following diets over a 3-week feeding trial: (a) control (∼0.3 14;mg Se/kg),: (b) 10 14;mg Se/kg as SeNPs, and (c) 5 14;mg Se/kg as SeN. Approximate regions representing the renal cortex (yellow box, ‘C’, right-hand side) and medulla (light blue box, ‘M’, left-hand side) are indicated in the optical images corresponding to each kidney section. Minimum and maximum concentrations (in areal density, µg/cm^2^) of each element are displayed at the top right of each elemental map and represent the range of the colour map for each image. XFM images were processed in Fiji ImageJ. A Gaussian blur (σ = 0.25 for Zn and Fe, σ = 0.75 for Cu and Se) and saturation (0.35) were applied to all elemental maps.

The distribution of Se in the kidneys of mice fed the SeNP diet was unchanged relative to the control diet, however, Figure 1 illustrates a marginal increase in Se concentration within the renal cortex (outer portion of the kidney depicted on the right-hand side of each image). In contrast, the kidneys from mice fed the SeN diet show a substantial elevation of Se across the whole tissue, albeit with higher concentrations of Se localised within the renal cortex in this example (Figure 1). The cortical tissue contains a high frequency of glomeruli, which contain a network of capillaries, and are located at the beginning of nephrons, which are responsible for filtering the blood circulating through the kidneys [95–97]. The glomerular filtration rate has been reported to be proportional to the size and length of the proximal convoluted tubules, and this rate is increased in nephrons which lie closer to the corticomedullary border [96]. In addition, kidneys play a key role in the excretion of Se from plasma, with a reported clearance rate of 0.18 14;mL/min [98]. Hence, the general elevation of Se in the renal cortex, diminishing with greater proximity to the medulla, was unsurprising and likely reflects the increased Se concentration within the circulating blood of animals fed Se-enriched diets.

**Figure 2.**
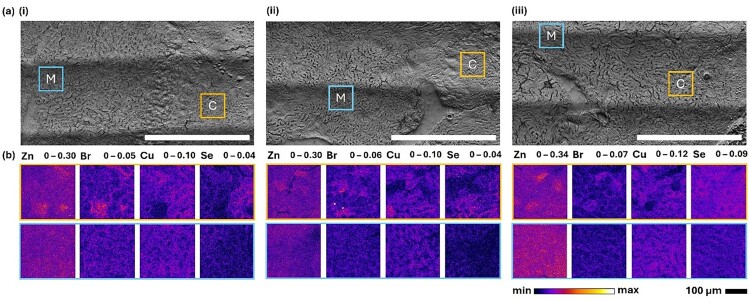
Comparison of cortex (yellow box, ‘C’) and medulla (light blue box, ‘M’) regions in (a) optical images and (b) XFM elemental maps (1-µm resolution) of kidney sections (25 14;µm) from C57BL/6J mice fed the following diets over a 3-week feeding trial: (i) control (∼0.3 14;mg Se/kg), (ii) 10 14;mg Se/kg as SeNPs, and (iii) 5 14;mg Se/kg as SeN. The scale bars on the optical images indicate 1,000 14;µm. The top row of XFM images depicts the cortex regions, and the bottom row corresponds to the medulla regions defined in the above optical images. Minimum and maximum concentrations (in areal density, µg/cm^2^) of each element are displayed at the top right of each elemental map and represent the range of the colour map for each image. These are equivalent to the ranges given for Zn, Cu and Se in Figure 1. XFM images were processed in Fiji ImageJ. A Gaussian blur (σ = 0.25 for Zn, σ = 0.75 for Br, Cu and Se) and saturation (0.35) were applied to all elemental maps.

We have previously demonstrated that the glomeruli can be identified by the colocalisation of Zn and Br, providing a useful landmark for distinguishing the cortical tissue from the medulla [82]. In this study, where rodent tissues were not perfused to remove excess blood from the organs, the Fe maps also provide a clear indication of the location of glomeruli, with the hotspots of Zn aligning with the Fe distribution in Figure 1. The general localisation of Se within the renal cortex is further illustrated in Figure 2, comparing the 1-µm resolution elemental maps of Zn, Br, Cu and Se within smaller areas (250 14;µm x 250 14;µm) of the renal cortex and medulla in tissues from each diet. In these images, Zn and Br were observed in the cortex but absent in the medulla, consistent with glomeruli being absent in the renal medulla. The concentration of Se was distinctly increased in the proximal tubules adjacent to glomeruli. Lower Se concentration was detected within the distal tubules of the renal medulla when comparing kidneys isolated from mice fed the control and Se-enriched diets.

Previous studies of rodents orally supplemented [81] or intraperitoneally administered [82] with selenite have demonstrated a distinct accumulation of Se in the renal cortex and a strong colocalisation of Se and Cu in this tissue subregion. This physiological outcome was also observed in animals treated with non-native selenotrisulfides (STS), representing the downstream metabolites of Se(IV) oxoanions [82]. Distinct association between Se and Cu distributions was not observed in the kidneys of mice fed either the SeNP or SeN diet. Instead, the concentration and distribution of Cu in the kidneys was unchanged and identical to the control tissue (Figures 1 and 2). This further substantiates the hypothesis that the previously observed punctate Se and Cu distribution is a unique result of the metabolic pathway of selenite. Additionally, this observation signifies that there was likely negligible degradation of SeNPs to selenite within the rodent feed, which was further supported by the speciation data obtained for the SeNP diet (SI Figures S12–S13, Table S2).

To further assess the observed trends in the distribution of Se within the kidneys, regions of interest (ROIs) corresponding to (i) total kidney area, (ii) cortex, (iii) medulla, and (iv) glomeruli, were identified and measured to compare mean concentrations of Se across replicate tissues from each dietary group via an ordinary one-way ANOVA and a post-hoc Tukey’s multiple comparisons test. Mean Se concentrations in the total kidney were significantly elevated (up to ∼3.5-fold greater) in mice fed the SeN diet relative to the control and the SeNP dietary groups (*P* < 0.0001) (SI Figure S14, Tables S3–S4). Subsequently, mean Se concentrations were significantly elevated in individually identified cortex and medulla ROIs in kidneys from the SeN dietary group, relative to both the control and SeNP groups (SI Figure S15, Tables S5–S8). This comparison was most significant for the cortex (*P* < 0.0001), but marginally less so for the medulla (*P* = 0.0007) where greater variation in Se concentration was observed.

The pairwise comparison of Se concentrations in cortex and medulla regions (SI Figure S16, Tables S9–S10) further demonstrated this inconsistency in the Se content within the medulla, which fluctuated between biological replicates more substantially than Se within the cortex. Although the mean Se concentration in the cortex was marginally greater than that of the medulla for both the control and SeNP groups, corresponding to the visualised distributions in Figures 1 and 2, this trend was not significant. The difference in the mean Se content between cortex and medulla ROIs was further diminished for the SeN group. Therefore, despite the localisation of Se in the cortex observed in Figure 1, comparison across replicates suggested that the elevation of Se in animals from the SeN dietary group was not tissue specific. Mean Se concentrations within glomeruli (average of *n* *=* 4 individual glomeruli from each tissue) were elevated (up to ∼3-fold greater) in the kidneys of mice fed SeN compared to the control and SeNP dietary groups (*P* < 0.0001) (SI Figure S17, Tables S11–S12). However, unlike previous comparisons, the SeNP diet showed a significant increase in Se concentration in the glomerular capillary tuft relative to the control (*P* = 0.0045), suggesting the potential elevation of Se within the blood of mice supplemented with SeNPs, and hence, some degree of bioavailability and uptake.

The mean concentrations of Cu and Zn were consistent for all ROIs across all groups, with no significant differences in these elemental concentrations between the cortex and medulla ROIs (SI Figures S14–S17, Tables S3–S12). This indicates that the concentrations and distributions of these endogenous elements were not perturbed by dietary supplementation with SeNPs or SeN.

#### 3.1.2. Liver

**Figure 3.**
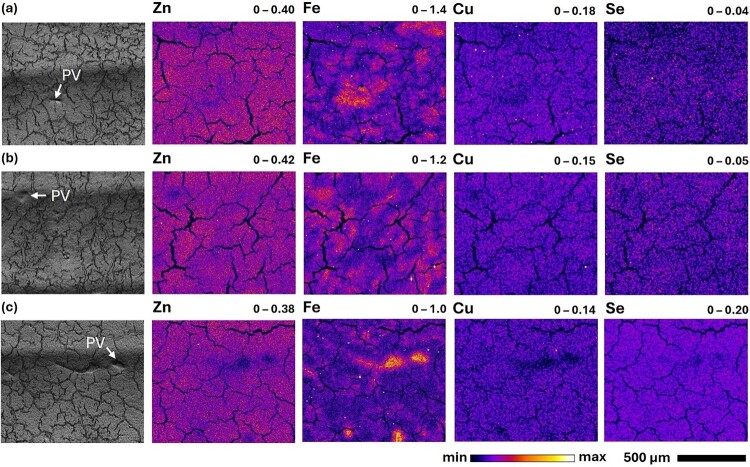
Optical (left) and XFM elemental maps (right, 3-µm resolution) of liver sections (25 14;µm) from C57BL/6J mice fed the following diets over a 3-week feeding trial: (a) control (∼0.3 14;mg Se/kg), (b) 10 14;mg Se/kg as SeNPs, and (c) 5 14;mg Se/kg as SeN. An example of a portal vein (white arrow, ‘PV’) region, representing high density Fe content, is indicated in the optical images corresponding to each liver section. Minimum and maximum concentrations (in areal density, µg/cm^2^) of each element are displayed at the top right of each elemental map and represent the range of the colour map for each image. XFM images were processed in Fiji ImageJ. A Gaussian blur (σ = 0.25 for Zn and Fe, σ = 0.75 for Cu and Se) and saturation (0.35) were applied to all elemental maps.

XFM maps of Se in mouse livers (Figure 3) demonstrate that tissues from the control and SeNP dietary groups exhibit similar concentrations of Se, whereas the livers from mice fed SeN show substantially greater Se signal. This indicates that supplementation of mice with SeNPs had minimal impact on the concentration of Se in the liver, despite the potential marginal increase of Se within the blood inferred from statistically significant comparison of the glomeruli in the kidneys of animals fed control and SeNP diets. In contrast, supplementation with SeN resulted in accumulation of Se within the liver. For all dietary groups, the distribution of Se within mouse liver (right medial lobe, RML) was homogenous with no evidence of localisation within the portal vein (PV, high Fe density) or central vein (CV, low Fe density) regions throughout the repeating subunits of the lobule. Our recent HERFD-XAS studies of Se speciation in rats administered with selenite and STS compounds provided evidence for a metal-selenide metabolite in the blood and several tissues, however, no perturbation of Cu concentration was identified in liver XFM data [82]. In this work, the XFM maps (Figure 3) indicate that the distributions of endogenous Zn, Fe and Cu were consistent across all dietary groups.

To validate these observations, statistical analyses (ordinary one-way ANOVA and post-hoc Tukey’s test) were performed to compare the mean Se concentration in (i) total liver area, (ii) PV, and (iii) CV ROIs across replicate tissues from each dietary group. Mean Se concentrations in the total area of the liver RML were indeed significantly elevated for the SeN group, relative to both the control and SeNP dietary groups (*P* < 0.0001) (SI Figure S18, Tables S13–S14). The passage of nutrient-rich blood from the digestive organs to the liver is mediated by the PVs, with a smaller supply of oxygen-rich blood provided by the adjacent hepatic arteries [99,100]. Thus, hotspots of Fe in the XFM maps, visualised in an approximately hexagonal arrangement (Figure 3), were used to estimate the location of the PVs. The areas between these hotspots were denoted as the location of the CVs where the blood drains into after processing in the liver [99]. Given the observed homogeneity in Se distribution, the elevations in the mean concentrations of Se within the PV and CV ROIs in the liver tissues from the SeN dietary group were equally statistically significant, relative to the control and SeNP groups (*P* < 0.0001 for both ROI comparisons and diets) (SI Figure S19, Tables S15–S18). This homogeneity was further confirmed by the pairwise comparison of the PV and CV ROIs which demonstrated no significant differences in Se concentration between the regions (SI Figure S20, Tables S19–S20).

The mean concentrations of Cu and Fe were consistent across the total area of liver and the individually assessed ROIs for all dietary groups (SI Figures S18–S19, Tables S13–S18). Thus, there was good confidence that the concentrations and distributions of these elements were unaffected by Se supplementation. Pairwise comparisons of mean Fe concentration in the PV and CV ROIs from each dietary group verified that the selected PV ROIs exhibited significantly higher mean concentrations of Fe than the CV (*P* = ≤ 0.0044) (SI Figure S20, Tables S19–S20).

#### 3.1.3. Testes

**Figure 4.**
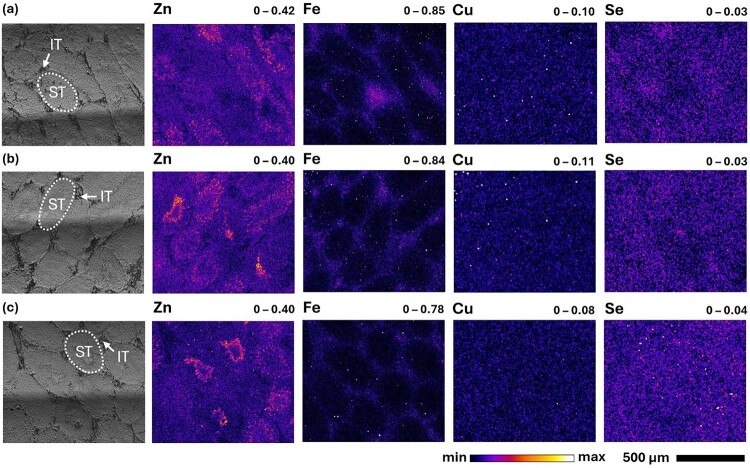
Optical (left) and XFM elemental maps (right, 3-µm resolution) of testis sections (25 14;µm) from C57BL/6J mice fed the following diets over a 3-week feeding trial: (a) control (∼0.3 14;mg Se/kg), (b) 10 14;mg Se/kg as SeNPs, and (c) 5 14;mg Se/kg as SeN. Examples of seminiferous tubule (white dotted oval, ‘ST’) and interstitial tissue (white arrow, ‘IT’) regions are indicated in the optical images corresponding to each testis section. Minimum and maximum concentrations (in areal density, µg/cm^2^) of each element are displayed at the top right of each elemental map and represent the range of the colour map for each image. XFM images were processed in Fiji ImageJ. A Gaussian blur (σ = 0.25 for Zn and Fe, σ = 0.75 for Cu and Se) and saturation (0.35) were applied to all elemental maps.

In the testes of mice fed the control and SeNP diets, XFM maps revealed that the concentration and distribution of Se was identical for animals fed control and Se-enriched diets (Figure 4). In these tissues, Se was observed to be localised within the seminiferous tubules (ST), the repeating functional units of the testes where spermatogenesis occurs [101,102], visualised in the optical images and Zn maps. Specifically, the highest concentrations of Se were found to coincide with regions of high Zn, accumulating outward from the centre of the STs. Zn is abundant in sperm nuclei and is incorporated into several important Zn finger proteins which act as transcription factors during sperm development [103–105]. Additionally, Se enrichment during spermatogenesis is associated with elevated GPx4 expression in late spermatids [105]. Hence, these elements were concentrated in the developing spermatids within the STs, as observed in previous XFM studies [82,105]. Although the tubule structures can be readily identified in the optical and XFM images, the Zn maps highlight the irregularity in the stage of spermatogenesis observed in each tubule within the same organism and between individuals. This has been previously observed in XFM images of rat testes [82], indicating that spermatid formation is not synchronous between each tubule, creating an inherent source of variability which must be considered when comparing these structures in different regions of the same testis. Additionally, the Fe maps provided a useful guide to distinguish the interstitial tissue (IT), which lies between the tubules and contains a diverse collection of cell types critical to testicular function and fertility [102,106]. Unlike Zn and Fe, Cu exhibited a diffuse distribution in this tissue. In contrast to the testes from the control and SeNP groups, Se was elevated in the testes of mice fed SeN. This finding suggests that SeN or its metabolite(s) were partially accumulated within the mouse testes, unlike selenite and STS compounds which demonstrated negligible impacts on the concentration, distribution, and speciation of Se within the testes of rats administered with 1 14;mg Se/kg by weight [82]. Additionally, the example shown in Figure 4 suggests a comparatively uniform diffuse distribution of Se throughout the testes in the mice fed SeN which is consistent with other biological replicates.

Further insights into these observations were derived from an ordinary one-way ANOVA and post-hoc Tukey’s test comparing mean Se concentrations in (i) total testis area, (ii) ST, and (iii) IT ROIs across replicates from each dietary group. Alike comparisons for the kidney and liver, differences in mean Se concentrations between tissues from mice fed the control and SeNP diets were insignificant. For the total testis area, the mean Se concentration was significantly elevated in mice fed SeN (up to ∼2-fold greater), relative to both the control (*P* = 0.0002) and SeNP (*P* = 0.0001) dietary groups (SI Figure S21, Tables S21–S22). The significance of the increase in mean Se concentration in mice fed SeN, relative to control and SeNP diets, was marginally greater in the ST (*P* = 0.0001 for both comparisons) than the IT (*P* = 0.0005 and 0.0003, respectively) (SI Figure S22, Tables S23–S26). Despite this, pairwise comparisons of ST and IT ROIs indicated that this difference was insignificant for all dietary groups, however, the greater overlap of uncertainties and higher variance of the measurements from the SeN group support the observed general uniformity in Se distribution in the testes, relative to the other groups (SI Figure S23, Tables S27–S28).

Statistical analyses of Zn and Fe concentrations indicate that the mean concentrations of these elements were consistent across replicates and between dietary groups (SI Figures S21–S22, Tables S21–S26), indicating no adverse effects from Se supplementation. Pairwise comparisons between ST and IT ROIs (SI Figure S23, Tables S27–S28) showed a general trend of higher mean Fe in the IT (not significant) and higher mean Zn in the ST (significant, *P* = 0.0055 and 0.0216 for the SeNP and SeN dietary groups, respectively), suggesting consistent identification of these ROIs.

### 3.2. Influence of supplementation on Se speciation in mouse tissues and whole blood

**Figure 5.**
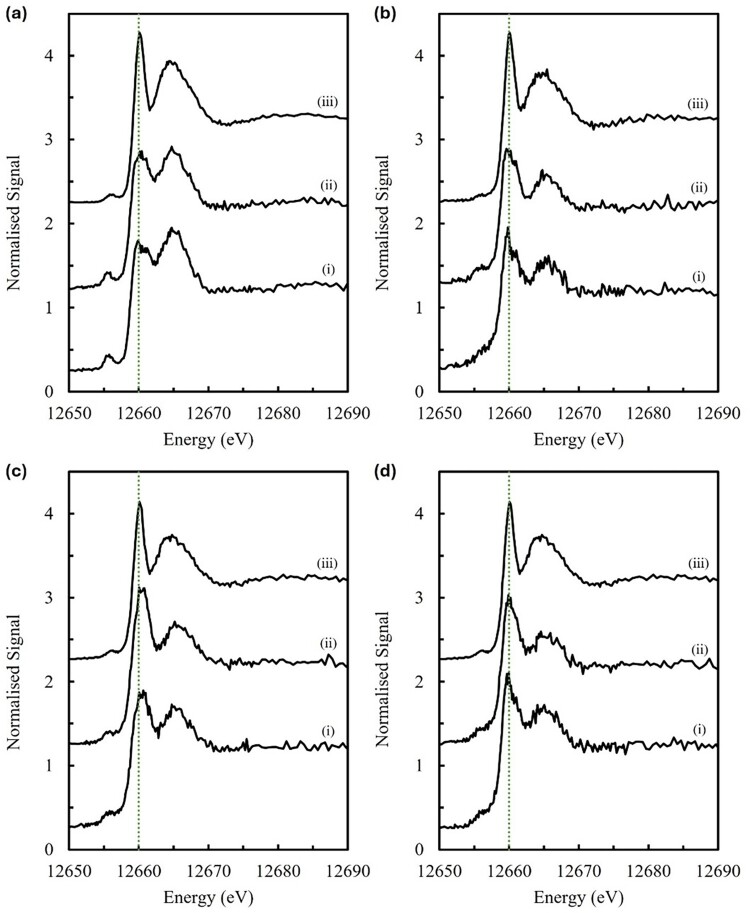
Se Kα_1_ HERFD-XAS XANES spectra of mouse tissues (a) kidney, (b) whole blood, (c) liver, and (d) testis, from dietary groups (i) control, (ii) 10 14;mg Se/kg as SeNP, and (iii) 5 14;mg Se/kg as SeN. Kidney spectra are the average of scans from two biological replicates show in Figure S24. Green dotted lines at 12,660.0 14;eV are included to emphasise differences in the spectral peak positions.

XFM results indicated that the concentration and distribution of Se in the tissues of mice fed SeNPs showed a strong resemblance to the control group, while substantial elevations of Se were observed in the tissues (kidney, liver and testis) from mice fed SeN. Complementary to this data, Se Kα_1_ HERFD-XAS spectra (Figure 5) were collected for these mouse tissues, and for corresponding whole blood samples, to examine the effect of supplementation on Se speciation throughout the organism. Broadly, these results demonstrated that the Se Kα_1_ HERFD-XAS spectra obtained from animals fed SeNP were almost identical to the control group for all analysed tissues and the whole blood. In the whole blood spectra from mice fed SeNPs, the signal-to-noise was marginally improved, potentially indicating an elevation of total Se concentration relative to the control group. In each case, spectra from control and SeNP groups were notably distinct from the spectra obtained from mice fed SeN. Comparing the spectra collected from animals in the control and SeNP groups, the variable intensities of the major peaks at ∼12,660 14;eV and ∼12,665–12,666 14;eV indicate subtle differences in the composition and ratio of selenospecies between diets and between each tissue. In contrast, the spectra of tissues and whole blood derived from animals fed SeN show striking similarity, with a sharper major peak at ∼12,660 14;eV and a taller and broader shoulder peak shifted to slightly lower energy (∼12,664.5 14;eV) relative to the spectra from control and SeNP groups. This uniformity suggests a characteristic and consistent change in Se speciation throughout organisms fed the SeN diet.

Several recent studies have demonstrated that the improved spectroscopic resolution of HERFD-XAS, relative to conventional XAS, provides a meaningful advantage in the quantitative determination of Se and Hg speciation in complex biological samples through linear combination analysis of high energy resolution XANES spectra [82–85]. Our current Se Kα_1_ HERFD-XAS model compound library, described in detail in previous publications [82,83], was utilised to estimate the mixture of selenospecies present in the tissue and whole blood samples from Se-supplemented mice (Table 1).

**Table 1.** Percent Se species in tissues from mice fed the following diets: control, SeNPs and SeN.*^a^* Fit fractions are estimated by a linear combination of model compound spectra.*^b^*

Tissue	Group	Percentage (%) Se species								N_tot_*^d^*	Residual (×10^−3^)
		SeO_3_^2− *c*^	Se (0)	GSSeSG	SeMet	CysSe^−^	CysSeSeCys	CuSe	SeN (R)		
Kidney	Control	8 (1)	8 (1)	–	21 (1)	35 (3)	–	26 (2)	–	0.98	4.04
	SeNPs	9 (1)	11 (1)	–	22 (1)	28 (2)	–	28 (2)	–	0.98	3.12
	SeN	–	–	–	7 (1)	12 (1)	–	6 (1)	74 (1)	0.99	0.80
Blood	Control	3 (1)	6 (2)	–	19 (1)	12 (3)	–	51 (3)	–	0.93	6.82
	SeNPs	4 (1)	10 (2)	–	20 (1)	10 (3)	–	48 (3)	–	0.94	5.83
	SeN	–	4 (1)	–	7 (1)	13 (2)	–	14 (1)	60 (2)	0.97	1.02
Liver	Control	4 (1)	–	5 (1)	24 (1)	20 (3)	–	42 (2)	–	0.96	4.03
	SeNPs	3 (1)	–	11 (1)	26 (1)	20 (2)	–	36 (1)	–	0.98	2.42
	SeN	–	–	–	7 (1)	7 (2)	–	18 (1)	62 (2)	0.94	1.34
Testis	Control	8 (1)	–	–	10 (1)	11 (3)	28 (2)	41 (2)	–	0.98	4.43
	SeNPs	6 (1)	–	–	12 (1)	9 (3)	27 (2)	40 (2)	–	0.93	4.64
	SeN	–	–	–	11 (1)	–	6 (1)	17 (1)	63 (1)	0.97	1.38

*^a^* Se-enriched diets contained 10 14;mg Se/kg and 5 14;mg Se/kg as SeNPs and SeN, respectively. *^b^* Values in parentheses are the estimated standard deviations derived from the diagonal elements of the covariance matrix and are a measure of precision. *^c^* Model spectrum was shifted by −0.25 14;eV to account for the shift between Kα_1_ HERFD-XAS fluorescence lines for selenite (oxidised Se) and selenomethionine (reduced Se). ^*d*^ N_tot_ is the sum of the fractions.

As a brief preface to our discussion of the Se Kα_1_ HERFD-XAS results, we noted that similar residuals were obtained in the linear combination fits to tissue spectra from animals fed control and SeNP diets by including (i) a selenourea (Se(0), Se=C(NR_2_)_2_) model spectrum, represented by selenouridine (Se2U) as the reference compound, or (ii) selenite (Se(IV), pH 5.5) and an alternative Se(0) model spectra (either elemental Se or selenodiglutathione, GSSeSG) (see examples in SI Figure S25 and Table S29). This suggests that the combination of Se(IV) and some Se(0) model spectra produces a similar fit component to the spectrum of Se2U alone. Unlike Se2U, the presence of inorganic selenocompounds in mammalian tissues has been confidently reported [107,108], therefore we propose that the presence of minor quantities of selenite in these tissues, alongside elemental Se and GSSeSG, would be more probable than the presence of a moderate fraction (∼30%) of the selenourea species. Thus, despite the enhanced spectroscopic resolution of HERFD-XAS relative to conventional XAS, the similarity between these fits using different model components further emphasises the complexity of biological spectra and limitations associated with unambiguous metabolite identification via XANES analysis.

Unlike conventional Se K-edge XAS which measures the fluorescence from the sum of all transitions between the higher occupied levels and the core level (2p → 1s), Se Kα_1_ HERFD-XAS measures a smaller subset of transitions (2p_3/2_ → 1s) central to the fluorescence peak [83]. The specific energy varies between samples containing different selenospecies but can be determined using the resonant inelastic X-ray scattering (RIXS) plane – a contour plot of the X-ray emission intensity versus incident energy and the energy transfer (incident energy minus the emission energy). The Se Kα_1_ model spectra for selenocompounds were collected at individually optimised non-resonant emission peak energies. For most compounds with oxidation states between Se(−II) and Se(0), and for biological samples which typically contain greater proportions of reduced Se, these energies are approximately equivalent. However, the energy of the Se Kα_1_ fluorescence line shifts with significant changes in the electronic configuration the element (*e.g.* changing oxidation state from Se(0) to Se(IV)) [83], hence, the individually optimised emission energy of the selenite model spectrum would differ from that of the reduced model selenospecies by a few tenths of an eV, shifting the HERFD-XAS spectrum relative to other models. This was accounted for in our fits using the selenite model spectrum. Since our mouse tissue spectra were collected at emission energies very close to the reduced model compounds (differing by < 0.1 14;eV) and given the absence of significant off-diagonal peaks in the RIXS plane for Se, we utilised the energy difference between Kα_1_ HERFD-XAS fluorescence lines for selenite and SeMet (−0.25 14;eV) to translate the selenite model spectrum. Hence, a shift of −0.25 14;eV was applied to the Se Kα_1_ HERFD-XAS spectrum for selenite in the linear combination fitting of these mouse tissue spectra.

Linear combination fits of tissue spectra showed that animals fed control and SeNP diets had generally similar compositions of selenospecies (Table 1, SI Figures S26–S27). The best fits for the kidney spectra obtained from mice fed control and SeNP diets included roughly equivalent fractions of SeMet (21–22%), CysSe^−^ (28–35%, representing deprotonated SeCys) and CuSe (25–28%, representing a metal(II)-selenide metabolite) model spectra, with minor contributions from selenite (8–9%) and elemental Se (8–11%). In contrast, the best fits to testis model spectra for these groups included a greater proportion of CuSe (40–41%), a moderate fraction of selenocystine (27–28%, CysSeSeCys, representing a diselenide species), and minor contributions from SeMet (10–12%), CysSe^−^ (9–11%) and selenite (6–8%) model spectra. Linear combination fits of whole blood spectra from mice fed control and SeNP diets were largely comparable, containing similar proportions of selenite (3–4%), SeMet (18–20%), CysSe^−^ (11–13%) and CuSe (48–56%), although the contributions from the Se(0) model spectrum differed most substantially, with 10% present in the bet fit for blood from mice in the SeNP group versus 6% for the control group. The greater fit fraction of Se(0) in the whole blood from mice fed SeNPs may reflect the presence of elevated elemental Se in the bloodstream. Likewise, the best fits to liver spectra from mice fed control and SeNP diets were broadly similar, containing selenite (3–4%), SeMet (24–26%), CysSe^−^ (20%) and CuSe (36–42%), albeit with differing minor fractions of Se(0) (modelled as GSSeSG), with the liver from the SeNP group reporting 11% compared to 5% in the best fit to the control group spectrum. This result further substantiates the possible increase in Se(0), perhaps present as STS metabolites or unmetabolised SeNPs, in the blood and liver of mice fed the SeNP diet.

The spectra obtained from the various tissues from mice fed SeN were similar to each other (highlighted in SI Figure S28), indicating uniform Se speciation in the kidney, blood, liver and testes. Linear combination fitting of these spectra (Table 1, SI Figure S29) revealed that the major component in the best fits was the reduced SeN (Se=C(NR_2_)_2_) model spectrum, in relatively consistent proportions of 60–74% for all tissues. In each case, the remainder of the fit contained some combination of CuSe, SeMet, CysSe^−^ (or CysSeSeCys for the testis) model spectra in quantities of 6–17%. Notably, the Se Kα_1_ HERFD-XAS analysis of the SeN-enriched dough feed indicated that both reduced and oxidised forms of SeN were present in the diet in an approximately 1:1 ratio (SI Figures S12–S13, Table S2). The fits for tissue spectra from animals fed the SeN diet were revised (Table 2, SI Figure S30) using the reduced and oxidised SeN models and corresponding control tissue spectra to represent the ‘typical’ mixture of selenospecies condensed into one model. In these fits, control tissue spectra comprised 23–39% of the total fit, alongside 56–70% contributed by the reduced SeN model spectrum. Small fractions (8–11%) of the oxidised SeN (R–Se–Se–R) model spectrum were present in the best fits to the blood and testis. These fits gave improved or equivalent residual values when compared to previous fits using only chemical models, except for the testis spectra. The absence of the oxidised SeN model in the best fits to the kidney and liver tissue spectra suggests that any oxidised SeN present in the feed was effectively reduced in the bloodstream (only 8% fit as oxidised SeN). Subsequently, SeN was transported to the kidney and liver, where residual oxidised SeN may have been fully reduced, however, our data suggests that SeN was not further metabolised in these organs. Given the poorer residual value for the testis fit, and the presence of another diselenide model spectrum in the best fit to the control testis tissue, the proportion of the oxidised SeN model (11%) may not be reliable. However, it is possible that some oxidised SeN from the bloodstream accumulated in this tissue. Notwithstanding this uncertainty, these results undoubtedly demonstrate that mice fed the SeN diet accumulated primarily reduced SeN in the bloodstream and in all analysed tissues.

**Table 2.** Percent Se species in tissues from mice fed a diet containing 5 14;mg Se/kg as SeN.*^a^* Fit fractions are estimated by a linear combination of model compound spectra.*^b^*

Tissue	Percentage (%) Se species			N_tot_*^c^*	Residual (×10^−3^)
	Control Tissue	SeN (R)	SeN (O)		
Kidney	29 (1)	70 (1)	–	0.99	0.76
Blood	23 (2)	67 (1)	8 (1)	0.99	0.91
Liver	39 (1)	56 (1)	–	0.95	1.07
Testis	30 (4)	56 (2)	11 (2)	0.97	1.73

*^a^* Refit using the control tissue spectrum and SeN models. *^b^* Values in parentheses are the estimated standard deviations derived from the diagonal elements of the covariance matrix and are a measure of precision. *^c^* N_tot_ is the sum of the fractions.

Additionally, in all the above mouse tissue spectra (Figure 5), we note the presence of a small pre-edge feature at ∼12,655 14;eV, with the greatest intensity in the kidney tissue spectra. Though not included as a model in the linear combination fits, we recognise that this feature likely represents the previously reported SeCys radical species (Cys-Se^•^) which is generated from the exposure of the sample to the X-ray beam [83,109].

## 4. Discussion

### 4.1. Bioavailability, stability and metabolism of SeNPs

In a 2014 study, Loeschner and colleagues reported elevated fractions of elemental Se in the livers and kidneys of rats orally administered BSA-stabilised SeNPs, with a mean diameter of 19 14;nm (and range of 10–80 14;nm), at a dose of 0.5 14;mg Se/kg per day [110]. This investigation indicated that daily dietary administration of 0.5 14;mg Se/kg as either SeNPs or selenite resulted in equivalent elevation of selenoprotein P (SelP) in blood plasma. This finding, combined with the comparable effects of SeNPs and selenite on GPx upregulation reported in previous studies in mice [44], led to the conclusion that both forms of Se display equal bioavailability and influence on selenoprotein activity, with upregulation primarily associated with Se dose rather than chemical form [110]. However, earlier studies of supranutritional supplementation with SeNPs of variable sizes suggests that their influence on selenoprotein expression and total Se status is more irregular and complex. Importantly, adverse effects including weight loss [41–43,111,112], liver degeneration and pathological changes [42,43,111,112], and oxidative stress marked by increased malondialdehyde concentrations indicative of lipid peroxidation [42,43], have been observed in several toxicological studies of SeNPs in laboratory animals under various treatment conditions. These reoccurring results and additional toxicological effects observed in isolated studies, highlight the importance of further characterisation of the absorption, metabolism and long-term toxicity of SeNPs [86].

An important uncertainty from several existing studies of SeNPs arises because researchers have often neglected to adequately characterise the size and morphology of the nanoparticles. Alternatively, nanoparticles are often administered as non-homogenous mixtures with a broad range of particle sizes. This is a significant oversight since nanoparticle size is a critical property which influences bioavailability and bioactivity [63]. In a 2012 study by Zhang *et al*., smaller BSA-coated SeNPs (40 14;nm) were found to exhibit superior thermostability compared to larger SeNPs (80 14;nm), the latter of which were observed to aggregate into larger particles and form nanorods when heated to 90 °C [59]. These heat-treated SeNPs displayed reduced bioactivity in Se-deficient mice compared to unheated 80 14;nm SeNPs which exhibited proportionally greater effects on increasing hepatic Se content and glutathione S-transferase (GST) activity [59].

Earlier works demonstrated that supranutritional supplementation with BSA-coated SeNPs, with an average diameter of 36 14;nm, increased Se concentrations in the livers of mice administered with variable doses up to 5 14;mg Se/kg by weight [42]. Subsequently, the bioactivity of these smaller nanoparticles was found to have a greater impact on blood Se content and enhanced hepatic GST activity compared to analogous treatments with larger SeNPs (∼90 14;nm) following oral administration at various supranutritional doses between 0.5–2 14;mg Se/kg by weight for 7 days [45]. Beyond investigations in animal models, a recent study of SeNPs by Cheng *et al.* demonstrated that the Se content in rhizosphere soil and uptake into *Brassica chinensis* shoots was significantly elevated following exposure to 30 14;nm SeNPs, with greater elevations in Se compared to treatments with larger nanoparticles at 50, 80 and 100 14;nm [113]. Collectively, these findings emphasise that smaller nanoparticles are generally more readily absorbed and accessible for bioconversion in both plant and mammalian systems.

Notably, the results from the rodent studies described above appear to contrast with the findings from the present investigation, where dietary supplementation with SeNPs had minimal impact on the concentration and distribution of Se within the liver and other organs. Wang *et al.* reported elevations of Se in mouse liver up to ∼10-fold following a short-term (7 days) toxicity study with a daily dose of 5 14;mg Se/kg [42]. In the present study, mice consuming ∼3 14;g per day of the 10 14;mg Se/kg SeNP feed were ingesting ∼1.0–1.5 14;mg Se/kg by weight daily over the course of three weeks. In the 2007 study by Wang *et al.*, Se-deficient mice orally administered 1 14;mg Se/kg per day for 7 days showed substantial elevations in Se concentration in the blood (∼6-fold), liver (∼9-fold) and kidneys (∼2-fold), relative to the control group [42]. While rodents in the present study were not Se-deficient, Se Kα_1_ HERFD-XAS spectra of whole blood and liver from mice fed SeNPs suggested potentially greater proportions of elemental Se in these tissues compared to the control, and XFM revealed a statistically significant elevation in Se concentration in glomeruli ROIs. However, these results indicate lower bioavailability compared to the 36 and 90 14;nm BSA-coated SeNPs in previous works [42,45].

Recent reviews of SeNPs for nutritional supplementation and Se biofortification in plants suggest that the lower surface area to unit volume of SeNPs reduces physiochemical interactions and results in a gradual release of Se into the organism compared to other selenospecies [114,115]. This could partially explain the absence of significant elevations in Se across whole tissues; however, it is also possible that the rate of excretion largely outbalances the uptake of Se in nanoparticulate form. Whilst we did not obtain urinary or faecal data in the present study, a recent study of dietary Se supplementation in mice has shown that the gut microbiome is a major source of Se consumption, with the chemical form of dietary Se (inorganic, organic or nanoparticle Se) influencing the population and distribution of microorganisms [89]. Bacteria have a significantly larger selenoproteome than mammals, thus, the utilisation of Se by gut microbiota can limit the efficiency of Se uptake into tissues [89,116]. Specifically, the 2025 study by Mojadadi *et al.* reported that mice fed diets containing nanoparticulate Se (2.7 ± 0.3 14;mg Se/kg, SeNP sizes 40.1 ± 15.3 14;nm) and MeSeCys (4.7 ± 0.5 14;mg Se/kg) exhibited similarly significant increases in richness and α-diversity relative to the control diet [89]. Beyond the potential impacts on Se bioavailability, these microbiome changes induced by SeNP supplementation reflect important influences on gut health and general immunity.

Given the absence of urinary data in the current study, we are unable to evaluate the proportion of Se excreted during supranutritional supplementation with SeNPs. However, studies of mice fed both Se-adequate and Se-deficient diets after injection of ^82^Se-enriched selenite have demonstrated that exogenous ^82^Se was rapidly transformed into cellular GPx in the liver and kidneys, with excess Se found to be associated with selenosugars [117]. These selenosugars, including the major identified species *Se*-methylseleno-N-acetylgalactosamine (MeSeGalNAc) and *Se*-methylseleno-N-acetylglucosamine (MeSeGluNAc), are considered the primary urinary Se metabolites excreted by mammals consuming Se within the recommended and low-toxicity range [11,118,119]. Hence, we anticipate that excess bioavailable Se from the SeNP feed, administered at a sub-toxic dose, would be processed and excreted via a similar metabolic pathway. In addition, hyphenated chromatography and mass spectrometry studies have reported appreciable concentrations of low molecular weight selenosugars and high molecular weight ‘selenosugar-decorated proteins’ in the livers of rats and turkeys supplemented with variable doses of SeMet and selenite [107,108]. In lieu of Se Kα_1_ HERFD-XAS model spectra for selenosugars, we anticipate that contributions from these species in the liver and kidney may have been compensated largely by SeMet (R–Se–Me).

It is broadly accepted that the absorption and metabolism of orally administered nanoparticles occurs in the gastrointestinal tract (GIT), with transport facilitated via pores (∼20–800 14;µm) in the mucus lining of the small and large intestines [63,114,120,121]. Subsequent tissue penetration and cellular uptake of nanoparticles is purported to occur via a range of potential active transport routes involving endocytosis [114,122]. Hence, both the size and surface chemistry of nanoparticles can dictate the efficiency of absorption. However, these properties may be modified within the GIT, as the variable pH conditions can influence nanoparticle agglomeration [123], and interactions with gastric proteins and enzymes may lead to alterations to the nanoparticle surface chemistry changing their charge and agglomeration state [114,124].

Furthermore, some researchers have proposed that BSA-stabilised SeNPs may be dissolved and oxidised to inorganic oxoanions of Se within the GIT [110,125], given existing evidence for the microbial oxidation of elemental Se to selenite (Se(IV)) in soil slurries and bacterial cultures [126,127]. Additionally, recent work using capillary electrophoresis coupled with ICP-MS demonstrated that SeNPs coated with polyvinyl alcohol were unstable in solutions of 50% human plasma, partially degrading into selenite over 5 days of incubation (total selenite fraction of 5%) [128]. However, our XFM data indicates absence of the characteristic accumulation and colocalisation of Se and Cu within the renal cortex which has been observed in rodents administered with selenite and STS compounds [81,82]. Instead, the distributions of Se and other endogenous elements (Zn, Cu, Fe) within the kidneys, liver and testes of animals fed SeNP diets were analogous to the control group, and the composition of selenospecies within these tissues and whole blood remained broadly unchanged. This suggests that oxidation of BSA-coated SeNPs to Se(IV) *in vivo* is negligible or occurs at a rate too slow to elicit the previously observed physiological effects in the rodent renal cortex.

Importantly, in the present study, SeNPs were incorporated into rodent feed, thus, physical modification of the nanoparticles may have occurred prior to entering the organism. Beyond the difference in the mean size of SeNPs, this method of administration of SeNPs to mice differs from the studies discussed above where rodents were given SeNPs via oral gavage. The present work represents one of few studies incorporating SeNPs into animal feed [89,125,129–132], with previous studies primarily focussing on the general health of poultry and livestock fed Se-enriched diets. Here we report the only current example of a synchrotron-based study of the *in situ* distribution and speciation of Se in mice following supplementation with SeNPs. Uniform distribution of Se in the dough form pellets was confirmed by ICP-MS and Se Kα_1_ HERFD-XAS verified that the feed was primarily composed of elemental Se, however, we were unable to adequately characterise the morphology and size of the SeNPs after incorporation into the feed. Thus, it is possible that aggregation and agglomeration of SeNPs may have occurred within the feed, reducing the proportion of bioavailable elemental Se (particles ≤ 100 14;nm in size) in the diet. This highlights a potential caveat in the design nutritional supplements containing SeNPs and an important consideration for the administration of SeNPs for biomedical or agricultural applications, such as supplemented livestock feed.

### 4.2. *In vivo* reduction and bioaccumulation of SeN

At present, it remains unclear whether SeN can serve as a Se source for utilisation in the biosynthesis of selenoproteins. Rather it appears that the unique chemistry of SeN may afford it the ability to directly scavenge free radicals as a small molecule or via binding to proteins such as hemoglobin and myoglobin, providing protection against iron autooxidation [64,65]. In a manner identical to its sulfur analogue, the reduced monomer of SeN exhibits chalcogen-centred tautomerism and a pH dependent equilibrium between selenol (H–Se–C(NR_2_)_2_) and selenone (also known as a selone or selenoketone, Se=C(NR_2_)_2_) tautomer forms [65,70]. Selenols are strong nucleophiles with low redox potentials and typically lower pK_a_ values (∼5) than thiols (∼8), permitting greater ionisation at neutral pH and subsequently greater reducing capabilities [133]. Correspondingly, the significant antioxidant capabilities of selenoproteins are largely attributed to the high reactivity of the selenol group present at the active site [133,134]. However, there is currently insufficient evidence to suggest that the selenol form of SeN is biologically relevant and present *in vivo*. The pK_a_ of ergothioneine (ET) reported in literature is 10.8 [135], hence, at physiological pH, ET exists primarily as the thione form and has a high redox potential, which makes it substantially more resistant to autooxidation compared to other thiols [72,136]. Proton NMR spectra of synthetic SeN measured in buffered solutions between pH 6 and 13, has been used to determine a pK_a_ for SeN of 10.1 [88]. Therefore, SeN would exist primarily as the selenone form under physiologically relevant conditions. Additionally, computational studies of selone/selenol tautomerisation have demonstrated that the imidazole selone form is the most stable isomer, according to theoretical calculations simulated in the gas phase and in solvents, using both second-order perturbation theory and range-separated density functional theory models [137]. This is supported by the current Se Kα_1_ HERFD-XAS data for animal tissues from the SeN dietary group, as fits for these tissue spectra include large fractions of the reduced selenone model spectrum (with low residuals) and the selenol model does not contribute to the best fit.

In a 2020 study by Miyata *et al.*, supplementation with SeN (in a diet containing 0.3 14;mg Se/kg) was found to ameliorate hepatocellular injury and hepatic steatosis in a mouse model of non-alcoholic fatty liver disease (NAFLD) [138]. Analysis of hepatic gene expression levels revealed that the mRNA levels of oxidative stress-related genes encoding for heme oxygenase 1 and GSTs were decreased in mice fed SeN. Similarly, although hepatic Se levels were increased, SeN supplementation did not affect the mRNA levels of selenoproteins including SelP, GPx1, GPx2, GxP4 and TrxR1 [138]. These results indicate that the attenuation of oxidative stress conditions associated with NAFLD was not achieved via the upregulation of selenoproteins, but rather via an alternative mechanism which is yet to be elucidated. Thus, whilst SeN has been shown to offer protection against oxidative damage associated with various diseases, its bioactivity starkly contrasts to SeNPs which have been shown to enhance the expression of GPxs and other proteins involved in the oxidative stress response.

In 2013, Yamashita and colleagues demonstrated that human embryonic kidney cells transiently overexpressing the ET transporter organic cation/carnitine transporter 1 (OCTN1) could efficiently uptake SeN when administered as the oxidised dimer form (R–Se–Se–R), leading researchers to postulate that this transporter is essential for SeN activity *in vivo* [70,139]. In this study, SeN treatment in OCTN1-transfected cells and zebrafish was found to effectively reduce MeHg accumulation and levels of associated reactive oxygen species, providing protection against toxicity [139]. Subsequent *in vitro* studies have demonstrated that SeN can be reduced by glutathione (GSH) and/or other reductants [88], and that reduced SeN acts as suitable substrate for OCTN1 (alternatively described as the ergothioneine transporter, ETT) whereas the stable dimer form is not directly transported [140]. The *in vivo* mechanism for SeN reduction and bioactivity is yet to be established, however, researchers have proposed that oxidised SeN may be reduced in the digestive tract and absorbed via the epithelial cells in the small intestine which produce the OCTN1 transporter [76]. Additionally, synthetic SeN shows high stability under treatment with hydrogen peroxide and the seleninic acid intermediate generated in this reaction can be rapidly converted back to the selenone following treatment with GSH under physiological conditions, suggesting a stable cycle for the quenching of peroxide radicals [88].

Our XFM and Se Kα_1_ HERFD-XAS data support findings from the 2020 study by Miyata *et al.* where mice fed SeN diets showed significantly elevated total Se in the liver and blood (1.7 and 1.9 times greater than the control diet, respectively), which was characterised by liquid chromatography coupled with ICP-MS as unmetabolised SeN (selenone, reduced form) [138]. Given the greater dose of SeN in our dough form feed (5 14;mg Se/kg), we observed a much larger fold increase in Se concentration in the liver (∼7.5-fold), compared to the mice fed 0.3 14;mg Se/kg in the 2020 study [138]. While not directly proportional to the difference in dose, this substantial elevation may indicate a relatively slow rate of excretion for SeN, which aligns with the trophic bioaccumulation highlighted in studies of populations consuming seafood diets rich in SeN [66,141]. Beyond this, the present data indicates that this accumulation of Se as reduced SeN also occurs within the mouse kidney and testes, with increases of ∼3.2-fold and ∼1.9-fold total Se, respectively, suggesting a ubiquitous distribution of SeN throughout the organism. Across the analysed tissues, XFM maps showed no significant perturbations in the distributions of endogenous elements (Zn, Cu, Fe), providing further confidence in the non-toxic nature of this organic selenospecies.

Importantly, the Se Kα_1_ HERFD-XAS spectra of our SeN-enriched dough form feed indicated that SeN in the diet was present as both oxidised diselenide (R–Se–Se–R) and the reduced selenone (Se=C(NR_2_)_2_) forms. Hence, the observed significant proportions of the reduced SeN model in the linear combination fits to mouse tissue and whole blood spectra substantiate the hypothesis that oxidised SeN is reduced *in vivo* and remains in the reduced form under physiological conditions. However, in the absence of immunohistochemical data we cannot verify the reported negligible effects of SeN supplementation on the expression and activities of selenoproteins. Beyond this, our study did not assess the composition of urinary and faecal samples from mice fed SeN. Given the typically low concentrations of Se in urine (20–60 μg Se/L), notwithstanding its advantages over conventional XAS, identification of urinary metabolites via Se Kα_1_ HERFD-XAS analysis would remain challenging. Despite these low parts-per-billion concentrations, previous high resolution chromatography and mass spectrometry studies have detected both selenosugars and methylated SeN (*Se*-methylselenoneine, MeSeN) in the non-preconcentrated urine of non-supplemented humans [74]. This finding highlights the natural presence of SeN in mammalian diets and contributes new insight into the composition of selenospecies in urine, many of which remain unknown [74].

The relevance of dietary SeN and its potential health benefits is anticipated to increase given recent identification of high levels of SeN in the popular edible porcini mushroom (*Boletus edulis*) [142]. While the present study investigated only the mouse kidney, liver and testes tissues, previous work has demonstrated that SeN is likely capable of crossing the blood–brain barrier, however, its transfer rate and permeability coefficient was significantly below those of selenite and MeSeCys in the *in vitro* model system [143]. Notably, no metabolism of SeN was identified in the primary porcine brain capillary endothelial cells [143], again corroborating current and previous findings which suggest that reduced SeN persists in the body, with only minor proportions of methylated SeN metabolites detected in the urine, blood plasma, kidney and liver [74,75]. SeN was also found to comprise 78–88% of the total Se content in the brains of giant petrels [67]. Hence, it has been postulated that SeN could play an important role in the protection of the nervous system through antioxidant activity and metal-detoxification, making it a potential therapeutic for neurodegenerative disease [144].

## 5. Conclusion

Through the application of synchrotron-based X-ray techniques, this study has demonstrated key distinctions between the distribution and speciation of Se in the tissues and whole blood of mice fed potentially therapeutic selenocompounds. Statistical analysis of XFM data collected for replicate mouse kidneys suggested that dietary supplementation with SeNPs (10 14;mg Se/kg in feed) increased total Se content in the blood and Se Kα_1_ HERFD-XAS spectra of a corresponding whole blood sample indicated a potentially greater fraction of elemental Se in these animals. Notably, the absence of significant increases in Se content in the whole kidney and liver tissues contradicts several previous studies of supranutritional supplementation with SeNPs in rodent models. This disparity further highlights the complex and sensitive physico-chemical properties of BSA-stabilised SeNPs and the potential modifications to the size, morphology and surface chemistry of nanoparticles which may occur within food products and *in vivo*, impacting the bioavailability of Se when administered in this dietary form. In contrast, statistically significant elevations in Se were identified in all tissues analysed (kidney, liver and testis) from mice supplemented with SeN (5 14;mg Se/kg in feed) and Se Kα_1_ HERFD-XAS spectra of these tissues and whole blood revealed that Se was consistently accumulated as the reduced monomer form of SeN throughout the organism. These findings corroborate previous reports of negligible metabolism of SeN and trophic bioaccumulation and transfer between marine organisms and humans consuming high seafood diets. Whilst the animals in the present feeding trial showed no signs of adverse health effects from supplementation with either SeNPs or SeN, further studies are required to comprehensively evaluate and quantify the nutritional benefits and therapeutic effects of these selenocompounds. This is particularly necessary for SeN, as the mechanisms behind its bioactivities have not yet been fully elucidated.

## Supplementary Material

SeNPsSeN_Mice_Supplementary_Redox_Rep_Revised_Jan2025_clean.docx

## Data Availability

The data supporting this article have been included as part of the Supplementary Information. Additional data and materials are available from the corresponding authors upon reasonable request.
