# Correction

**DOI:** 10.1080/13510002.2026.2648349

**Published:** 2026-03-24

**Article title:** XFM and HERFD-XAS studies of selenium in tissues and whole blood from mice supplemented with potentially therapeutic selenocompounds

**Authors:** Baker, A. T., Ortiz-Cerda, T., Xie, K., Hunter, J., Dragutinovic, I., Morris, Vogt, L. I., George, G. N., Sokaras, D., Howard, D. L., Witting, P. K. & Harris, H. H.

**Journal:** *Redox Report*

**Bibliometrics:** Volume 31, Number 1, pages 1-26

**DOI:** 10.1080/13510002.2026.2626151

This article was first published without Graphical Abstract and wrong Author Credit for author Jonathan Morris:

The article has been republished with the added Graphical Abstract and correct Author Credit.

