# Supplementary material for: XFM and HERFD-XAS studies of selenium in tissues and whole blood from mice supplemented with potentially therapeutic selenocompounds
Source: Redox Rep. 2026 Feb 16;31(1):2626151. doi: 10.1080/13510002.2026.2626151 (PMC12912245; doi:10.1080/13510002.2026.2626151)
Supplement: SeNPsSeN_Mice_Supplementary_Redox_Rep_Revised_Jan2025_clean.docx [file YRER_A_2626151_SM3664.docx]

**XFM and HERFD-XAS Studies of Selenium in Tissues and Whole Blood from Mice Supplemented with Potentially Therapeutic Selenocompounds**

Ani T. Baker,^a^ Tamara Ortiz-Cerda,^b,c^ Kangzhe Xie,^c^ Jordan Hunter,^c^ Iliya Dragutinovic,^d^ Jonathan C. Morris,^d^ Linda I. Vogt,^e^ Graham N. George,^e^ Dimosthenis Sokaras,^f^ Daryl L. Howard,^g^ Paul K. Witting,^c^ Hugh H. Harris^a*^

^a^ Discipline of Chemistry, The University of Adelaide, North Terrace, Adelaide, SA 5005, Australia

E-mail: [hugh.harris@adelaide.edu.au](mailto:hugh.harris@adelaide.edu.au); Tel: +61-08-83135060

^b^ Departamento de Citología e Histología Normal y Patológica, Facultad de medicina, Universidad de Sevilla, Seville, Spain

^c^ Redox Biology Group, Charles Perkins Centre, School of Medical Sciences, Faculty of Medicine and Health, The University of Sydney, Sydney, Australia

^d^ School of Chemistry, Faculty of Science, Kensington, UNSW Sydney, NSW, Australia

^e^ Department of Geological Sciences, University of Saskatchewan, 114 Science Place, Saskatoon, SK S7N 5E2, Canada

^f^ Stanford Synchrotron Radiation Lightsource, SLAC National Accelerator Laboratory, 2575 Sand Hill Road, Menlo Park, CA 94025, USA

^g^ Australian Synchrotron, ANSTO, 800 Blackburn Road, Clayton, VIC 3168, Australia

**Supplementary Information**

**Synthesis of selenoneine (SeN)**

**General experimental**

The full reaction scheme for the synthesis of SeN is provided in **Figure S1**. Schematics for the syntheses of each intermediate compound are provided in **Figures S2-S5** and accompanying ^1^H NMR data is shown in **Figures S6-S9**. Unless otherwise stated all reactions were performed in flame dried glassware under an atmosphere of high purity argon using dry solvents. Reagents and solvents were purchased from commercial sources and used without further purification unless stated. In those cases, reagents and solvents used in reactions were purified according to well established procedures [1]. In particular, tetrahydrofuran (THF) was freshly distilled from sodium and benzophenone under an inert atmosphere of argon. *N,N*-Dimethylformamide (DMF) was dried sequentially over three batches of 4 Ǻ molecular sieves (3 × 24 h), before finally being stored over a fourth batch of 4 Ǻ molecular sieves, under argon. To remove residual *N,N*-dimethylamine from DMF, the atmosphere was placed under vacuum (~0.1 mm/Hg) for at least 30 min prior to use. Methanol was distilled from magnesium and stored over 3 Ǻ molecular sieves under argon. Dichloromethane and triethylamine were distilled from calcium hydride prior to use. Reaction temperatures refer to external bath temperature. ^1^H NMR and ^13^C NMR were recorded on a Bruker Avance III 400 (400 MHz) or Bruker Avance III 600 (600 MHz), with data acquired and processed using TopSpin 4.4 software. Chemical shifts are expressed in parts per million (ppm) on the δ scale. Chemical shifts in (a) CDCl_3_ were referenced relative to CHCl_3_ (7.26 ppm) for ^1^H NMR and CDCl_3_ (77.16 ppm) for ^13^C NMR and (b) CD_3_OD were referenced relative to CH_3_OH (3.31 ppm) for ^1^H NMR and CD_3_OD (49.00 ppm) for ^13^C NMR. *t*-Butyllithium in pentanes was purchased from Sigma Aldrich and titrated using menthol and 2,2’-bipyridyl in THF as described by Eastham [2]. α-(Bromomethyl) styrene was prepared via previously reported method [3].

**Figure S1.** Reaction scheme for the stepwise synthesis of selenoneine (SeN). Reaction conditions are (a) α-(Bromomethyl) styrene, K_2_CO_3_, NaI, MeCN, 85 °C (b) Se, DMF, 80 °C, 82% over 2 steps (c) *t*-BuLi, THF, -78 °C, 30 min, 60% (d) TFA, DCM, rt (e) CH_2_O_(aq.)_, NaCNBH_3_, MeOH, rt, 52% over 2 steps (f) ClCO_2_Et, NET_3_, DCM, rt (g) MeI, THF, rt (h) LiOH.H_2_O, H_2_O, rt, 15% over 3 steps.

**Synthesis of compounds**

**Methyl (*S*)-3-(1,3-bis(2-phenylallyl)-2-selenoxo-2,3-dihydro-1*H*-imidazol-4-yl)-2-((*tert*-butoxycarbonyl)amino)propanoate (1)**

**Figure S2.** Reaction scheme for synthesis of compound **1** from compound **2** via intermediate compound **3** over three steps.

Potassium carbonate (15.8 g, 114 mmol), sodium iodide (2.10 g, 14.0 mmol), and α-(bromomethyl) styrene (22.5 g, 114 mmol) were added sequentially to a suspension of Boc-His-OMe (**2**) (12.3 g, 45.7 mmol) in dry acetonitrile. The reaction mixture was heated at 85 °C for 20 h before being filtered through a plug of celite, eluting with dichloromethane. The solvent was removed under reduced pressure and the crude intermediate (**3**) was redissolved in dry *N,N*-dimethylformamide (140 mL). To this mixture was added potassium carbonate (7.0 g, 50.3 mmol) and elemental selenium (4.0 g, 50.3 mmol). The reaction mixture was heated at 80 °C for 22 h before being cooled to room temperature, filtered through a plug of celite eluting with dichloromethane, and the solvents were removed under a stream of N_2_ with care taken to avoid exposure to volatile selenium compounds during evaporation. The crude material was purified via flash column chromatography on deactivated silica gel, eluting with 40% ethyl acetate/petroleum spirits, to afford the title compound **1** as a yellow oil (22.0 g, 83% over 2 steps) with analytical data matching that reported in the literature [4]: ^1^H NMR (400 MHz; CDCl_3_) δ 7.51 – 7.43 (m, 4H), 7.38 – 7.27 (m, 6H), 6.62 (s, 1H), 5.64 (s, 1H), 5.14 – 5.40 (m, 6H), 4.99 (d, *J* = 7.5 Hz, 1H), 4.45 (q, *J* = 6.1 Hz, 1H), 4.37 (t, *J* = 1.7 Hz, 1H), 3.58 (s, 3H), 3.00 (dd, *J* = 15.6, 5.8 Hz, 1H), 2.85 (dd, *J* = 15.6, 5.8 Hz, 1H), 1.40 (s, 9H).

**Dimethyl 3,3'-(diselanediylbis(1*H*-imidazole-2,4-diyl))bis(2-((*tert*-butoxycarbonyl) amino)propanoate) (4)**

**Figure S3.** Reaction scheme for synthesis of compound **4** from compound **1** in one step.

*t*-Butyllithium (1.7 M in pentane, 35 mL, 60.3 mmol) was added dropwise to a solution of protected selenohistidine **1** (7.0 g, 12.1 mmol) in freshly distilled THF (84 mL) at -78 °C. The reaction mixture was stirred at -78 °C for 30 min before being quenched with dry methanol (14 mL) and warmed to room temperature. Saturated aqueous sodium bicarbonate solution was added and the mixture was extracted with dichloromethane (×4). The combined organic extracts were washed with brine (×1) and dried (Na_2_SO_4_). The solvent was removed under reduced pressure and the crude material was purified via flash column chromatography on silica gel, eluting with ethyl acetate, to afford the title compound **4** as a foam (2.5 g, 60%) with analytical data matching that reported in the literature [4]: ^1^H NMR (400 MHz; CD_3_OD) δ 7.01 (s, 2H), 4.49 – 4.32 (m, 2H), 3.72 (s, 6H), 3.15 – 3.07 (m, 2H), 2.94 (dd, *J* = 15, 9.4 Hz, 2H), 1.40 (s, 18H).

**Dimethyl 3,3'-(diselanediylbis(1*H*-imidazole-2,4-diyl))bis(2-(dimethylamino)propanoate) (5)**

**Figure S4.** Reaction scheme for synthesis of compound **5** from compound **4** over two steps.

Trifluoroacetic acid (60 mL) was added dropwise over 30 min to a solution of Boc-selenohistidine compound **4** (8.0 g, 11.5 mmol) in dichloromethane (240 mL) at room temperature. The reaction mixture was stirred at room temperature for 2 h before the solvent was removed under a stream of N_2_. The residue was repeatedly co-evaporated with diethyl ether to remove residual trifluoroacetic acid and the resulting solid was redissolved in methanol (480 mL). To this solution was successively added aqueous formaldehyde solution (37%, 9.4 mL) and sodium cyanoborohydride (5.8 g, 92.3 mmol) at room temperature. The reaction mixture was stirred at room temperature for 20 h before the solvent was removed in vacuo. The residue was diluted with saturated aqueous sodium bicarbonate solution and extracted with dichloromethane (×10). The combined organic extracts were washed with brine (×1), dried (Na_2_SO_4_) and the solvent was removed under reduced pressure. The crude material was purified via flash column chromatography on silica gel, eluting with 15% ethanol, 40% heptane and 45 % ethyl acetate, to afford the title compound **5** (3.29 g, 52% over 2 steps) with analytical data matching that reported in the literature [4]: ^1^H NMR (400 MHz; CD_3_OD) δ 6.78 (s, 2H), 3.66 (s, 6H), 3.52 (dd, *J* = 8.5, 6.9 Hz, 2H), 2.94 (dd, *J* = 14.8, 8.5 Hz, 2H), 2.86 (ddd, *J* = 14.9, 6.9, 0.7 Hz, 2H), 2.35 (s, 12H)

**(±)-Selenoneine**

**Figure S5.** Reaction scheme for synthesis of compound (±)-selenoneine from compound **5**.

Triethylamine (2.2 mL, 15.5 mmol) and ethyl chloroformate (1.5 mL, 15.5 mmol) were added sequentially to a solution of diselenide **5** (1.9 g 3.44 mmol) in dichloromethane at 0 °C. The reaction mixture was stirred at 0 °C for 90 min before being diluted with dichloromethane, washed with water (×1), brine (×1) and dried (Na_2_SO_4_). The solvent was removed under reduced pressure and the resulting crude residue (2.6 g) was taking up in dry THF (24 mL). Iodomethane (0.770 mL, 12.4 mmol) was added dropwise to the above solution at room temperature. The reaction mixture was stirred for 18 h at room temperature and the resulting precipitate was collected via vacuum filtration. Crude precipitate **6** (2.5 g) was dissolved in water (350 mL) and lithium hydroxide monohydrate (2.0 g, 47.7 mmol) was added in one portion at room temperature. The reaction mixture was stirred for 2 h at which point it was deemed to be complete by ^1^H NMR spectroscopy. The pH of the solution was adjusted to 2-3 with 2M hydrochloric acid and the solvent volume was reduced to ~30 mL under a stream of N_2_. The solution was incubated at 4 °C for 3 days then filtered to remove the insoluble material. The volume of the solution was further reduced via partial lyophilisation and the resulting solid was collected via filtration to give selenoneine hydrochloride as an orange powder (275 mg, 15% over 3 steps) with analytical data matching that reported in the literature [4]: ^1^H NMR (400 MHz; D_2_O) δ 7.46 (s, 2H), 4.00 (dd, *J* = 12, 3.9 Hz, 2H), 3.55 (dd, *J* = 14, 3.9 Hz, 2H), 3.41 (dd, *J* = 12, 1.6 Hz, 2H), 3.33 (s, 18H).

**References:**

1. Armarego, WL, Chai, CLL, *Purification of laboratory chemicals*. 6 edn, Oxford, United Kingdom: Butterworth-Heinemann, 2009.

2. Watson, SC, Eastham, JF. Colored indicators for simple direct titration of magnesium and lithium reagents, *J Organomet Chem,* 1967;**9**: 165-68. <https://doi.org/10.1016/S0022-328X(00)92418-5>

3. Dong, X, Xu, L-P, Yang, Y*, et al.* A palladium/Et3N·HI-catalyzed highly selective 7-endo alkyl-Heck-type reaction of epoxides and a DFT study on the mechanism, *Org Chem Front,* 2021;**8**: 6009-18. <https://doi.org/10.1039/D1QO00942G>

4. Abd El-Hack, ME, Ashour, EA, Baset, SA*, et al.* Effect of Dietary Supplementation of Organic Selenium Nanoparticles on Growth Performance and Carcass Traits of Broiler Chickens, *Biol Trace Elem Res,* 2024;**202**: 3760-66. <https://doi.org/10.1007/s12011-023-03948-x>

**^1^H NMR Data**


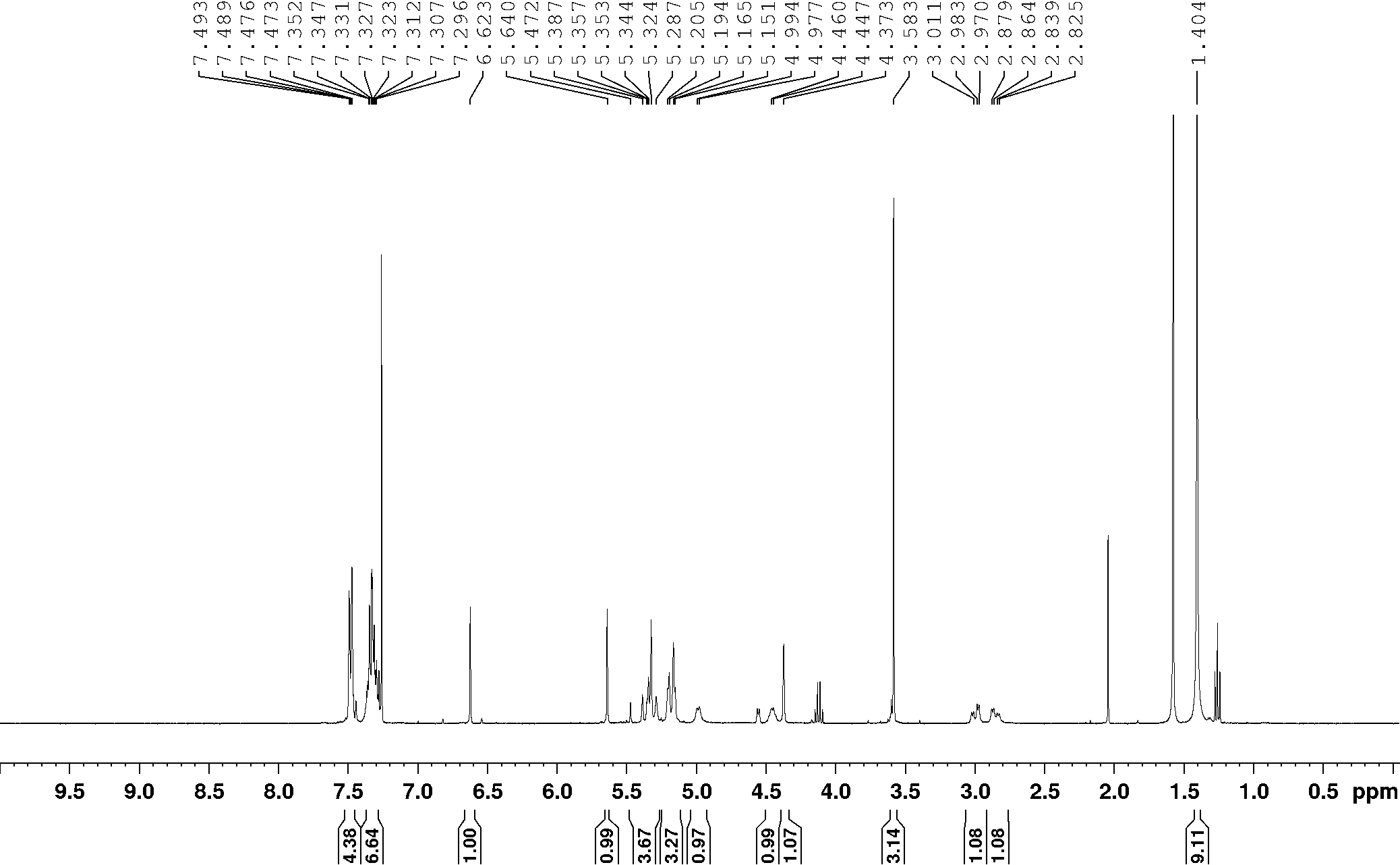


**Figure S6.** ^1^H NMR (400 MHz; CD_3_OD) spectrum for methyl (*S*)-3-(1,3-bis(2-phenylallyl)-2-selenoxo-2,3-dihydro-1*H*-imidazol-4-yl)-2-((*tert*-butoxycarbonyl)amino)propanoate (**1**).


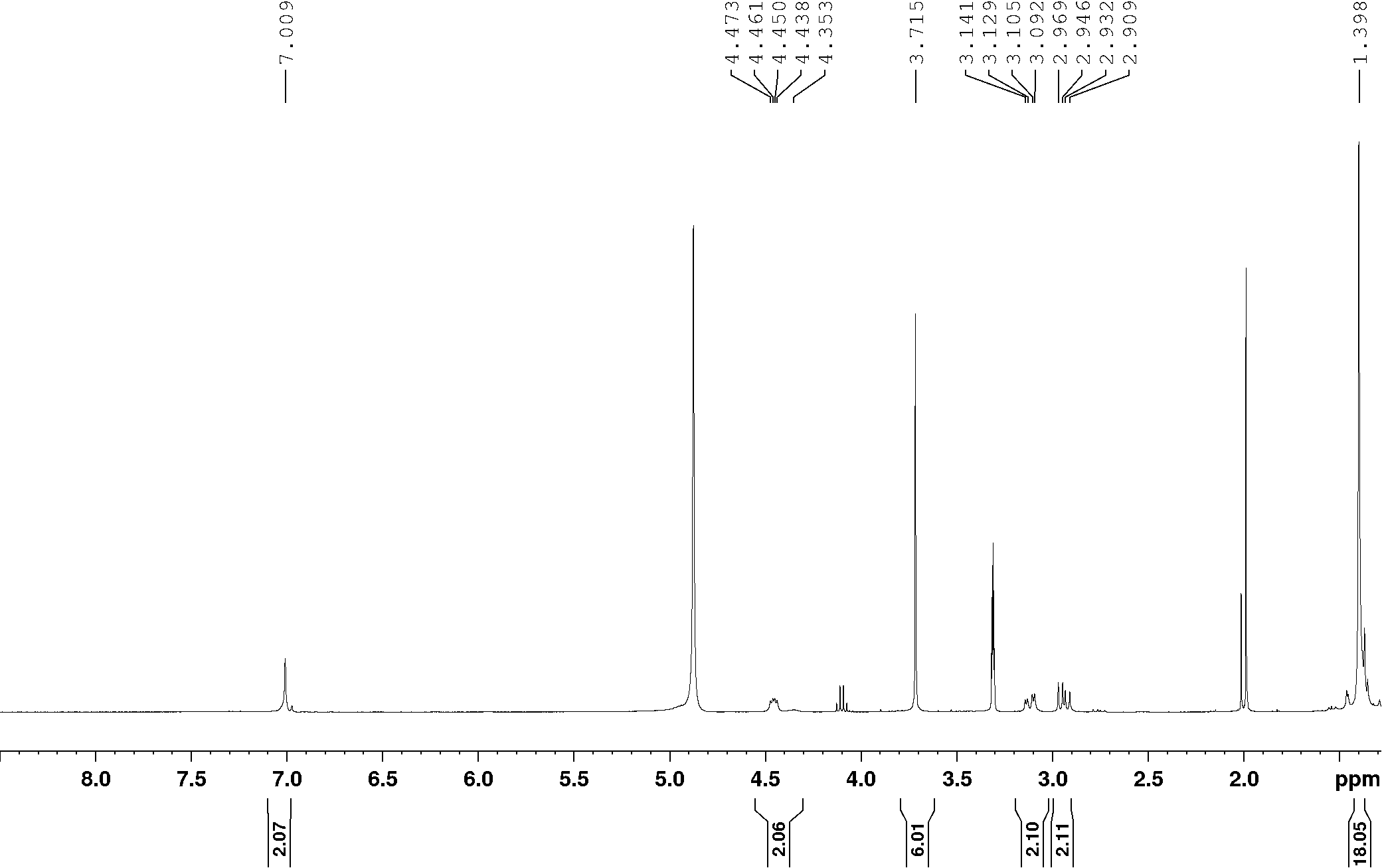


**Figure S7.** ^1^H NMR (400 MHz; CD_3_OD) spectrum for dimethyl 3,3'-(diselanediylbis(1H-imidazole-2,4-diyl))bis(2-((tert-butoxycarbonyl) amino)propanoate) (**4**).

**
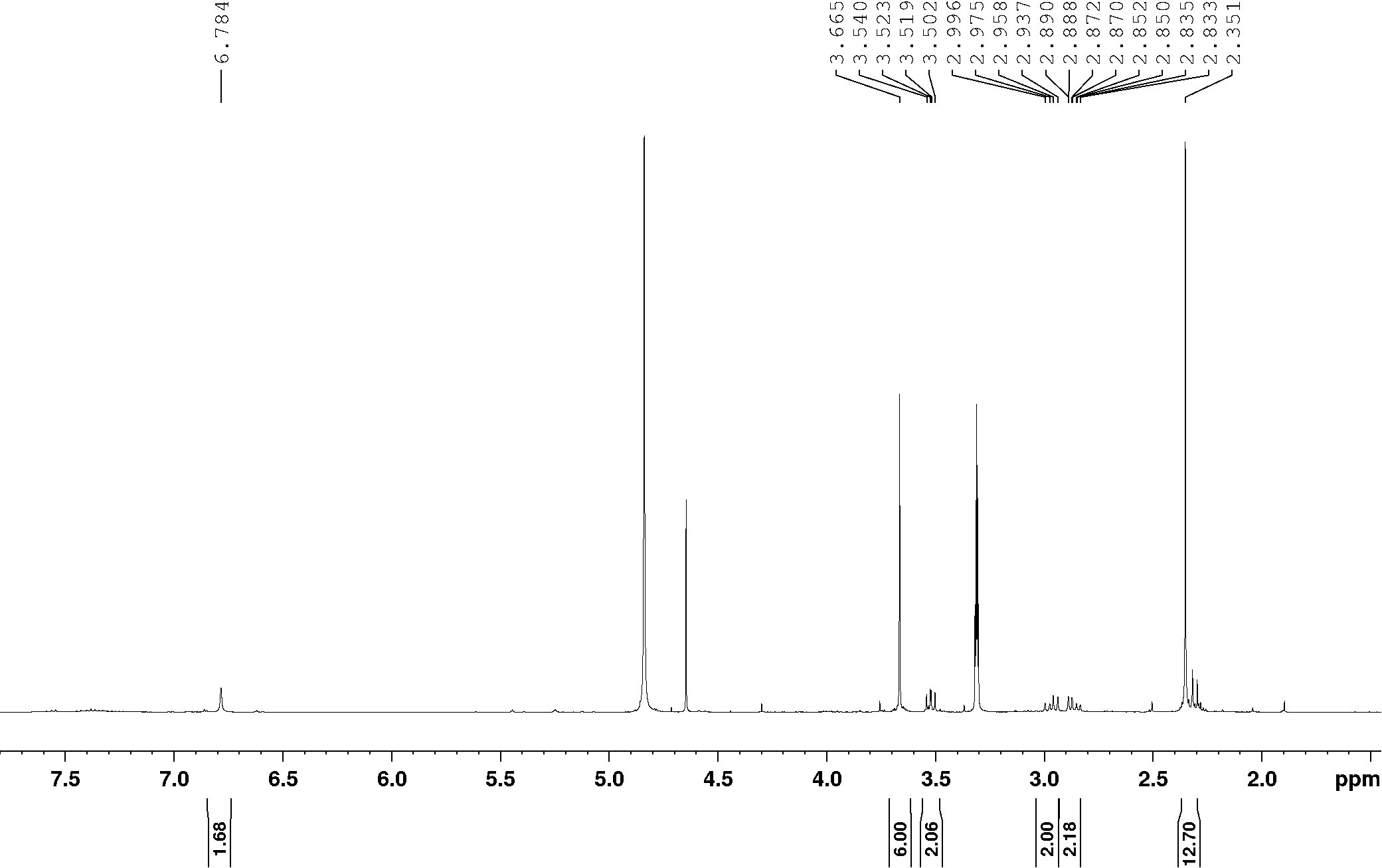
**

**Figure S8.** ^1^H NMR (400 MHz; CD_3_OD) spectrum for dimethyl 3,3'-(diselanediylbis(1H-imidazole-2,4-diyl))bis(2-(dimethylamino)propanoate) (**5**).

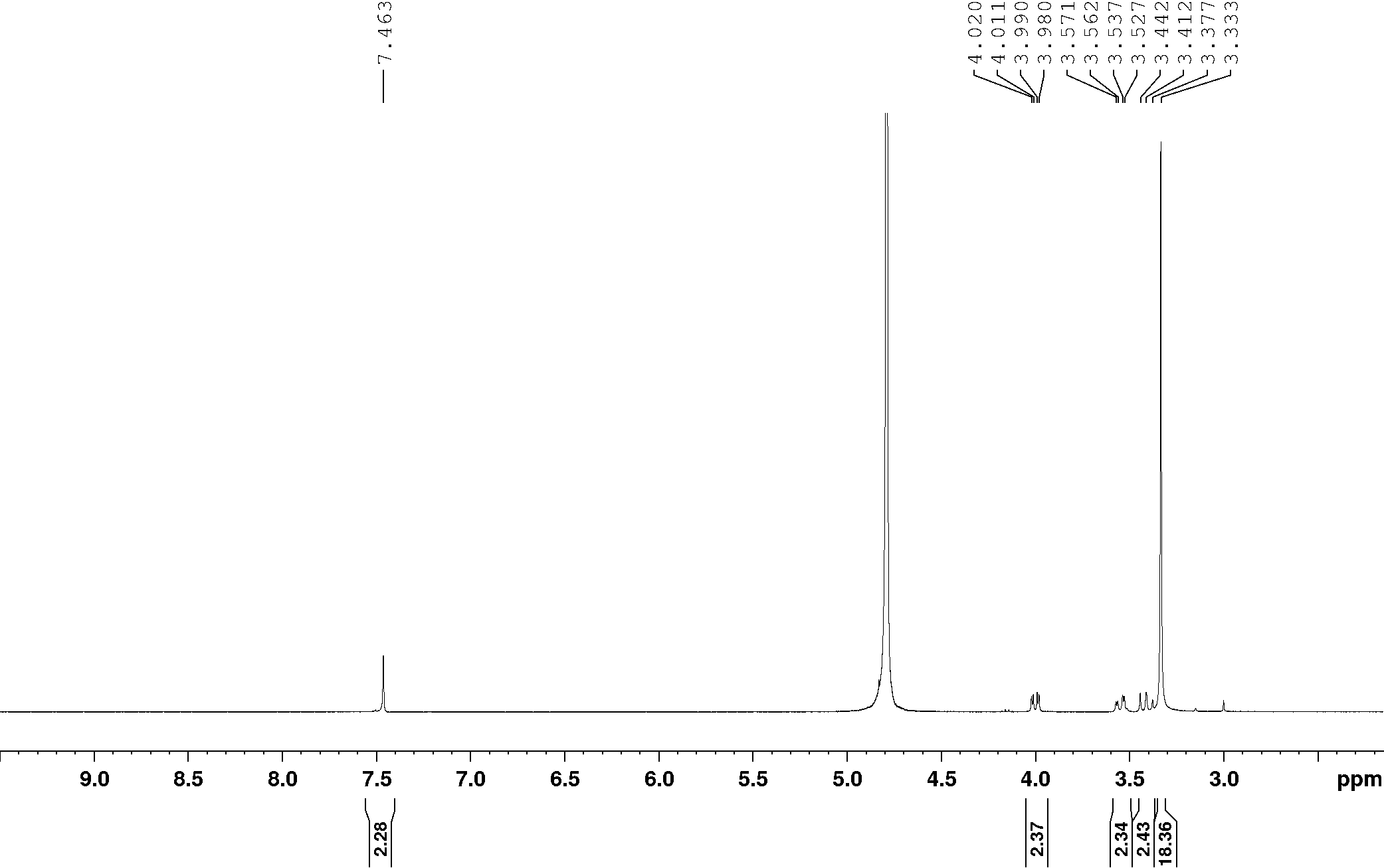


**Figure S9.** ^1^H NMR (400 MHz; CD_3_OD) spectrum for (±)-selenoneine hydrochloride.

**Characterisation of selenium nanoparticles (SeNPs)**


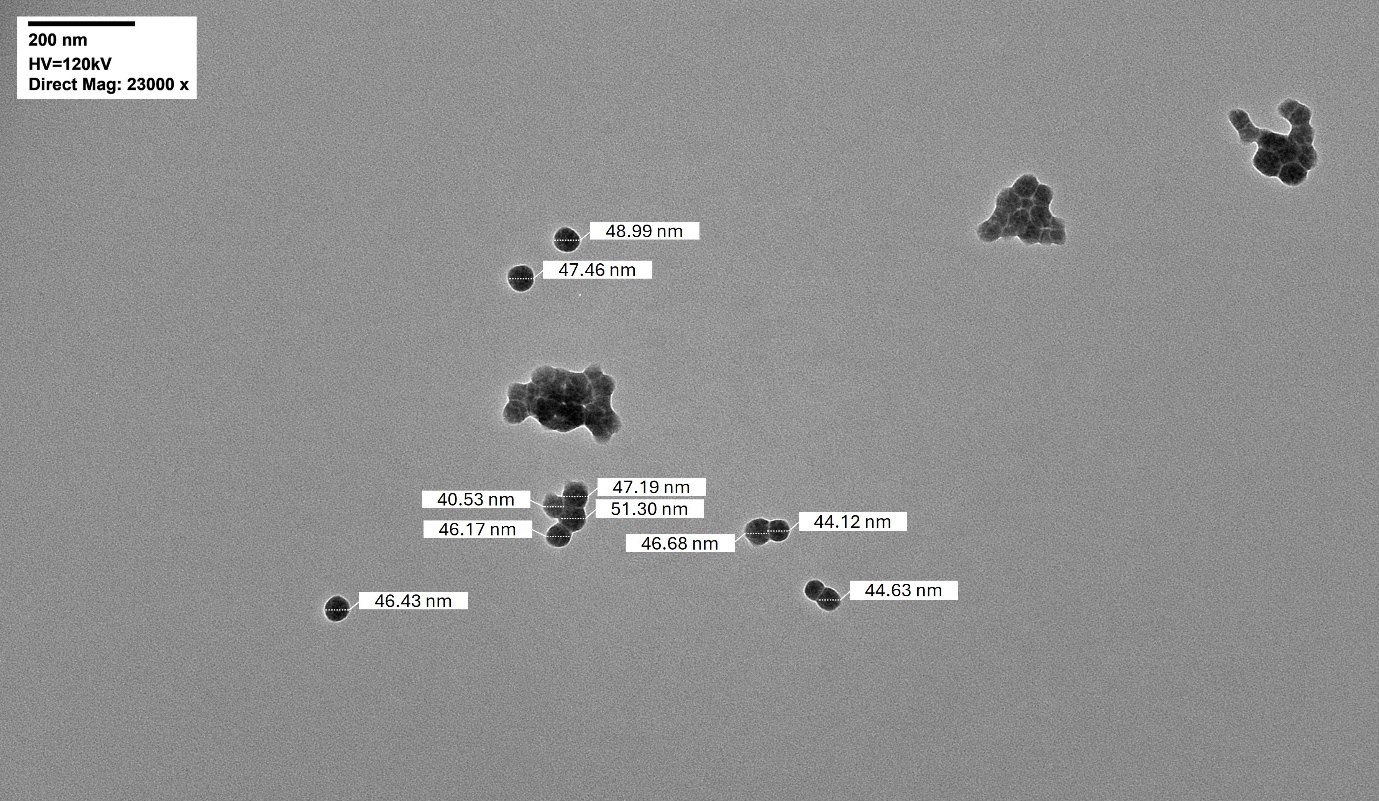


**Figure S10.** Transmission electron microscopy (TEM) image of selenium nanoparticles (SeNPs) coated with bovine serum albumin (BSA), synthesised using a 1:5 molar ratio of sodium selenite to ascorbic acid at 600 rpm. The diameter of SeNPs was determined to be 46.3 ± 0.9 nm (mean ± SEM) from *n =* 10 measurements with individual values shown in the TEM image. The scale bar (200 µm), accelerating voltage (120 kV) and direct magnification (23000X) are displayed in the top left corner.

**Verification of SeNP diameter**

The chemical synthesis of BSA-coated SeNPs was conducted following an existing procedure described in the literature. In the original study by Chung *et al.* [1], SeNPs produced under variable reaction conditions were extensively characterised. Utilising a 1:5 molar ratio of sodium selenite to ascorbic acid and agitating the mixture at 600 rpm was found to yield SeNPs with a spherical morphology and an average diameter of 42 nm, as determined by dynamic light scattering (DLS) measurements [1]. In the present work, these reaction conditions were applied to synthesise BSA-coated SeNPs for incorporation into the rodent feed. Prior to feed preparation, a sample of the SeNPs was analysed by TEM to verify that the nanoparticles were synthesised at the desired size (40-50 nm in diameter). Despite modifying the surface hydrophilicity of the carbon film TEM grid and sonicating the suspension of BSA-coated SeNPs prior to drop coating, the spherical nanoparticles were observed to aggregate into larger clusters on the carbon grid. In areas where isolated SeNPs were identified, the diameter was measured as 46.3 ± 0.9 nm from *n =* 10 representative individual nanoparticles shown in the TEM image (**Figure S10**). Hence, the morphology and average SeNPs diameter was consistent with the literature reports. While direct measurements were not taken from SeNPs presented in larger clusters, visual inspection suggested that the diameter of individual nanoparticles both dispersed and clustered were uniform.

Due to the small mean diameter of the SeNPs and their low abundance once incorporated into the rodent feed (at 10 mg Se/kg), the density of SeNPs was too low for successful characterisation of the SeNP-enriched dry powder or dough pellets by TEM and/or scanning electron microscopy. Therefore, the potential aggregation and agglomeration of SeNPs after incorporation into the feed could not be verified by these methods, however, the homogeneity of Se within the feed was assessed by ICP-MS for both diets.

**References:**

1. Chung, S, Zhou, R, Webster, TJ. Green Synthesized BSA-Coated Selenium Nanoparticles Inhibit Bacterial Growth While Promoting Mammalian Cell Growth, *Int J Nanomedicine,* 2020;**15**: 115-24. <https://doi.org/10.2147/ijn.S193886>

**Animal monitoring data**

**
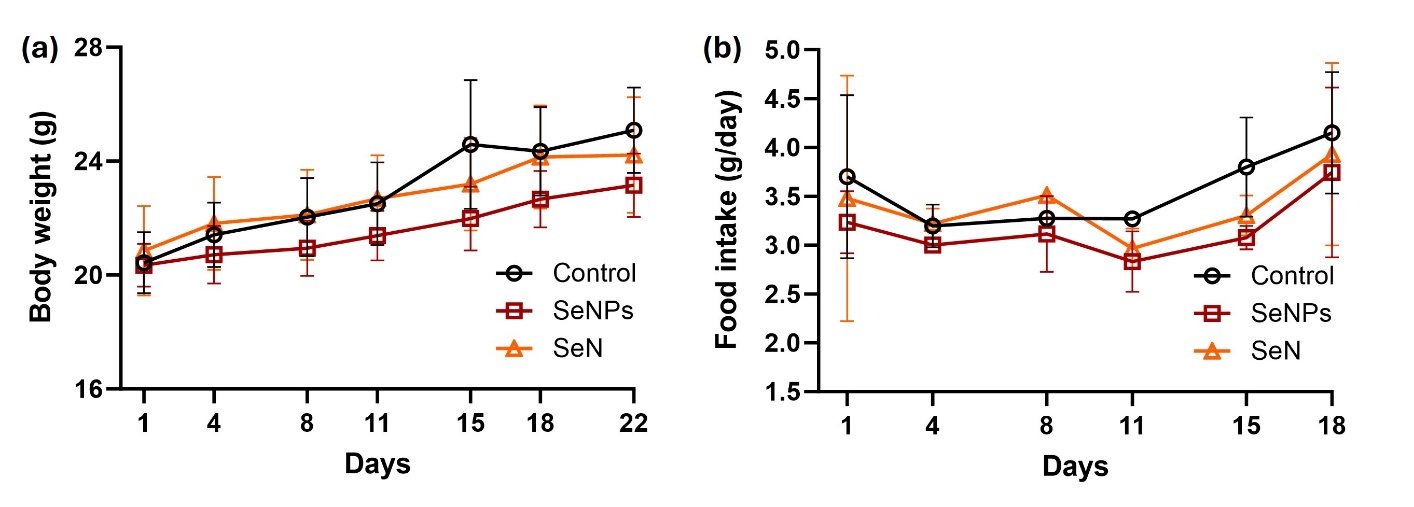
**

**Figure S11.** General monitoring data of mice during 3-week feeding trial, showing **(a)** body weights (g) measured for individual mice (*n =* 6), and **(b)** estimated intake of food (g/day) averaged across two cages (3 individuals, 2 cages per group) for each dietary group. Data points represent the mean from **(a)** 6 (mice) or **(b)** 2 (cages) measurements, with error bars showing the corresponding standard deviation. Note that error bars cannot be seen for several data points in **(b)** as the standard deviations fall below 0.1 g/day. Diets: Control containing ≈ 0.3 mg Se/kg (black trace, circles), 10 mg Se/kg as SeNPs (red trace, squares), and 5 mg Se/kg as SeN (orange trace, triangles).

**ICP-MS and HERFD-XAS analysis of rodent feed**

**Table S1.** Se concentration in rodent diets quantified by ICP-MS. Feed was prepared using a modified dough form diet with no added selenium (SF23-021, Specialty Feeds, WA, Australia) in large-scale batches of 2 kg. Samples were digested in 70% nitric acid (2 h, 60^o^C).

| **Diet*^a^*** | **Sample Form** | **Se (mg/kg)*^b^*** |
| --- | --- | --- |
| Control | Dry Powder | n.d.*^c^* |
|  | Dough Pellet | n.d.*^c^* |
| SeNPs | Dry Powder | 13.1 ± 1.2 |
|  | Dough Pellet | 13.0 ± 1.0 |
| SeN | Dry Powder | 8.06 ± 0.21 |
|  | Dough Pellet | 4.1 ± 0.4 |

*^a^* Intended Se concentrations for diets were; control ≈ 0.3 mg Se/kg (according to manufacturer specifications), SeNPs = 10 mg Se/kg, and SeN = 5 mg Se/kg. *^b^* Concentrations were calculated from *n =* 4 dry powder or *n =* 8 dough pellet samples (2 x half segments from 4 x pellets) and are presented as mean ± SEM. *^c^* ‘n.d.’ = below the limit of detection and indicates a concentration < 0.46 mg Se/kg.


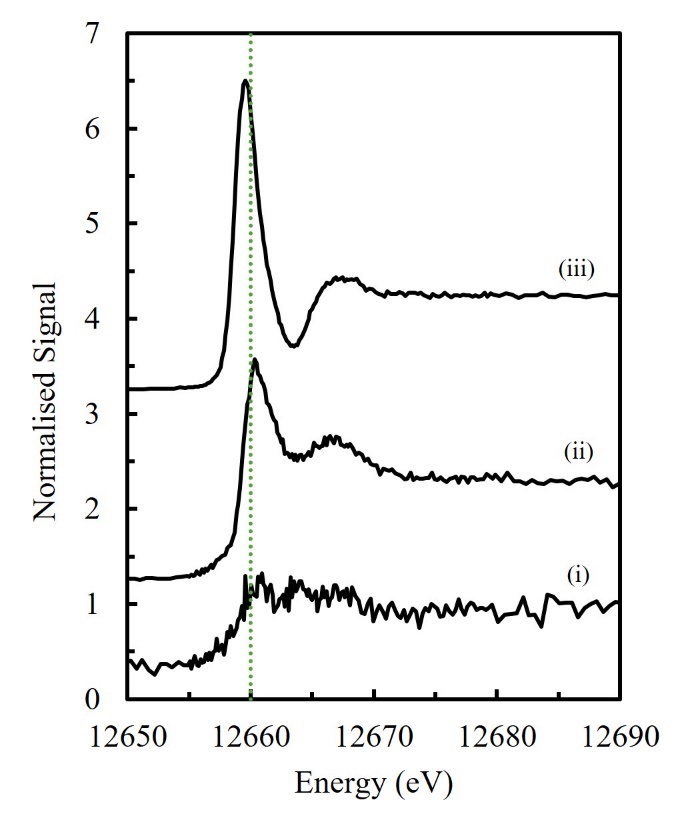


**Figure S12.** Se Kα_1_ HERFD-XAS XANES spectra of dough form pellets prepared for each dietary group; (i) control (unamended, ~0.3 mg Se/kg), (ii) 5 mg Se/kg as SeN, and (iii) 10 mg Se/kg as SeNPs. The green dotted line at 12,660.0 eV is included to emphasise differences in the spectral peak positions.

**Table S2.** Percent Se species in dough form diets containing 10 mg Se/kg as SeNPs or 5 mg Se/kg as SeN.*^a^* Fit fractions are estimated by a linear combination of model compound spectra.*^b^*

| **Diet** | **Percentage (%) Se species** | | | | | **N_tot_*^d^*** | **Residual (×10^−3^)** |
| --- | --- | --- | --- | --- | --- | --- | --- |
|  | **SeO_3_^2−^ *^c^*** | **SeNPs** | **CysSeH** | **SeN (R)** | **SeN (O)** |  |  |
| SeNPs | 3 (1) | 79 (1) | 12 (1) | – | – | 0.94 | 5.42 |
| SeN | – | – | 29 (1) | 41 (1) | 36 (1) | 1.05 | 2.76 |

*^a^* Dough form diets were prepared using SF23-021 feed supplied by Specialty Feeds (WA, Australia). *^b^* Values in parentheses are the estimated standard deviations derived from the diagonal elements of the covariance matrix and are a measure of precision. *^c^* Model spectrum was shifted by −0.25 eV to account for the shift between Kα_1_ HERFD-XAS fluorescence lines for selenite (oxidised Se) and selenomethionine (reduced Se). *^d^* N_tot_ is the sum of the fractions.


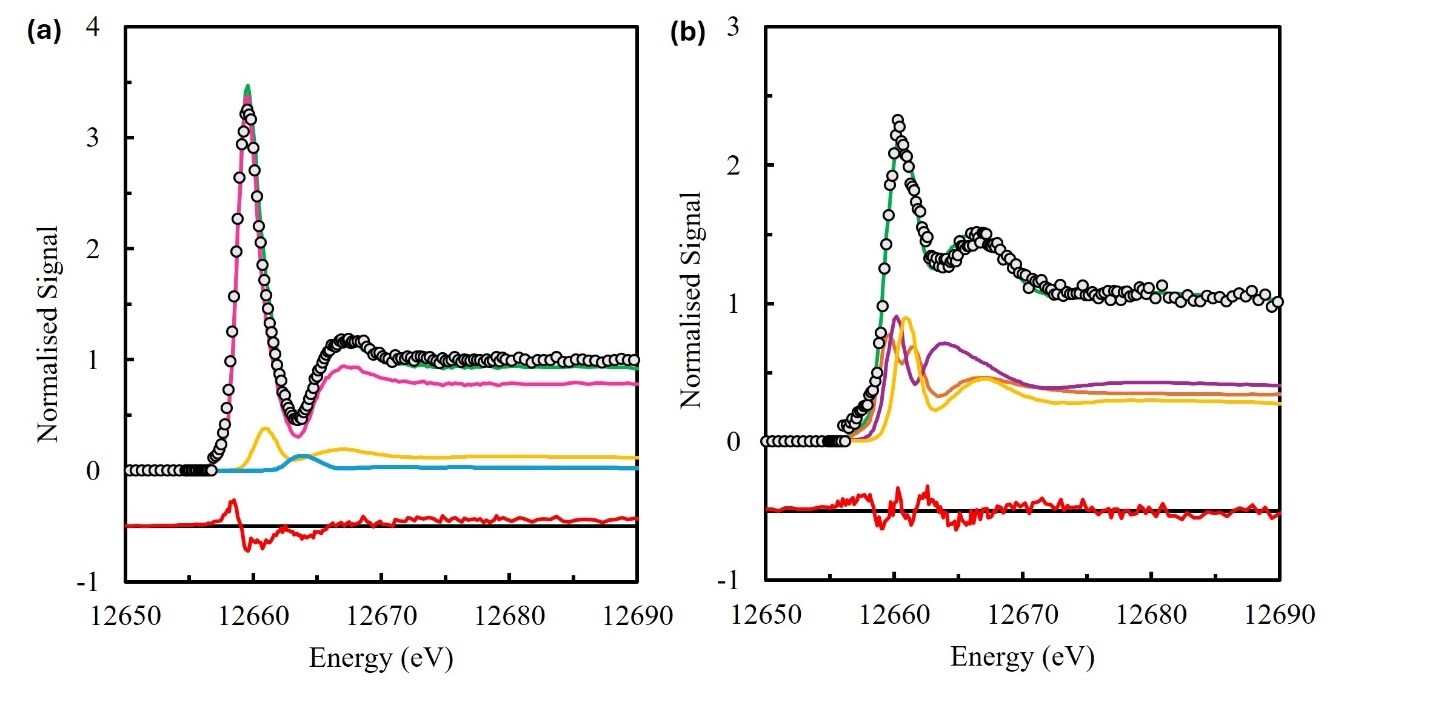


**Figure S13.** Linear combination analysis of Se Kα_1_ HERFD-XAS spectra of the rodent feed prepared with **(a)** 10 mg Se/kg as SeNPs, and **(b)** 5 mg Se/kg as SeN. The experimental data (points, grey) and the linear combination fit (green line) are displayed, along with components used in the best fit, scaled by their contributions to the fit: BSA-coated SeNPs, magenta line; CysSeH (R–Se–H), yellow line; selenite (pH 5.5), light blue line; SeN (oxidised, R–Se–Se–R), orange line; and SeN (reduced, (Se=C(NR_2_)_2_), purple line. The lower red trace shows the fit residual. Fit fractions for all components are provided in **Table S2**.

**Se distribution and speciation in rodent feed**

**ICP-MS data**

Se-enriched feeds were analysed by ICP-MS to verify the concentration of Se in each diet and evaluate the homogeneity across several dry powder (*n =* 4) and dough pellet samples (*n =* 8, 2 x half segments from 4 x pellets) (**Table S1**). The results indicated that both dry powder and dough pellet forms of the SeNP diet were marginally above the desired Se concentration of 10 mg Se/kg. In contrast, the concentration of Se in the SeN diet differed more distinctly between the dry powder and dough pellet forms, reporting above and below the target Se concentration of 5 mg Se/kg, respectively, albeit with greater consistency between replicate samples as indicated by the smaller uncertainties for this diet relative to the feed enriched with SeNPs. Control feed samples reported Se concentrations below the limit of detection, translating to < 0.46 mg Se/kg, which aligns with the manufacturer’s specifications for the diet. Additionally, the absence of significant matrix effects was demonstrated from good recovery (100-110%) of Se in control feed samples spiked with 100 µg Se/kg as sodium selenite.

Some amount of variability between feed samples and prepared dough pellets was anticipated given the challenge of incorporating such small quantities of SeNPs and SeN (< 40 mg) into a large final quantity of 2 kg. However, these results confirmed that the Se concentrations in the feeds were within an acceptable range which was approximately equivalent to the desired sub-toxic concentration. The ICP-MS data also provided good confidence that the distribution of Se was adequately homogeneous in both diets given the uncertainties of ≤ 1.2 mg Se/kg.

**HERFD-XAS data**

Crucially, alongside the evaluation of the Se concentration by ICP-MS, the preservation of chemical speciation of Se within the feed was verified by Se Kα_1_ HERFD-XAS analysis of dough pellet samples from each diet. The collected XANES spectra are provided in the Supplementary Information (**Figure S12)**. Linear combination fitting of the spectra (**Table S2**, **Figure S13**) indicated that Se-enriched dough pellets contained the desired selenospecies as the major fraction (77-79%). The best fit for the SeNP dough pellets included the BSA-coated SeNP model spectrum as the major component (79%), with minor contributions from the SeCys (12%) and selenite (3%) model spectra. Similarly, the best fit for the SeN dough pellet included SeN model compound spectra in both reduced (41%) and oxidised (36%) forms in an approximately 1:1 ratio, and a moderate fraction of SeCys (26%). The XANES spectrum collected for the control dough pellet demonstrated insufficient signal-to-noise for reliable fitting (**Figure S12**).

In each diet, the SeCys model spectrum (protonated, Se(II), CysSeH), fit the sample spectrum better than the SeMet model, and likely represents the mixture of selenoproteins natively present in the rodent feed. This fraction was proportionally greater in the SeN feed as the concentration of added Se (~5 mg Se/kg) was lower than that of the SeNP diet (~10 mg Se/kg). Notably, the presence of the selenite model spectrum was found to reduce the residual error for the fit to the SeNP feed, however, this fraction (3%) is minor which suggests that there was negligible degradation of the SeNPs during the preparation of the dough pellets.

**Statistical analysis of elemental distributions in tissues**


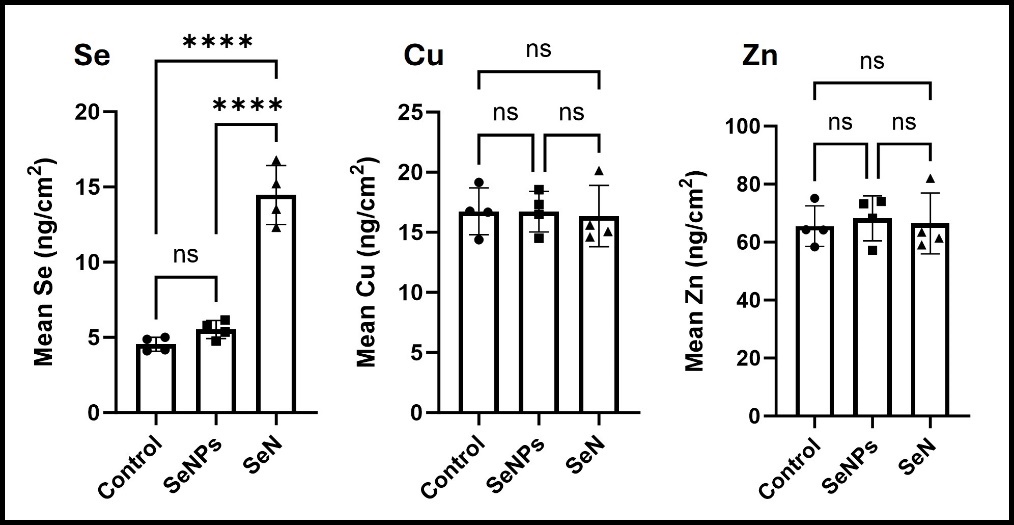


**Figure S14.** Comparisons of measured mean elemental concentrations of Se, Cu, and Zn in mouse kidneys (total area) from each dietary group. Data represents mean ± SD for *n =* 4 biological replicates (individual data points are provided). The level of statistical significance for each comparison is indicated. Significant = *P* < 0.05 as determined by an ordinary one-way ANOVA and Tukey multiple comparisons test, assuming normality and equal variance. Notation “ns” indicates no significance, and symbols *, **, ***, **** indicate *P* < 0.05 < 0.01 < 0.001 < 0.0001, respectively.

**Table S3.** Results from an ordinary one-way ANOVA to compare mean elemental concentrations (Se, Cu, Zn) in the total area of mouse kidney from each treatment group. Biological replicates: *n* = 4. F critical = 4.26, for significance level = 0.05.

| **Element** |  | **SS** | **DF** | **MS** | **F** | **P** |
| --- | --- | --- | --- | --- | --- | --- |
| Se | Between Groups | 239.4 | 2 | 119.7 | 81.78 | < 0.0001 (****) |
|  | Within Groups | 13.17 | 9 | 1.464 |  |  |
|  | Total | 252.6 | 11 |  |  |  |
|  |  |  |  |  |  |  |
| Cu | Between Groups | 0.3845 | 2 | 0.1922 | 0.04386 | 0.9573 (ns) |
|  | Within Groups | 39.45 | 9 | 4.383 |  |  |
|  | Total | 39.83 | 11 |  |  |  |
|  |  |  |  |  |  |  |
| Zn | Between Groups | 14.75 | 2 | 7.376 | 0.1006 | 0.9053 (ns) |
|  | Within Groups | 660.2 | 9 | 73.35 |  |  |
|  | Total | 674.9 | 11 |  |  |  |

**Table** **S4.** Results from a post-hoc Tukey’s multiple comparisons test of mean elemental concentrations (Se, Cu, Zn) in the total area of mouse kidney from each treatment group. Biological replicates: *n* = 4.

| **Element** | **Comparison** | **Mean Diff.** | **SE of Diff.** | **95% CI of Diff.** | | **P** |
| --- | --- | --- | --- | --- | --- | --- |
|  |  |  |  | **Lower Bound** | **Upper Bound** |  |
| Se | Control vs SeNPs | -0.9798 | 0.8555 | -3.368 | 1.409 | 0.5123 (ns) |
|  | Control vs SeN | -9.927 | 0.8555 | -12.32 | -7.538 | < 0.0001 (****) |
|  | SeNPs vs SeN | -8.947 | 0.8555 | -11.34 | -6.558 | < 0.0001 (****) |
|  |  |  |  |  |  |  |
| Cu | Control vs SeNPs | 0.02425 | 1.480 | -4.109 | 4.158 | 0.9999 (ns) |
|  | Control vs SeN | 0.3912 | 1.480 | -3.742 | 4.525 | 0.9624 (ns) |
|  | SeNPs vs SeN | 0.3670 | 1.480 | -3.766 | 4.500 | 0.9668 (ns) |
|  |  |  |  |  |  |  |
| Zn | Control vs SeNPs | -2.677 | 6.058 | -19.59 | 14.23 | 0.8991 (ns) |
|  | Control vs SeN | -0.9390 | 6.058 | -17.85 | 15.97 | 0.9869 (ns) |
|  | SeNPs vs SeN | 1.738 | 6.058 | -15.17 | 18.65 | 0.9559 (ns) |


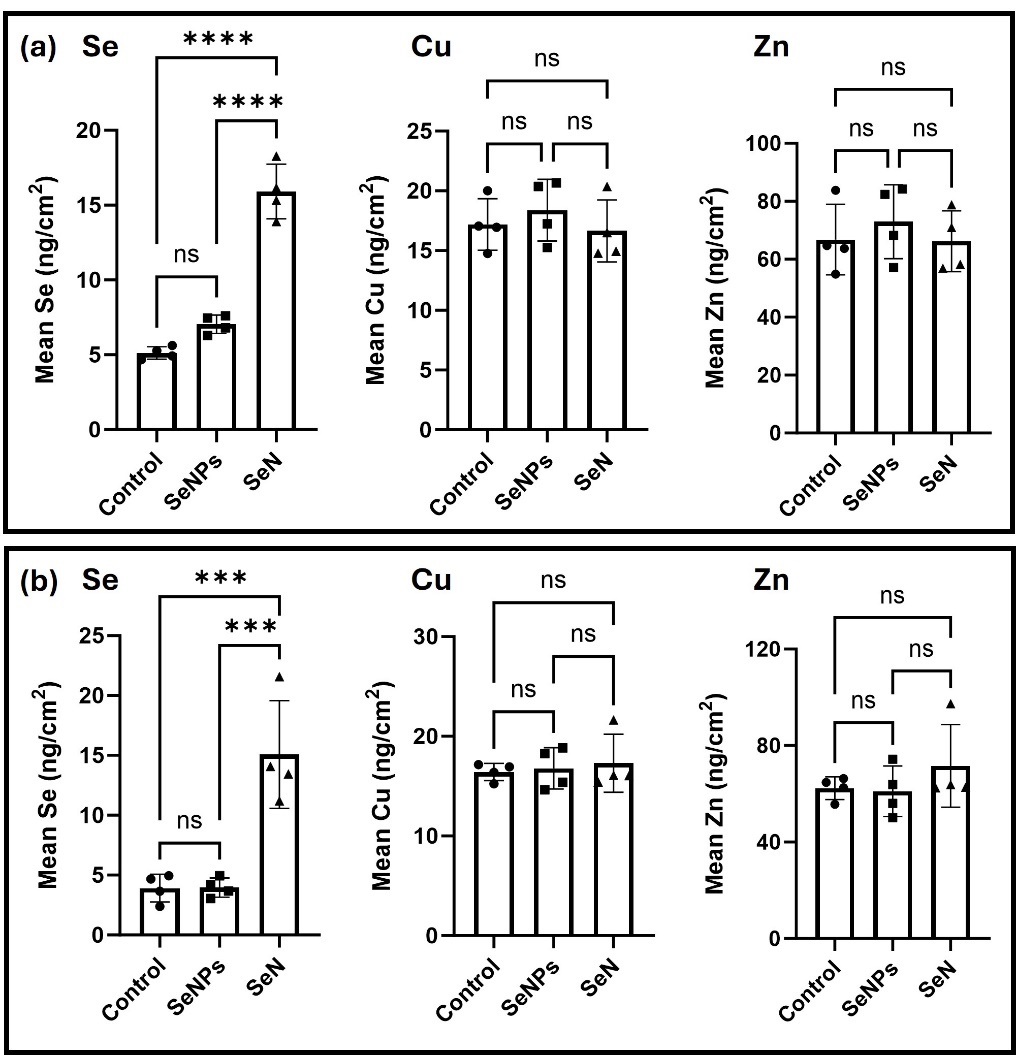


**Figure S15.** Comparisons of measured mean elemental concentrations of Se, Cu and Zn in **(a)** cortex and **(b)** medulla ROIs in mouse kidneys from each dietary group. Data represents mean ± SD for *n =* 4 biological replicates (individual data points are provided). The level of statistical significance for each comparison is indicated. Significant = *P* < 0.05 as determined by an ordinary one-way ANOVA and Tukey multiple comparisons test, assuming normality and equal variance. Notation “ns” indicates no significance, and symbols *, **, ***, **** indicate *P* < 0.05 < 0.01 < 0. 001 < 0.0001, respectively.

**Table S5.** Results from an ordinary one-way ANOVA to compare mean elemental concentrations (Se, Cu, Zn) in cortex ROIs in mouse kidney from each dietary group. Biological replicates: *n* = 4. F critical = 4.26, for significance level = 0.05.

| **Element** |  | **SS** | **DF** | **MS** | **F** | **P** |
| --- | --- | --- | --- | --- | --- | --- |
| Se | Between Groups | 264.9 | 2 | 132.4 | 102.3 | < 0.0001 (****) |
|  | Within Groups | 11.65 | 9 | 1.294 |  |  |
|  | Total | 276.5 | 11 |  |  |  |
|  |  |  |  |  |  |  |
| Cu | Between Groups | 6.321 | 2 | 3.161 | 0.5241 | 0.6091 (ns) |
|  | Within Groups | 54.27 | 9 | 6.030 |  |  |
|  | Total | 60.59 | 11 |  |  |  |
|  |  |  |  |  |  |  |
| Zn | Between Groups | 112.8 | 2 | 56.41 | 0.4020 | 0.6804 |
|  | Within Groups | 1263 | 9 | 140.3 |  |  |
|  | Total | 1376 | 11 |  |  |  |

**Table S6 .** Results from a post-hoc Tukey’s multiple comparisons test of mean elemental concentrations (Se, Cu, Zn) in cortex ROIs in mouse kidney from each dietary group. Biological replicates: *n* = 4.

| **Element** | **Comparison** | **Mean Diff.** | **SE of Diff.** | **95% CI of Diff.** | | **P** |
| --- | --- | --- | --- | --- | --- | --- |
|  |  |  |  | **Lower Bound** | **Upper Bound** |  |
| Se | Control vs SeNPs | -1.916 | 0.8045 | -4.162 | 0.3300 | 0.0945 (ns) |
|  | Control vs SeN | -10.79 | 0.8045 | -13.03 | -8.539 | < 0.0001 (****) |
|  | SeNPs vs SeN | -8.870 | 0.8045 | -11.12 | -6.623 | < 0.0001 (****) |
|  |  |  |  |  |  |  |
| Cu | Control vs SeNPs | -1.198 | 1.736 | -6.046 | 3.651 | 0.7752 (ns) |
|  | Control vs SeN | 0.5393 | 1.736 | -4.309 | 5.387 | 0.9485 (ns) |
|  | SeNPs vs SeN | 1.737 | 1.736 | -3.111 | 6.585 | 0.5950 (ns) |
|  |  |  |  |  |  |  |
| Zn | Control vs SeNPs | -6.206 | 8.376 | -29.59 | 17.18 | 0.7464 (ns) |
|  | Control vs SeN | 0.5607 | 8.376 | -22.82 | 23.95 | 0.9975 (ns) |
|  | SeNPs vs SeN | 6.767 | 8.376 | -16.62 | 30.15 | 0.7078 (ns) |

**Table S7.** Results from an ordinary one-way ANOVA to compare mean elemental concentrations (Se, Cu, Zn) in medulla ROIs in mouse kidney from each dietary group. Biological replicates: *n* = 4. F critical = 4.26, for significance level = 0.05.

| **Element** |  | **SS** | **DF** | **MS** | **F** | **P** |
| --- | --- | --- | --- | --- | --- | --- |
| Se | Between Groups | 330.6 | 2 | 165.3 | 22.21 | 0.0003 (***) |
|  | Within Groups | 66.97 | 9 | 7.441 |  |  |
|  | Total | 397.5 | 11 |  |  |  |
|  |  |  |  |  |  |  |
| Cu | Between Groups | 1.559 | 2 | 0.7797 | 0.1729 | 0.8440 (ns) |
|  | Within Groups | 40.60 | 9 | 4.511 |  |  |
|  | Total | 42.16 | 11 |  |  |  |
|  |  |  |  |  |  |  |
| Zn | Between Groups | 262.6 | 2 | 131.3 | 0.9223 | 0.4322 (ns) |
|  | Within Groups | 1281 | 9 | 142.4 |  |  |
|  | Total | 1544 | 11 |  |  |  |

**Table S8 .** Results from a post-hoc Tukey’s multiple comparisons test of mean elemental concentrations (Se, Cu, Zn) in medulla ROIs in mouse kidney from each dietary group. Biological replicates: *n* = 4.

| **Element** | **Comparison** | **Mean Diff.** | **SE of Diff.** | **95% CI of Diff.** | | **P** |
| --- | --- | --- | --- | --- | --- | --- |
|  |  |  |  | **Lower Bound** | **Upper Bound** |  |
| Se | Control vs SeNPs | -0.05350 | 1.929 | -5.439 | 5.332 | 0.9996 (ns) |
|  | Control vs SeN | -11.16 | 1.929 | -16.55 | -5.775 | 0.0007 (***) |
|  | SeNPs vs SeN | -11.11 | 1.929 | -16.49 | -5.722 | 0.0007 (***) |
|  |  |  |  |  |  |  |
| Cu | Control vs SeNPs | -0.3598 | 1.502 | -4.553 | 3.833 | 0.9690 (ns) |
|  | Control vs SeN | -0.8783 | 1.502 | -5.071 | 3.315 | 0.8315 (ns) |
|  | SeNPs vs SeN | -0.5185 | 1.502 | -4.712 | 3.675 | 0.9369 (ns) |
|  |  |  |  |  |  |  |
| Zn | Control vs SeNPs | 1.224 | 8.437 | -22.33 | 24.78 | 0.9885 (ns) |
|  | Control vs SeN | -9.254 | 8.437 | -32.81 | 14.30 | 0.5394 (ns) |
|  | SeNPs vs SeN | -10.48 | 8.437 | -34.03 | 13.08 | 0.4600 (ns) |


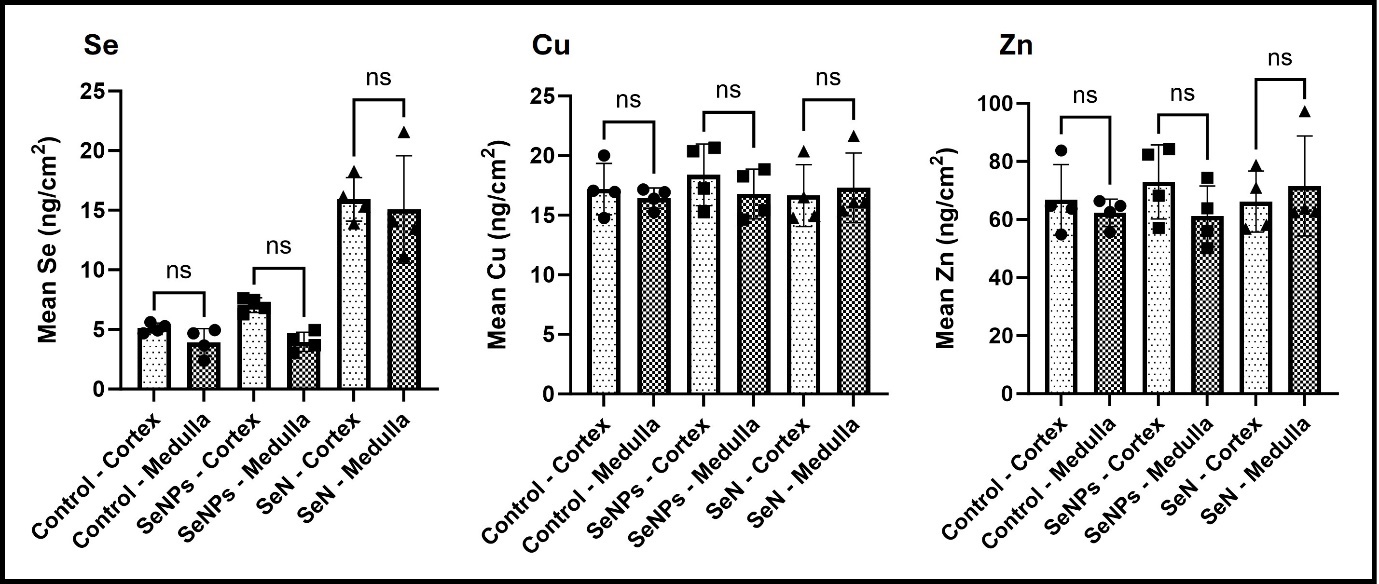


**Figure S16.** Selected pairwise comparisons between measured mean elemental concentrations of Se, Cu, and Zn in cortex and medulla ROIs in mouse kidneys from each dietary group. Data represents mean ± SD for *n =* 4 biological replicates (individual data points are provided). Significant = *P* < 0.05 as determined by an ordinary one-way ANOVA and post-hoc pairwise comparisons with Sidak’s correction, assuming normality and equal variance. Notation “ns” indicates no significance, and symbols *, **, ***, **** indicate *P* < 0.05 < 0.01 < 0.001 < 0.0001, respectively.

**Table S9.** Results from an ordinary one-way ANOVA to compare mean elemental concentrations (Se, Cu, Zn) between cortex and medulla ROIs in mouse kidney from each dietary group. Biological replicates: *n =* 4. F critical = 2.77, for significance level = 0.05.

| **Element** |  | **SS** | **DF** | **MS** | **F** | **P** |
| --- | --- | --- | --- | --- | --- | --- |
| Se | Between Groups | 613.0 | 5 | 122.6 | 28.07 | < 0.0001 (****) |
|  | Within Groups | 78.61 | 18 | 4.367 |  |  |
|  | Total | 691.6 | 23 |  |  |  |
|  |  |  |  |  |  |  |
| Cu | Between Groups | 9.846 | 5 | 1.969 | 0.3736 | 0.8601 (ns) |
|  | Within Groups | 94.87 | 18 | 5.271 |  |  |
|  | Total | 104.7 | 23 |  |  |  |
|  |  |  |  |  |  |  |
| Zn | Between Groups | 455.1 | 5 | 91.02 | 0.6440 | 0.6694 (ns) |
|  | Within Groups | 2544 | 18 | 141.3 |  |  |
|  | Total | 2999 | 23 |  |  |  |

**Table S10.** Results from a post-hoc pairwise Sidak’s multiple comparisons test of mean elemental concentrations (Se, Cu, Zn) between cortex and medulla ROIs in mouse kidney from each dietary group. Biological replicates: *n =* 4.

| **Element** | **Comparison Group** | **Mean Diff.** | **SE of Diff.** | **95% CI of Diff.** | | **P** |
| --- | --- | --- | --- | --- | --- | --- |
|  |  |  |  | **Lower Bound** | **Upper Bound** |  |
| Se | Control | 1.214 | 1.478 | -2.674 | 5.102 | 0.8069 (ns) |
|  | SeNPs | 3.077 | 1.478 | -0.8113 | 6.965 | 0.1477 (ns) |
|  | SeN | 0.8393 | 1.478 | -0.3049 | 4.727 | 0.9244 (ns) |
|  |  |  |  |  |  |  |
| Cu | Control | 0.7655 | 1.623 | -3.506 | 5.037 | 0.9545 (ns) |
|  | SeNPs | 1.603 | 1.623 | -2.668 | 5.874 | 0.7078 (ns) |
|  | SeN | -0.6520 | 1.623 | -4.923 | 3.619 | 0.9710 (ns) |
|  |  |  |  |  |  |  |
| Zn | Control | 4.439 | 8.406 | -17.68 | 26.56 | 0.9379 (ns) |
|  | SeNPs | 11.87 | 8.406 | -10.25 | 33.99 | 0.4385 (ns) |
|  | SeN | -5.376 | 8.406 | -27.49 | 16.74 | 0.8966 (ns) |


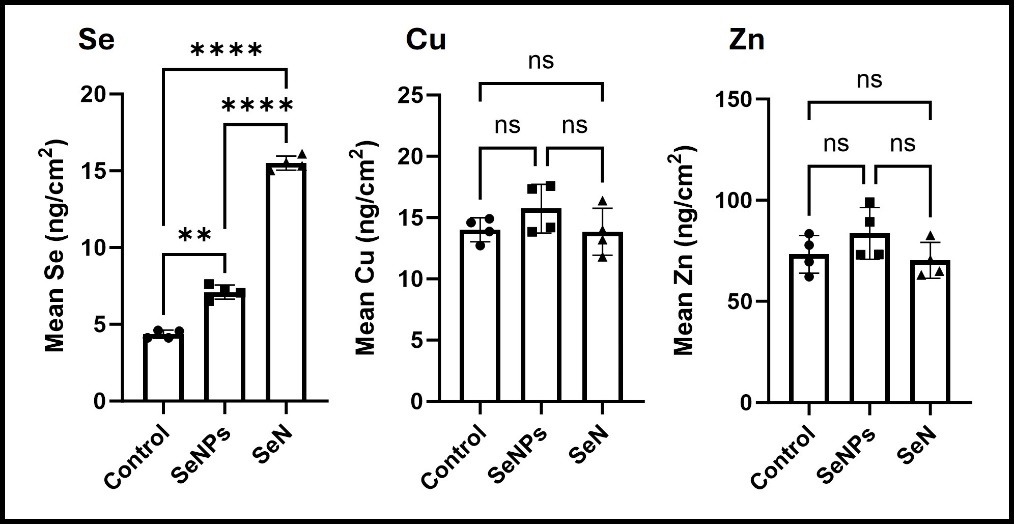


**Figure S17.** Comparisons of measured mean elemental concentrations of Se, Cu, and Zn in glomeruli in mouse kidneys from each dietary group. Data represents mean ± SD for *n =* 4 biological replicates, with measurements representing the average elemental content from multiple individual glomeruli (*n =* 4). The level of statistical significance for each comparison is indicated. Significant = *P* < 0.05 as determined by ordinary one-way ANOVA and Tukey multiple comparisons test, assuming normality and equal variance. Notation “ns” indicates no significance, and symbols *, **, ***, **** indicate *P* < 0.05 < 0.01 < 0.001 < 0.0001, respectively.

**Table S11.** Results from an ordinary one-way ANOVA to compare mean elemental concentrations (Se, Cu, Zn) in glomeruli ROIs identified in kidney from each dietary group. Biological replicates: *n* = 4, average of *n =* 4 measurements from individual glomeruli for each kidney. F critical = 4.26, for significance level = 0.05.

| **Element** |  | **SS** | **DF** | **MS** | **F** | **P** |
| --- | --- | --- | --- | --- | --- | --- |
| Se | Between Groups | 269.8 | 2 | 134.9 | 171.9 | < 0.0001 (****) |
|  | Within Groups | 7.062 | 9 | 0.7847 |  |  |
|  | Total | 276.8 | 11 |  |  |  |
|  |  |  |  |  |  |  |
| Cu | Between Groups | 8.772 | 2 | 4.386 | 1.530 | 0.2679 (ns) |
|  | Within Groups | 25.80 | 9 | 2.866 |  |  |
|  | Total | 34.57 | 11 |  |  |  |
|  |  |  |  |  |  |  |
| Zn | Between Groups | 392.3 | 2 | 196.2 | 1.789 | 0.2217 (ns) |
|  | Within Groups | 986.7 | 9 | 109.6 |  |  |
|  | Total | 1379 | 11 |  |  |  |

**Table S12 .** Results from a post-hoc Tukey’s multiple comparisons test of mean elemental concentrations (Se, Cu, Zn) in cortex ROIs identified in kidney from each dietary group. Biological replicates: *n* = 4, average of *n =* 4 measurements from individual glomeruli for each kidney.

| **Element** | **Comparison** | **Mean Diff.** | **SE of Diff.** | **95% CI of Diff.** | | **P** |
| --- | --- | --- | --- | --- | --- | --- |
|  |  |  |  | **Lower Bound** | **Upper Bound** |  |
| Se | Control vs SeNPs | -2.748 | 0.6264 | -4.497 | -0.9994 | 0.0045 (**) |
|  | Control vs SeN | -11.15 | 0.6264 | -12.90 | -9.398 | < 0.0001 (****) |
|  | SeNPs vs SeN | -8.398 | 0.6264 | -10.15 | -6.649 | < 0.0001 (****) |
|  |  |  |  |  |  |  |
| Cu | Control vs SeNPs | -1.722 | 1.197 | -5.064 | 1.620 | 0.3631 (ns) |
|  | Control vs SeN | 0.1712 | 1.197 | -3.171 | 3.514 | 0.9888 (ns) |
|  | SeNPs vs SeN | 1.893 | 1.197 | -1.449 | 5.236 | 0.3018 (ns) |
|  |  |  |  |  |  |  |
| Zn | Control vs SeNPs | -10.40 | 7.404 | -31.07 | 10.28 | 0.3790 (ns) |
|  | Control vs SeN | 2.929 | 7.404 | -17.74 | 23.60 | 0.9181 (ns) |
|  | SeNPs vs SeN | 13.33 | 7.404 | -7.346 | 34.00 | 0.2239 (ns) |


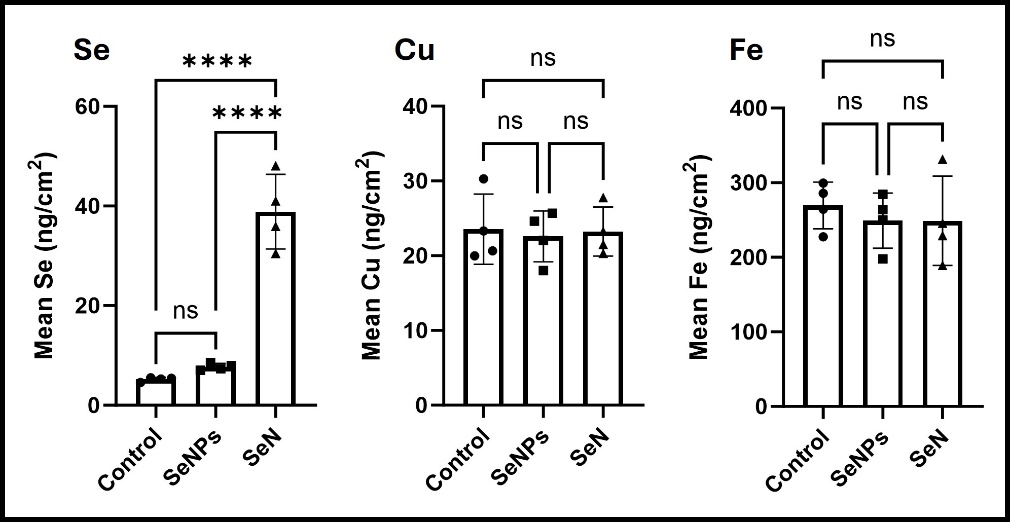


**Figure S18.** Comparisons of measured mean elemental concentrations of Se, Cu, and Fe in mouse livers (total area) from each dietary group. Data represents mean ± SD for *n =* 4 biological replicates (individual data points are provided). The level of statistical significance for each comparison is indicated. Significant = *P* < 0.05 as determined by an ordinary one-way ANOVA and Tukey multiple comparisons test, assuming normality and equal variance. Notation “ns” indicates no significance, and symbols *, **, ***, **** indicate *P* < 0.05 < 0.01 < 0.001 < 0.0001, respectively.

**Table S13.** Results from an ordinary one-way ANOVA to compare mean elemental concentrations (Se, Cu, Fe) in the total area of mouse liver from each dietary group. Biological replicates: *n* = 4. F critical = 4.26, for significance level = 0.05.

| **Element** |  | **SS** | **DF** | **MS** | **F** | **P** |
| --- | --- | --- | --- | --- | --- | --- |
| Se | Between Groups | 2813 | 2 | 1406 | 73.99 | < 0.0001 (****) |
|  | Within Groups | 171.0 | 9 | 19.01 |  |  |
|  | Total | 2984 | 11 |  |  |  |
|  |  |  |  |  |  |  |
| Cu | Between Groups | 1.935 | 2 | 0.9673 | 0.06534 | 0.9372 (ns) |
|  | Within Groups | 133.2 | 9 | 14.80 |  |  |
|  | Total | 135.2 | 11 |  |  |  |
|  |  |  |  |  |  |  |
| Fe | Between Groups | 1111 | 2 | 555.3 | 0.2801 | 0.7621 (ns) |
|  | Within Groups | 17843 | 9 | 1983 |  |  |
|  | Total | 18954 | 11 |  |  |  |

**Table S14 .** Results from a post-hoc Tukey’s multiple comparisons test of mean elemental concentrations (Se, Cu, Fe) in the total area of mouse liver from each dietary group. Biological replicates: *n* = 4.

| **Element** | **Comparison** | **Mean Diff.** | **SE of Diff.** | **95% CI of Diff.** | | **P** |
| --- | --- | --- | --- | --- | --- | --- |
|  |  |  |  | **Lower Bound** | **Upper Bound** |  |
| Se | Control vs SeNPs | -2.525 | 3.083 | -11.13 | 6.082 | 0.7013 (ns) |
|  | Control vs SeN | -33.66 | 3.083 | -42.27 | -25.06 | < 0.0001 (****) |
|  | SeNPs vs SeN | -31.14 | 3.083 | -39.75 | -22.53 | < 0.0001 (****) |
|  |  |  |  |  |  |  |
| Cu | Control vs SeNPs | 0.9675 | 2.721 | -6.628 | 8.563 | 0.9332 (ns) |
|  | Control vs SeN | 0.3307 | 2.721 | -7.265 | 7.927 | 0.9919 (ns) |
|  | SeNPs vs SeN | -0.6367 | 2.721 | -8.233 | 6.959 | 0.9704 (ns) |
|  |  |  |  |  |  |  |
| Fe | Control vs SeNPs | 20.31 | 31.48 | -67.60 | 108.2 | 0.7997 (ns) |
|  | Control vs SeN | 20.51 | 31.48 | -67.40 | 108.4 | 0.7963 (ns) |
|  | SeNPs vs SeN | 0.2012 | 31.48 | -87.70 | 88.11 | > 0.9999 (ns) |


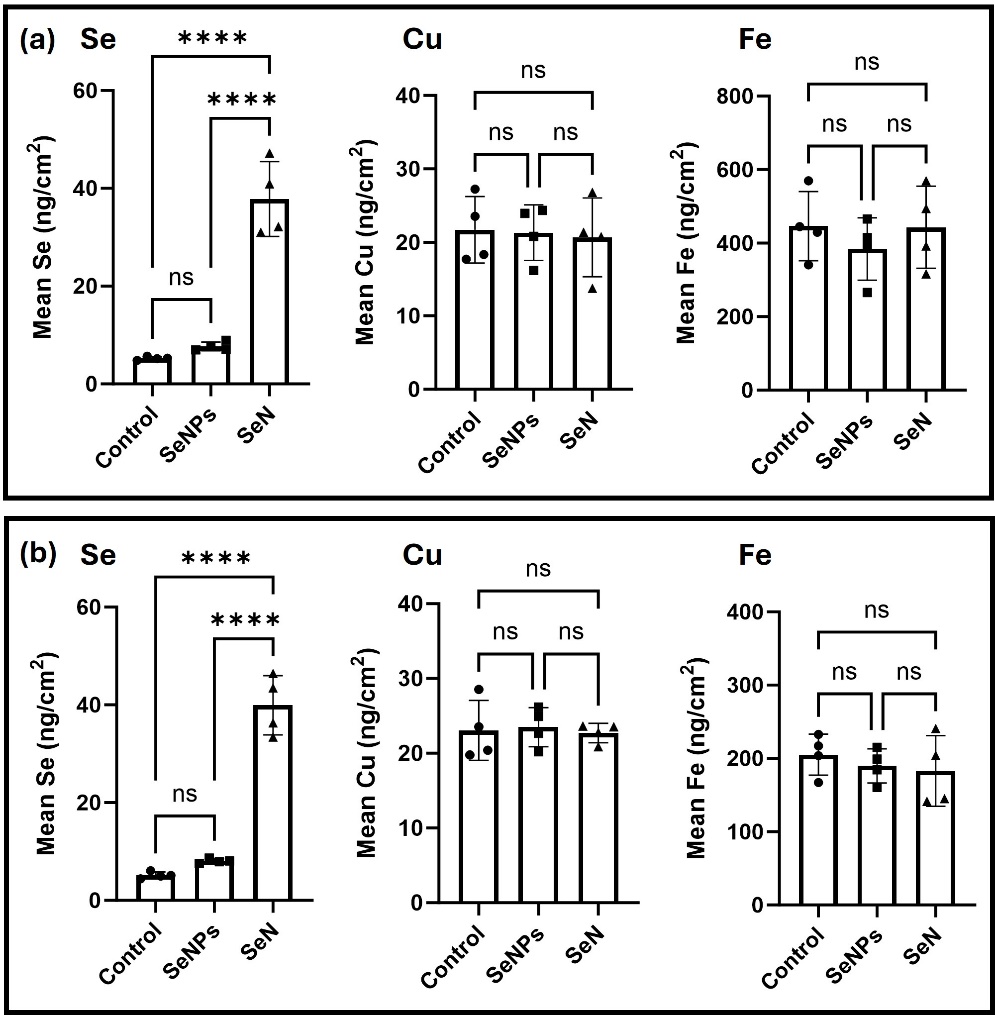


**Figure S19.** Comparisons of measured mean elemental concentrations of Se, Cu, and Fe in **(a)** portal vein and **(b)** central vein ROIs in mouse livers from each dietary group. Data represents mean ± SD for *n =* 4 biological replicates, with measurements representing the average elemental content from multiple individual portal vein and central vein ROIs (*n =* 4 each). The level of statistical significance for each comparison is indicated. Significant = *P* < 0.05 as determined by nested one-way ANOVA and Tukey multiple comparisons test, assuming normality and equal variance. Notation “ns” indicates no significance, and symbols *, **, ***, **** indicate *P* < 0.05 < 0.01 < 0.001 < 0.0001, respectively.

**Table S15.** Results from an ordinary one-way ANOVA to compare mean elemental concentrations (Se, Cu, Fe) in portal vein ROIs in mouse liver from each dietary group. Biological replicates: *n* = 4, average of *n =* 4 measurements from individual portal veins for each liver. F critical = 4.26, for significance level = 0.05.

| **Element** |  | **SS** | **DF** | **MS** | **F** | **P** |
| --- | --- | --- | --- | --- | --- | --- |
| Se | Between Groups | 2638 | 2 | 1319 | 66.35 | < 0.0001 (****) |
|  | Within Groups | 178.9 | 9 | 19.88 |  |  |
|  | Total | 2817 | 11 |  |  |  |
|  |  |  |  |  |  |  |
| Cu | Between Groups | 2.213 | 2 | 1.107 | 0.05235 | 0.9493 (ns) |
|  | Within Groups | 190.2 | 9 | 21.14 |  |  |
|  | Total | 192.4 | 11 |  |  |  |
|  |  |  |  |  |  |  |
| Fe | Between Groups | 9793 | 2 | 4896 | 0.5147 | 0.6143 (ns) |
|  | Within Groups | 85620 | 9 | 9513 |  |  |
|  | Total | 95412 | 11 |  |  |  |

**Table S16 .** Results from a post-hoc Tukey’s multiple comparisons test of mean elemental concentrations (Se, Cu, Fe) in portal vein ROIs in mouse liver from each dietary group. Biological replicates: *n* = 4, average of *n =* 4 measurements from individual portal veins for each tissue.

| **Element** | **Comparison** | **Mean Diff.** | **SE of Diff.** | **95% CI of Diff.** | | **P** |
| --- | --- | --- | --- | --- | --- | --- |
|  |  |  |  | **Lower Bound** | **Upper Bound** |  |
| Se | Control vs SeNPs | -2.455 | 3.153 | -11.26 | 6.348 | 0.7247 (ns) |
|  | Control vs SeN | -32.61 | 3.153 | -41.41 | -23.80 | < 0.0001 (****) |
|  | SeNPs vs SeN | -30.15 | 3.153 | -38.95 | -21.35 | < 0.0001 (****) |
|  |  |  |  |  |  |  |
| Cu | Control vs SeNPs | 0.3945 | 3.251 | -8.682 | 9.471 | 0.9919 (ns) |
|  | Control vs SeN | 1.042 | 3.251 | -8.035 | 10.12 | 0.9453 (ns) |
|  | SeNPs vs SeN | 0.6473 | 3.251 | -8.429 | 9.724 | 0.9784 (ns) |
|  |  |  |  |  |  |  |
| Fe | Control vs SeNPs | 62.12 | 68.97 | -130.4 | 254.7 | 0.6533 (ns) |
|  | Control vs SeN | 3.168 | 68.97 | -189.4 | 195.7 | 0.9988 (ns) |
|  | SeNPs vs SeN | -58.95 | 68.97 | -251.5 | 133.6 | 0.6803 (ns) |

**Table S17.** Results from an ordinary one-way ANOVA to compare mean elemental concentrations (Se, Cu, Fe) in central vein ROIs in mouse liver from each dietary group. Biological replicates: *n* = 4, average of *n =* 4 measurements from individual central veins for each liver. F critical = 4.26, for significance level = 0.05.

| **Element** |  | **SS** | **DF** | **MS** | **F** | **P** |
| --- | --- | --- | --- | --- | --- | --- |
| Se | Between Groups | 2975 | 2 | 1488 | 119.5 | < 0.0001 (****) |
|  | Within Groups | 112.0 | 9 | 12.45 |  |  |
|  | Total | 3087 | 11 |  |  |  |
|  |  |  |  |  |  |  |
| Cu | Between Groups | 1.238 | 2 | 0.6188 | 0.07529 | 0.9281 (ns) |
|  | Within Groups | 73.97 | 9 | 8.218 |  |  |
|  | Total | 75.20 | 11 |  |  |  |
|  |  |  |  |  |  |  |
| Fe | Between Groups | 1044 | 2 | 522.1 | 0.4297 | 0.6634 (ns) |
|  | Within Groups | 10936 | 9 | 1215 |  |  |
|  | Total | 11981 | 11 |  |  |  |

**Table S18 .** Results from a post-hoc Tukey’s multiple comparisons test of mean elemental concentrations (Se, Cu, Fe) in central vein ROIs in mouse liver from each dietary group. Biological replicates: *n* = 4, average of *n =* 4 measurements from individual central veins for each liver.

| **Element** | **Comparison** | **Mean Diff.** | **SE of Diff.** | **95% CI of Diff.** | | **P** |
| --- | --- | --- | --- | --- | --- | --- |
|  |  |  |  | **Lower Bound** | **Upper Bound** |  |
| Se | Control vs SeNPs | -2.926 | 2.495 | -9.892 | 4.040 | 0.4971 (ns) |
|  | Control vs SeN | -34.77 | 2.495 | -41.73 | -27.80 | < 0.0001 (****) |
|  | SeNPs vs SeN | -31.84 | 2.495 | -38.81 | -24.88 | < 0.0001 (****) |
|  |  |  |  |  |  |  |
| Cu | Control vs SeNPs | -0.4290 | 2.027 | -6.089 | 5.231 | 0.9757 (ns) |
|  | Control vs SeN | 0.3565 | 2.027 | -5.303 | 6.016 | 0.9831 (ns) |
|  | SeNPs vs SeN | 0.7855 | 2.027 | -4.874 | 6.445 | 0.9213 (ns) |
|  |  |  |  |  |  |  |
| Fe | Control vs SeNPs | 15.37 | 24.65 | -53.45 | 84.19 | 0.8112 (ns) |
|  | Control vs SeN | 22.33 | 24.65 | -46.49 | 91.15 | 0.6503 (ns) |
|  | SeNPs vs SeN | 6.955 | 24.65 | -61.87 | 75.78 | 0.9573 (ns) |


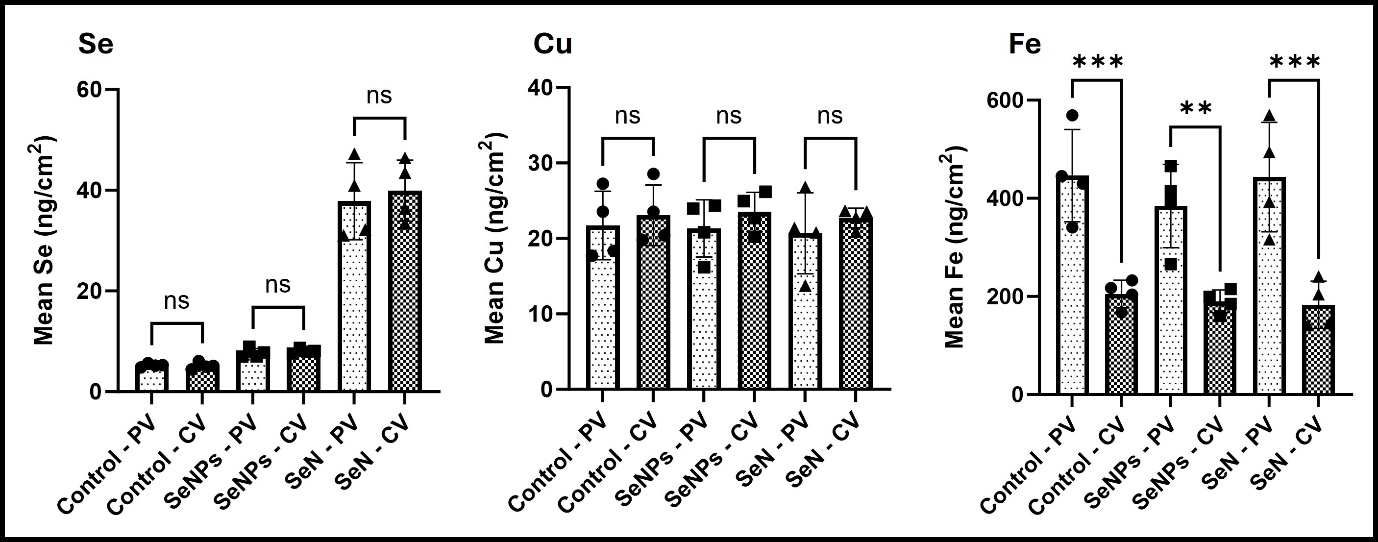


**Figure S20.** Selected pairwise comparisons between measured mean elemental concentrations of Se, Cu, and Fe in the portal vein (PV) and central vein (CV) ROIs in mouse livers from each dietary group. Data represents mean ± SD for *n =* 4 biological replicates, with measurements representing the average elemental content from multiple individual portal vein and central vein ROIs (*n =* 4 each). The level of statistical significance for each comparison is indicated. Significant = *P* < 0.05 as determined by nested one-way ANOVA and post-hoc pairwise comparisons with Sidak’s correction, assuming normality and equal variance. Notation “ns” indicates no significance, and symbols *, **, ***, **** indicate *P* < 0.05 < 0.01 < 0.001 < 0.0001, respectively.

**Table S19.** Results from an ordinary one-way ANOVA to compare mean elemental concentrations (Se, Cu, Fe) between portal vein and central vein ROIs in mouse liver from each dietary group. Biological replicates: *n =* 4, average of *n =* 4 measurements from individual veins for each liver. F critical = 2.77, for significance level = 0.05.

| **Element** |  | **SS** | **DF** | **MS** | **F** | **P** |
| --- | --- | --- | --- | --- | --- | --- |
| Se | Between Groups | 5617 | 5 | 1123 | 69.50 | < 0.0001 (****) |
|  | Within Groups | 291.0 | 18 | 16.16 |  |  |
|  | Total | 5908 | 23 |  |  |  |
|  |  |  |  |  |  |  |
| Cu | Between Groups | 24.09 | 5 | 4.818 | 0.3283 | 0.8893 (ns) |
|  | Within Groups | 264.2 | 18 | 14.68 |  |  |
|  | Total | 288.3 | 23 |  |  |  |
|  |  |  |  |  |  |  |
| Fe | Between Groups | 333550 | 5 | 66710 | 12.44 | < 0.0001 (****) |
|  | Within Groups | 96556 | 18 | 5364 |  |  |
|  | Total | 430106 | 23 |  |  |  |

**Table S20.** Results from a post-hoc pairwise Sidak’s multiple comparisons test of mean elemental concentrations (Se, Cu, Fe) between portal vein and central vein ROIs in mouse liver from each dietary group. Biological replicates: *n =* 4, average of *n =* 4 measurements from individual veins for each liver.

| **Element** | **Comparison Group** | **Mean Diff.** | **SE of Diff.** | **95% CI of Diff.** | | **P** |
| --- | --- | --- | --- | --- | --- | --- |
|  |  |  |  | **Lower Bound** | **Upper Bound** |  |
| Se | Control | 0.08425 | 2.843 | -7.396 | 7.564 | > 0.9999 (ns) |
|  | SeNPs | -0.3873 | 2.843 | -7.867 | 7.093 | 0.9988 (ns) |
|  | SeN | -2.077 | 2.843 | -9.557 | 5.403 | 0.8548 (ns) |
|  |  |  |  |  |  |  |
| Cu | Control | -1.352 | 2.709 | -8.479 | 5.776 | 0.9468 (ns) |
|  | SeNPs | -2.175 | 2.709 | -9.303 | 4.952 | 0.8172 (ns) |
|  | SeN | -2.037 | 2.709 | -9.165 | 5.091 | 0.8441 (ns) |
|  |  |  |  |  |  |  |
| Fe | Control | 241.1 | 51.79 | 104.9 | 377.4 | 0.0006 (***) |
|  | SeNPs | 194.4 | 51.79 | 58.10 | 330.6 | 0.0044 (**) |
|  | SeN | 260.3 | 51.79 | 124.0 | 396.5 | 0.0003 (***) |


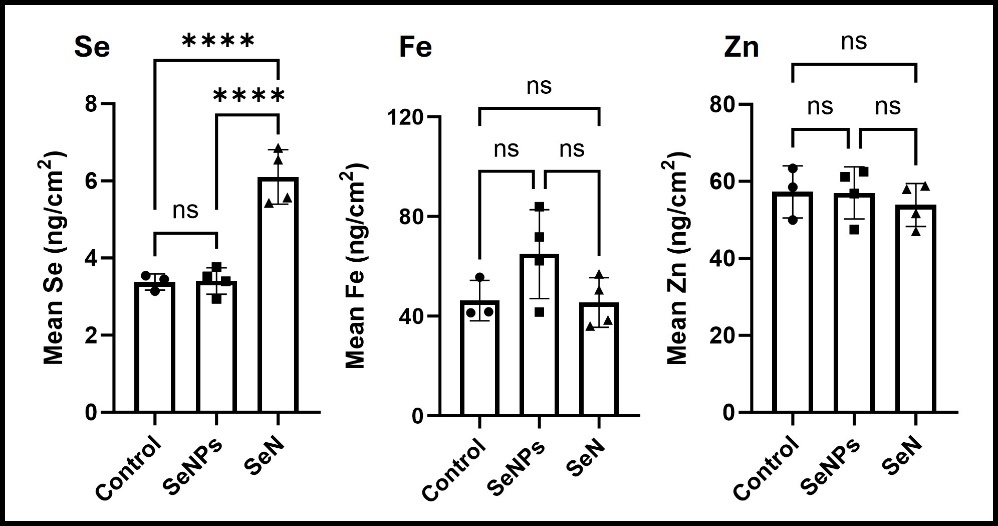


**Figure S21.** Comparisons of measured mean elemental concentrations of Se, Fe and Zn in mouse testis (total area) from each dietary group. Data represents mean ± SD for *n =* 3 or *n =* 4 biological replicates (individual data points are provided). The level of statistical significance for each comparison is indicated. Significant = *P* < 0.05 as determined by an ordinary one-way ANOVA and Tukey multiple comparisons test, assuming normality and equal variance. Notation “ns” indicates no significance, and symbols *, **, ***, **** indicate *P* < 0.05 < 0.01 < 0.001 < 0.0001, respectively.

**Table S21.** Results from an ordinary one-way ANOVA to compare mean elemental concentrations (Se, Fe, Zn) in the total area of mouse testis from each dietary group. Biological replicates: *n* = 3 or *n =* 4. F critical = 4.46, for significance level = 0.05.

| **Element** |  | **SS** | **DF** | **MS** | **F** | **P** |
| --- | --- | --- | --- | --- | --- | --- |
| Se | Between Groups | 18.68 | 2 | 9.338 | 38.36 | < 0.0001 (****) |
|  | Within Groups | 1.948 | 8 | 0.2434 |  |  |
|  | Total | 20.62 | 10 |  |  |  |
|  |  |  |  |  |  |  |
| Fe | Between Groups | 931.2 | 2 | 465.6 | 2.690 | 0.1278 (ns) |
|  | Within Groups | 1384 | 8 | 173.1 |  |  |
|  | Total | 2316 | 10 |  |  |  |
|  |  |  |  |  |  |  |
| Zn | Between Groups | 26.82 | 2 | 13.41 | 0.3321 | 0.7269 (ns) |
|  | Within Groups | 323.1 | 8 | 40.38 |  |  |
|  | Total | 349.9 | 10 |  |  |  |

**Table S22 .** Results from a post-hoc Tukey’s multiple comparisons test of mean elemental concentrations (Se, Fe, Zn) in the total area of mouse testis from each dietary group. Biological replicates: *n* = 3 or *n =* 4.

| **Element** | **Comparison** | **Mean Diff.** | **SE of Diff.** | **95% CI of Diff.** | | **P** |
| --- | --- | --- | --- | --- | --- | --- |
|  |  |  |  | **Lower Bound** | **Upper Bound** |  |
| Se | Control vs SeNPs | -0.02708 | 0.3768 | -1.104 | 1.050 | 0.9972 (ns) |
|  | Control vs SeN | -2.724 | 0.3768 | -3.801 | -1.647 | 0.0002 (***) |
|  | SeNPs vs SeN | -2.697 | 0.3489 | -3.694 | -1.700 | 0.0001 (***) |
|  |  |  |  |  |  |  |
| Fe | Control vs SeNPs | -18.68 | 10.05 | -47.39 | 10.03 | 0.2120 (ns) |
|  | Control vs SeN | 0.7603 | 10.05 | -27.95 | 29.47 | 0.9968 (ns) |
|  | SeNPs vs SeN | 19.44 | 9.302 | -7.138 | 46.02 | 0.1534 (ns) |
|  |  |  |  |  |  |  |
| Zn | Control vs SeNPs | 0.2707 | 4.854 | -13.60 | 14.14 | 0.9983 (ns) |
|  | Control vs SeN | 3.393 | 4.854 | -10.48 | 17.26 | 0.7707 (ns) |
|  | SeNPs vs SeN | 3.123 | 4.493 | -9.717 | 15.96 | 0.7730 (ns) |


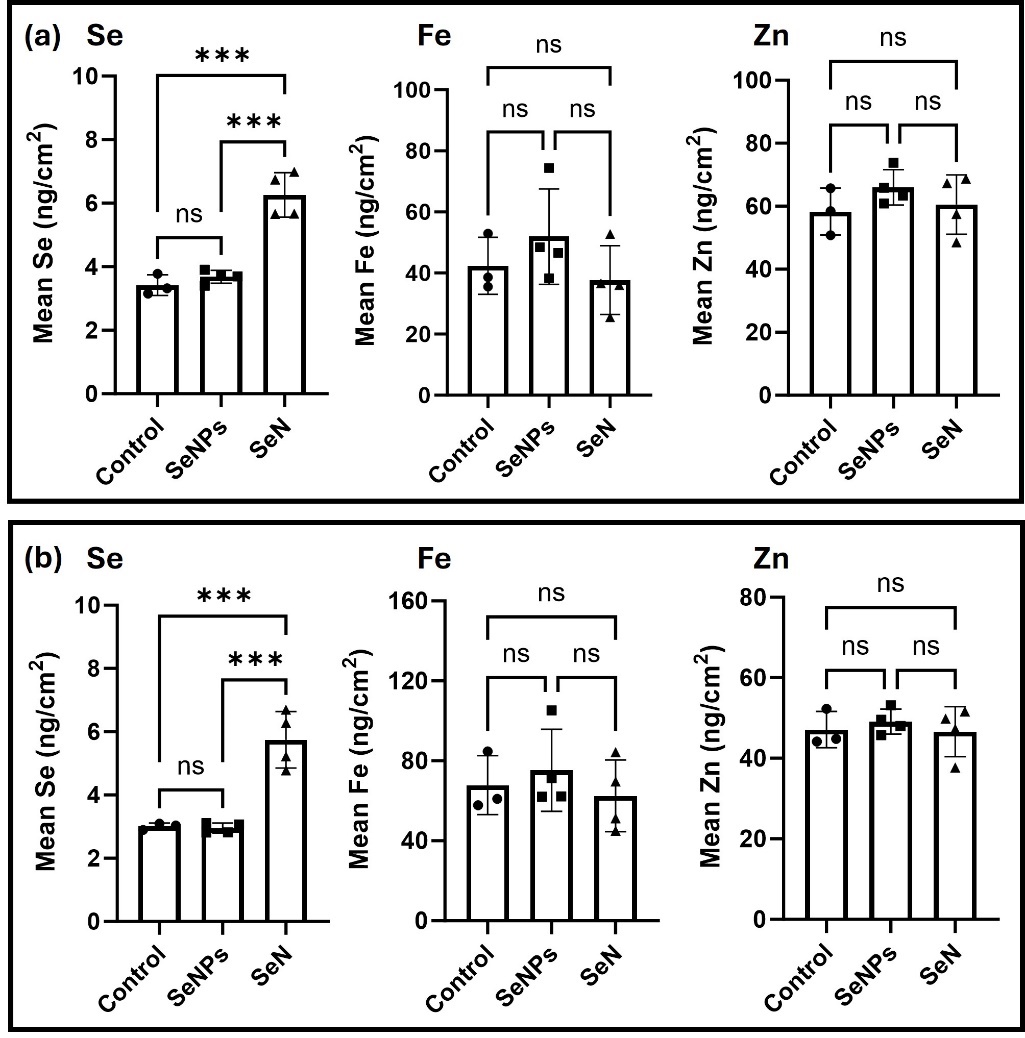


**Figure S22.** Comparisons of measured mean elemental concentrations of Se, Fe and Zn in **(a)** seminiferous tubules and **(b)** interstitial tissue ROIs in mouse testis from each dietary group. Data represents mean ± SD for *n =* 3 or *n =* 4 biological replicates, with measurements representing the average elemental content from multiple individual seminiferous tubules and interstitial tissue ROIs (*n =* 4 each). The level of statistical significance for each comparison is indicated. Significant = *P* < 0.05 as determined by ordinary one-way ANOVA and Tukey multiple comparisons test, assuming normality and equal variance. Notation “ns” indicates no significance, and symbols *, **, ***, **** indicate *P* < 0.05 < 0.01 < 0.001 < 0.0001, respectively.

**Table S23.** Results from an ordinary one-way ANOVA to compare mean elemental concentrations (Se, Fe, Zn) in seminiferous tubules ROIs in mouse testis from each dietary group. Biological replicates: *n* = 3 or *n =* 4, average of *n =* 4 measurements from individual tubules for each testis. F critical = 4.46, for significance level = 0.05.

| **Element** |  | **SS** | **DF** | **MS** | **F** | **P** |
| --- | --- | --- | --- | --- | --- | --- |
| Se | Between Groups | 18.60 | 2 | 9.299 | 41.35 | < 0.0001 (****) |
|  | Within Groups | 1.799 | 8 | 0.2249 |  |  |
|  | Total | 20.40 | 10 |  |  |  |
|  |  |  |  |  |  |  |
| Fe | Between Groups | 416.1 | 2 | 208.1 | 1.294 | 0.3258 (ns) |
|  | Within Groups | 1286 | 8 | 160.7 |  |  |
|  | Total | 1702 | 10 |  |  |  |
|  |  |  |  |  |  |  |
| Zn | Between Groups | 113.0 | 2 | 56.49 | 0.9618 | 0.4224 (ns) |
|  | Within Groups | 469.8 | 8 | 58.73 |  |  |
|  | Total | 582.8 | 10 |  |  |  |

**Table S24.** Results from a post-hoc Tukey’s multiple comparisons test of mean elemental concentrations (Se, Fe, Zn) in seminiferous tubules ROIs in mouse testis from each dietary group. Biological replicates: *n* = 3 or *n =* 4, average of *n =* 4 measurements from individual tubules for each testis.

| **Element** | **Comparison** | **Mean Diff.** | **SE of Diff.** | **95% CI of Diff.** | | **P** |
| --- | --- | --- | --- | --- | --- | --- |
|  |  |  |  | **Lower Bound** | **Upper Bound** |  |
| Se | Control vs SeNPs | -0.2648 | 0.3622 | -1.300 | 0.7701 | 0.7527 (ns) |
|  | Control vs SeN | -2.846 | 0.3622 | -3.880 | -1.811 | 0.0001 (***) |
|  | SeNPs vs SeN | -2.581 | 0.3353 | -3.539 | -1.623 | 0.0001 (***) |
|  |  |  |  |  |  |  |
| Fe | Control vs SeNPs | -9.564 | 9.683 | -37.23 | 19.10 | 0.6044 (ns) |
|  | Control vs SeN | 4.628 | 9.683 | -23.04 | 32.30 | 0.8834 (ns) |
|  | SeNPs vs SeN | 14.19 | 8.964 | -11.42 | 39.81 | 0.3067 (ns) |
|  |  |  |  |  |  |  |
| Zn | Control vs SeNPs | -7.673 | 5.853 | -24.40 | 9.052 | 0.4284 (ns) |
|  | Control vs SeN | -2.210 | 5.853 | -18.94 | 14.51 | 0.9251 (ns) |
|  | SeNPs vs SeN | 5.463 | 5.419 | -10.02 | 20.95 | 0.5926 (ns) |

**Table S25.** Results from an ordinary one-way ANOVA to compare mean elemental concentrations (Se, Fe, Zn) in interstitial tissue ROIs in mouse testis from each dietary group. Biological replicates: *n* = 3 or *n =* 4, average of *n =* 4 measurements from individual interstitial tissue regions for each testis. F critical = 4.46, for significance level = 0.05.

| **Element** |  | **SS** | **DF** | **MS** | **F** | **P** |
| --- | --- | --- | --- | --- | --- | --- |
| Se | Between Groups | 19.46 | 2 | 9.732 | 31.31 | 0.0002 (***) |
|  | Within Groups | 2.487 | 8 | 0.3109 |  |  |
|  | Total | 21.95 | 10 |  |  |  |
|  |  |  |  |  |  |  |
| Fe | Between Groups | 326.5 | 2 | 163.3 | 0.4928 | 0.6283 (ns) |
|  | Within Groups | 2650 | 8 | 331.3 |  |  |
|  | Total | 2977 | 10 |  |  |  |
|  |  |  |  |  |  |  |
| Zn | Between Groups | 13.84 | 2 | 6.921 | 0.2980 | 0.7502 (ns) |
|  | Within Groups | 185.8 | 8 | 23.22 |  |  |
|  | Total | 199.6 | 10 |  |  |  |

**Table S26.** Results from a post-hoc Tukey’s multiple comparisons test of mean elemental concentrations (Se, Fe, Zn) in interstitial tissue ROIs in mouse testis from each dietary group. Biological replicates: *n* = 3 or *n =* 4, average of *n =* 4 measurements from individual interstitial tissue regions for each testis.

| **Element** | **Comparison** | **Mean Diff.** | **SE of Diff.** | **95% CI of Diff.** | | **P** |
| --- | --- | --- | --- | --- | --- | --- |
|  |  |  |  | **Lower Bound** | **Upper Bound** |  |
| Se | Control vs SeNPs | 0.05617 | 0.4258 | -1.161 | 1.273 | 0.9905 (ns) |
|  | Control vs SeN | -2.733 | 0.4258 | -3.950 | -1.516 | 0.0005 (***) |
|  | SeNPs vs SeN | -2.789 | 0.3942 | -3.916 | -1.662 | 0.0003 (***) |
|  |  |  |  |  |  |  |
| Fe | Control vs SeNPs | -7.407 | 13.90 | -47.13 | 32.31 | 0.8578 (ns) |
|  | Control vs SeN | 5.324 | 13.90 | -34.40 | 45.05 | 0.9231 (ns) |
|  | SeNPs vs SeN | 12.73 | 12.87 | -24.04 | 49.51 | 0.6035 (ns) |
|  |  |  |  |  |  |  |
| Zn | Control vs SeNPs | -2.012 | 3.681 | -12.53 | 8.506 | 0.8511 (ns) |
|  | Control vs SeN | 0.4978 | 3.681 | -10.02 | 11.02 | 0.9900 (ns) |
|  | SeNPs vs SeN | 2.509 | 3.408 | -7.228 | -7.228 | 0.7498 (ns) |


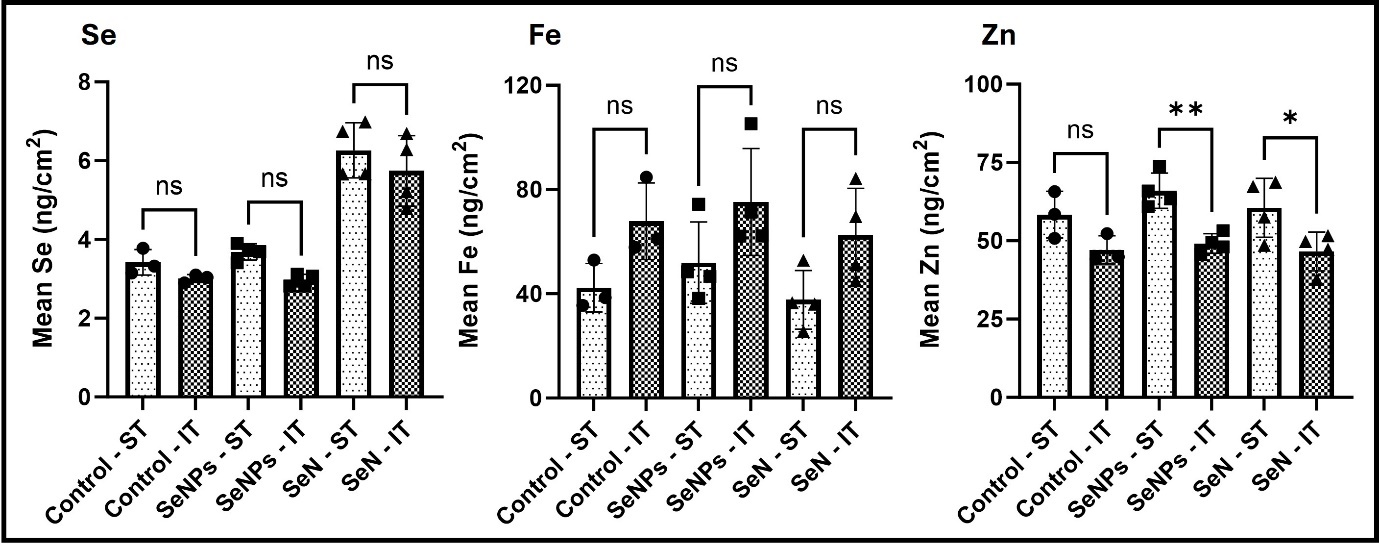


**Figure S23.** Selected pairwise comparisons between measured mean elemental concentrations of Se, Fe and Zn in the seminiferous tubules (ST) and interstitial tissue (IT) ROIs in mouse testis from each dietary group. Data represents mean ± SD for *n =* 3 or *n =* 4 biological replicates, with measurements representing the average elemental content from multiple individual seminiferous tubules and interstitial tissue ROIs (*n =* 4 each). The level of statistical significance for each comparison is indicated. Significant = *P* < 0.05 as determined by ordinary one-way ANOVA and post-hoc pairwise comparisons with Sidak’s correction, assuming normality and equal variance. Notation “ns” indicates no significance, and symbols *, **, ***, **** indicate *P* < 0.05 < 0.01 < 0.001 < 0.0001, respectively.

**Table S27.** Results from an ordinary one-way ANOVA to compare mean elemental concentrations (Se, Fe, Zn) between seminiferous tubules and interstitial tissue ROIs in mouse testis from each dietary group. Biological replicates: *n =* 3 or *n =* 4, average of *n =* 4 measurements from individual tubules and interstitial tissues for each testis. F critical = 2.85, for significance level = 0.05.

| **Element** |  | **SS** | **DF** | **MS** | **F** | **P** |
| --- | --- | --- | --- | --- | --- | --- |
| Se | Between Groups | 39.82 | 5 | 7.965 | 29.73 | < 0.0001 (****) |
|  | Within Groups | 4.286 | 16 | 0.2679 |  |  |
|  | Total | 44.11 | 21 |  |  |  |
|  |  |  |  |  |  |  |
| Fe | Between Groups | 4033 | 5 | 806.7 | 3.279 | 0.0315 (*) |
|  | Within Groups | 3936 | 16 | 246.0 |  |  |
|  | Total | 7969 | 21 |  |  |  |
|  |  |  |  |  |  |  |
| Zn | Between Groups | 1244 | 5 | 248.9 | 6.073 | 0.0025 (**) |
|  | Within Groups | 6556 | 16 | 40.98 |  |  |
|  | Total | 1900 | 21 |  |  |  |

**Table S28.** Results from a post-hoc pairwise Sidak’s multiple comparisons test of mean elemental concentrations (Se, Fe, Zn) between seminiferous tubules and interstitial tissue ROIs in mouse testis from each dietary group. Biological replicates: *n =* 3 or *n =* 4, average of *n =* 4 measurements from individual tubules and interstitial tissues for each testis.

| **Element** | **Comparison Group** | **Mean Diff.** | **SE of Diff.** | **95% CI of Diff.** | | **P** |
| --- | --- | --- | --- | --- | --- | --- |
|  |  |  |  | **Lower Bound** | **Upper Bound** |  |
| Se | Control | 0.4080 | 0.4226 | -0.7181 | 1.534 | 0.7237 (ns) |
|  | SeNPs | 0.7290 | 0.3660 | -0.2462 | 1.704 | 0.1792 (ns) |
|  | SeN | 0.5208 | 0.3660 | -0.4544 | 1.496 | 0.4364 (ns) |
|  |  |  |  |  |  |  |
| Fe | Control | -25.50 | 12.81 | -59.62 | 8.627 | 0.1795 (ns) |
|  | SeNPs | -23.34 | 11.09 | -52.89 | 6.212 | 0.1467 (ns) |
|  | SeN | -24.80 | 11.09 | -54.35 | 4.751 | 0.1151 (ns) |
|  |  |  |  |  |  |  |
| Zn | Control | 11.21 | 5.227 | -2.717 | 25.14 | 0.1363 (ns) |
|  | SeNPs | 16.87 | 4.526 | 4.811 | 28.93 | 0.0055 (**) |
|  | SeN | 13.92 | 4.526 | 1.857 | 25.98 | 0.0216 (*) |

**Additional HERFD-XAS data**


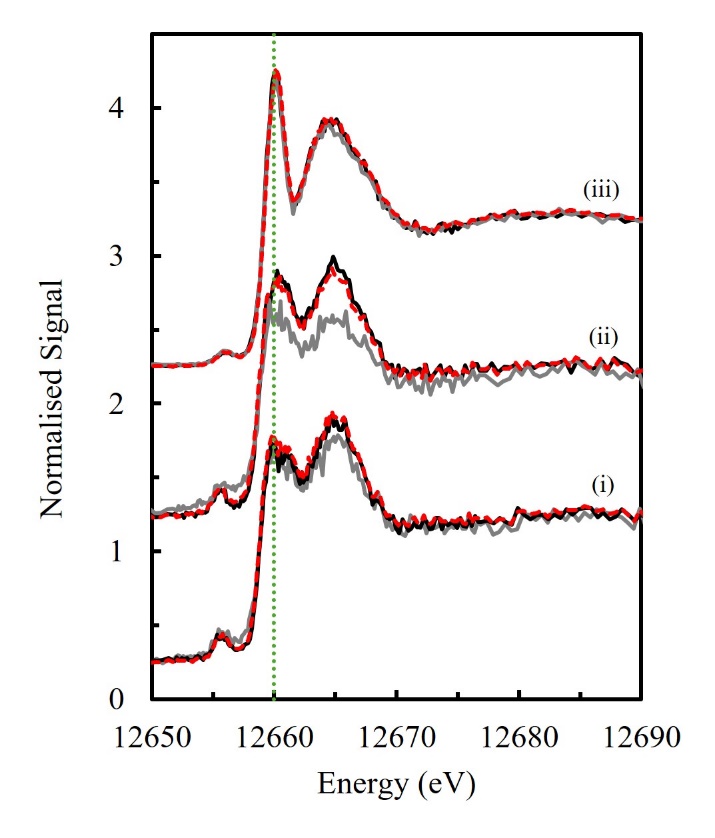


**Figure S24.** Se Kα_1_ HERFD-XAS XANES spectra of mouse kidneys depicting replicate scans from individual organisms (black and grey solid traces) and the averaged spectra (red dashed trace) from dietary groups (i) control, (ii) 10 mg Se/kg as SeNP, and (iii) 5 mg Se/kg as SeN. The green dotted line at 12,660.0 eV is included to emphasise differences in the spectral peak positions.

**Table S29.** Percent Se species in kidney and liver tissues from mice fed the control diets, fitting with Se(IV) and Se(0) models, or substituting these components for a selenourea model derived from Se2U. Fit fractions are estimated by a linear combination of model compound spectra.*^a^*

| **Tissue** | **Group** | **Percentage (%) Se species** | | | | | | | **N_tot_*^c^*** | **Residual (×10^-3^)** |
| --- | --- | --- | --- | --- | --- | --- | --- | --- | --- | --- |
|  |  | **SeO_3_^2−^ *^b^*** | **Se (0)** | **GSSeSG** | **SeMet** | **CysSe^−^** | **CuSe** | **Se2U** |  |  |
| Kidney | Se(IV) + Se (0) | 8 (1) | 8 (1) | – | 21 (1) | 35 (3) | 26 (2) | – | 0.98 | 4.04 |
|  | Se2U | – | – | – | 15 (1) | 40 (1) | 9 (3) | 34 (4) | 0.99 | 4.84 |
|  |  |  |  |  |  |  |  |  |  |  |
| Liver | Se(IV) + Se (0) | 4 (1) | – | 5 (1) | 24 (1) | 20 (3) | 42 (2) | – | 0.96 | 4.03 |
|  | Se2U | – | – | – | 23 (1) | 15 (2) | 25 (2) | 33 (4) | 0.96 | 3.78 |

*^a^* Values in parentheses are the estimated standard deviations derived from the diagonal elements of the covariance matrix and are a measure of precision. *^b^* Model spectrum was shifted by −0.25 eV to account for the shift between Kα_1_ HERFD-XAS fluorescence lines for selenite (oxidised Se) and selenomethionine (reduced Se). *^c^* N_tot_ is the sum of the fractions.


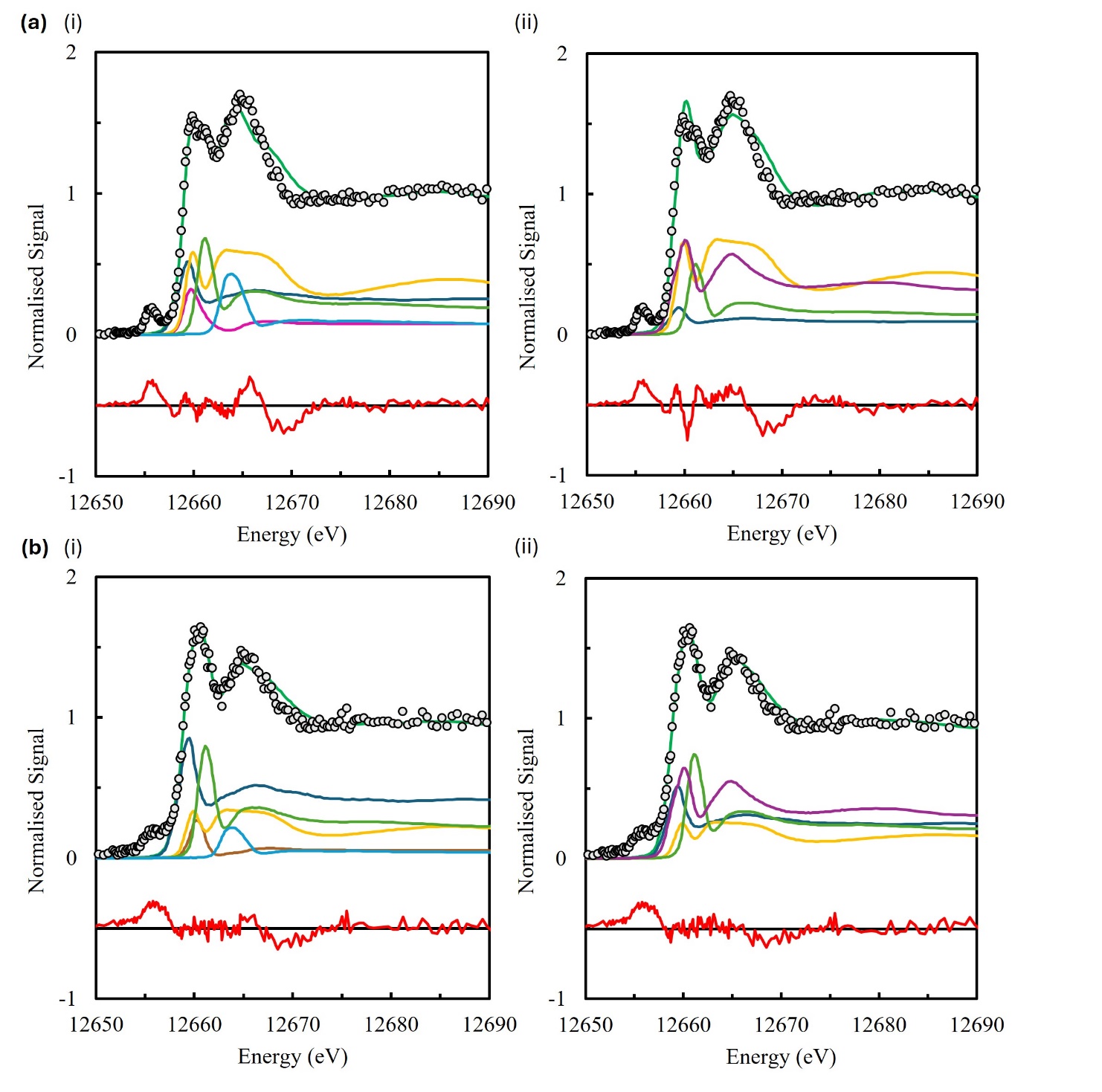


**Figure S25.** Linear combination analysis of Se Kα1-HERFD-XAS spectra of mouse **(a)** kidney and **(b)** liver from animals control diets (no added Se). For each tissue, the best fits (i) excluding or (ii) including the selenourea model are shown. The experimental data (points, grey) and the linear combination fit (green line) are displayed, along with components used in the best fit, scaled by their contributions to the fit: selenite, light blue line; elemental Se, magenta line; GSSeSG (R–S–Se–S–R’), brown line; SeMet (R–Se–R’), green line; CysSe^−^ (R–Se^−^), yellow line; CysSeSeCys (R–S–Se–Se–S–R’), orange line; CuSe (metal-selenide model), dark blue line; and Se2U (representing a selenourea, (Se=C(NR_2_)_2_)), purple line. The lower red trace shows the fit residual. Fit fractions for all components are provided in **Table S29**.


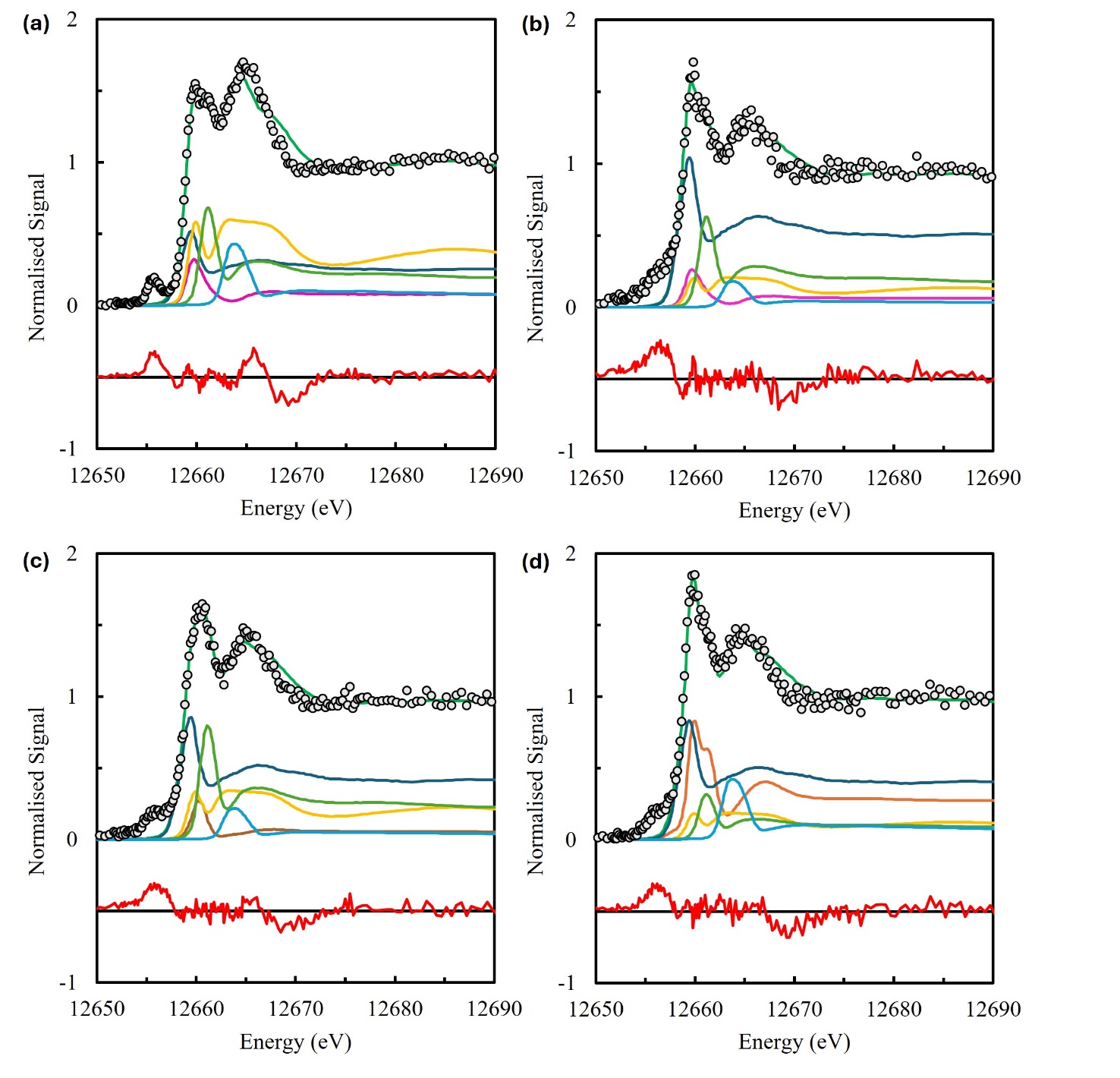


**Figure S26.** Linear combination analysis of Se Kα_1_-HERFD-XAS spectra of mouse **(a)** kidney, **(b)** whole blood, **(c)** liver, and **(d)** testis, from animals fed control diets (no added Se). The experimental data (points, grey) and the linear combination fit (green line) are displayed, along with components used in the best fit, scaled by their contributions to the fit: selenite, light blue line; elemental Se, magenta line; GSSeSG (R–S–Se–S–R’), brown line; SeMet (R–Se–R’), green line; CysSe^−^ (R–Se^−^), yellow line; CysSeSeCys (R–S–Se–Se–S–R’), orange line; and CuSe (metal-selenide model), dark blue line. The lower red trace shows the fit residual. Fit fractions for all components are provided in **Table 1**.


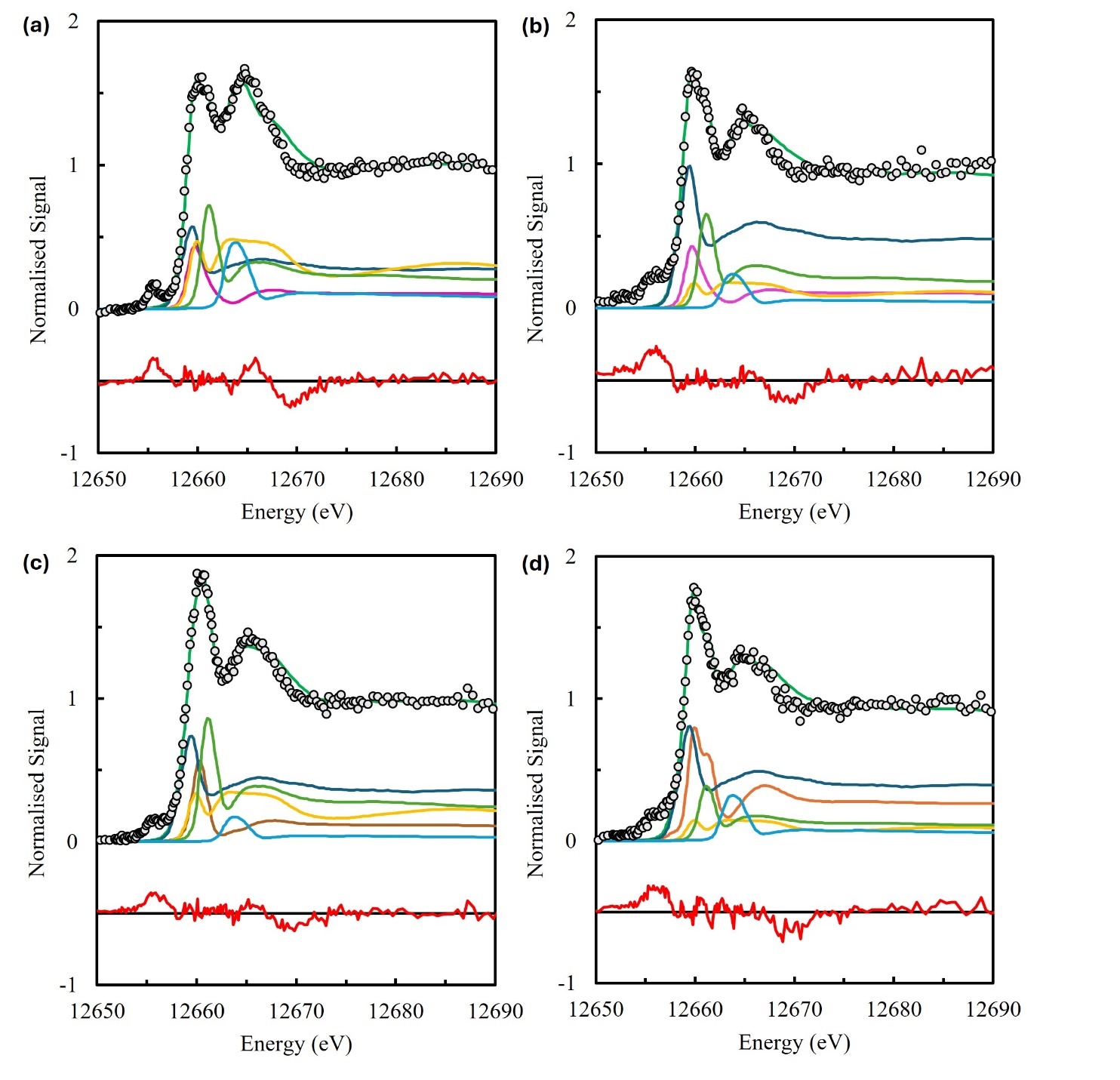


**Figure S27.** Linear combination analysis of Se Kα_1_-HERFD-XAS spectra of mouse **(a)** kidney, **(b)** whole blood, **(c)** liver, and **(d)** testis, from animals fed 10 mg Se/kg as SeNPs. The experimental data (points, grey) and the linear combination fit (green line) are displayed, along with components used in the best fit, scaled by their contributions to the fit: selenite, light blue line; elemental Se, magenta line; GSSeSG (R–S–Se–S–R’), brown line; SeMet (R–Se–R’), green line; CysSe^−^ (R–Se^−^), yellow line; CysSeSeCys (R–S–Se–Se–S–R’), orange line; and CuSe (metal-selenide model), dark blue line. The lower red trace shows the fit residual. Fit fractions for all components are provided in **Table 1**.


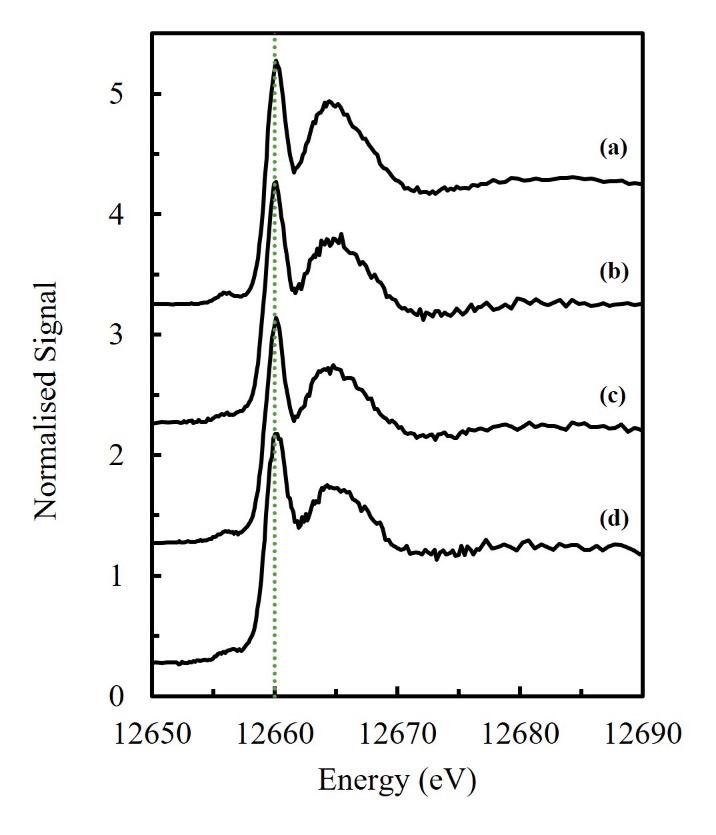


**Figure S28.** Se Kα_1_ HERFD-XAS XANES spectra of mouse tissues **(a)** kidney, **(b)** whole blood, **(c)** liver, and **(d)** testis, from animals fed 5 mg Se/kg as SeN. The kidney spectrum is the average of scans from two biological replicates show in **Figure S24**. The green dotted line at 12,660.0 eV is included to emphasise the similarities in the spectral peak positions with overlapping alignment of the major peaks occurring at this energy.


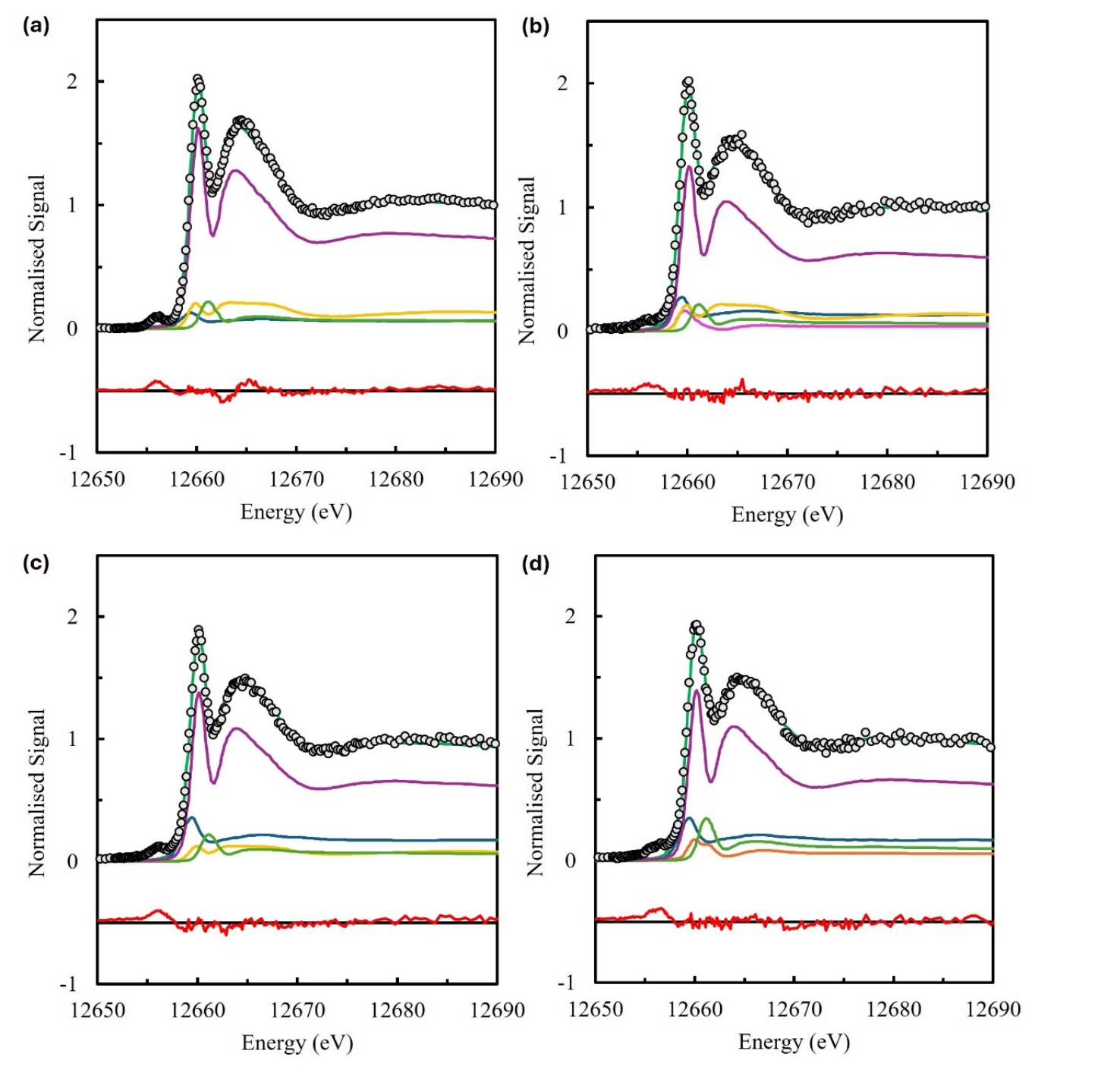


**Figure S29.** Linear combination analysis of Se Kα_1_-HERFD-XAS spectra of mouse **(a)** kidney, **(b)** whole blood, **(c)** liver, and **(d)** testis, from animals fed 5 mg Se/kg as SeN. The experimental data (points, grey) and the linear combination fit (green line) are displayed, along with components used in the best fit, scaled by their contributions to the fit: elemental Se, magenta line; SeMet (R–Se–R’), green line; CysSe^−^ (R–Se^−^), yellow line; CysSeSeCys (R–S–Se–Se–S–R’), orange line; CuSe (metal-selenide model), dark blue line; and SeN (reduced, (Se=C(NR_2_)_2_)), purple line. The lower red trace shows the fit residual. Fit fractions for all components are provided in **Table 1**.


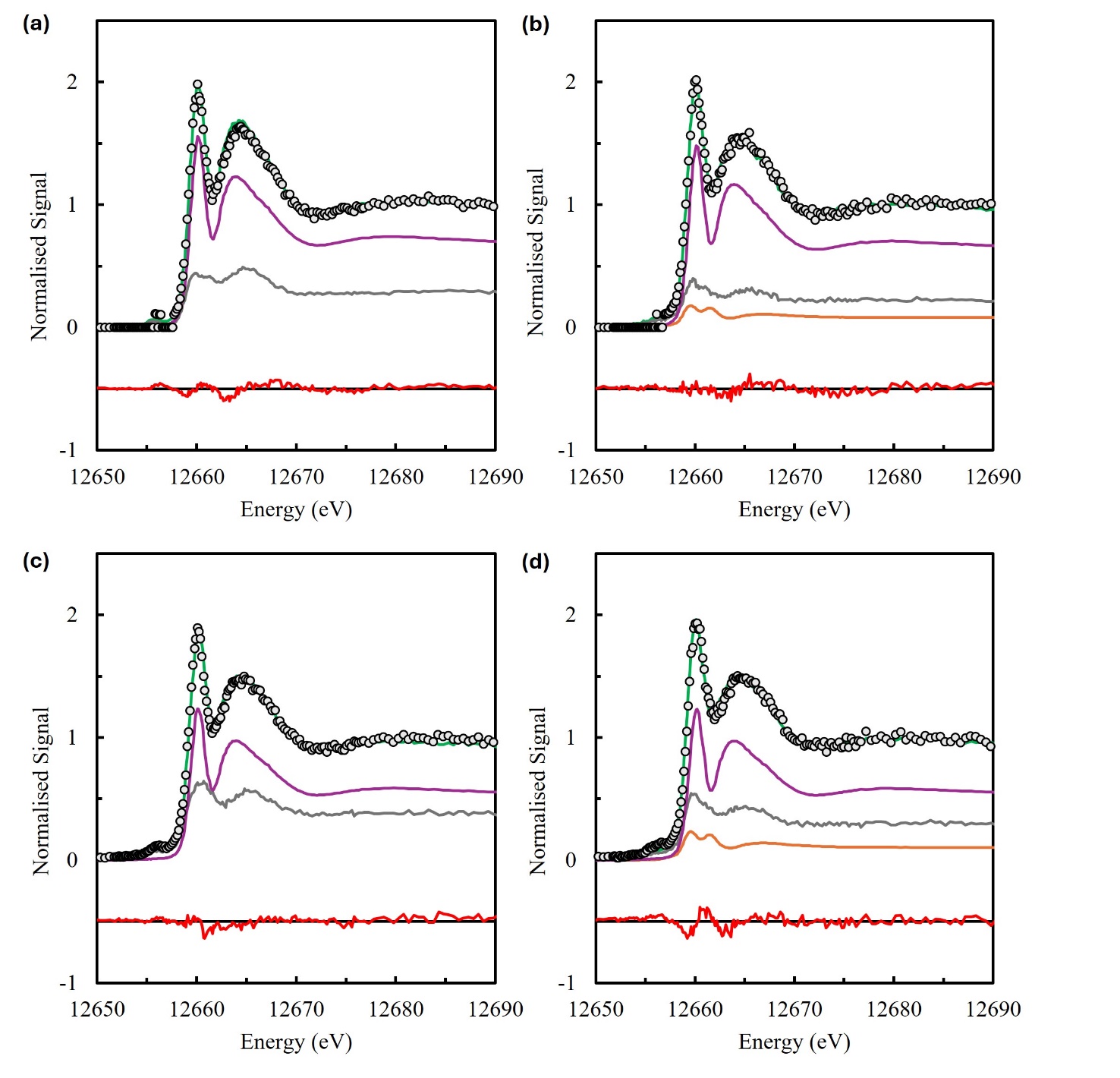


**Figure S30.** Linear combination analysis of Se Kα_1_-HERFD-XAS spectra of mouse **(a)** kidney, **(b)** whole blood, **(c)** liver, and **(d)** testis, from animals fed 5 mg Se/kg as SeN. The experimental data (points, grey) and the linear combination fit (green line) are displayed, along with components used in the best fit, scaled by their contributions to the fit: control tissue sample spectra, grey line; SeN (oxidised, R–Se–Se–R), orange line; and SeN (reduced, (Se=C(NR_2_)_2_)), purple line. The lower red trace shows the fit residual. Fit fractions for all components are provided in **Table 2**.
